# Common and rare variant analyses implicate *JARID2* in cerebral tau deposition

**DOI:** 10.1038/s44400-026-00107-6

**Published:** 2026-07-03

**Authors:** Tamil Iniyan Gunasekaran, Devendra Meena, Annie J. Lee, Siwei Wu, Logan Dumitrescu, Reisa Sperling, Timothy J. Hohman, Jingxian Huang, A4 Study, Alzheimer’s Disease Sequencing Project (ADSP), Abbas Dehghan, Ioanna Tzoulaki, Richard Mayeux, Badri Vardarajan

**Affiliations:** 1 The Gertrude H. Sergievsky Center, College of Physicians and Surgeons, Columbia University, New York, NY USA; 2 Department of Epidemiology and Biostatistics, School of Public Health, Imperial College London, London, UK; 3 Human Genetics Unit, Indian Statistical Institute, Kolkata, India; 4 Department of Neurology, College of Physicians and Surgeons, Columbia University and the New York Presbyterian Hospital, New York, NY USA; 5 Department of Neurology, The New York Presbyterian Hospital, New York, NY USA; 6 Taub Institute for Research on Alzheimer’s Disease and the Aging Brain, College of Physicians and Surgeons, Columbia University, New York, NY USA; 7 Vanderbilt Memory and Alzheimer’s Center, Vanderbilt University Medical Center, Nashville, TN USA; 8 Vanderbilt Genetics Institute, Vanderbilt University Medical Center, Nashville, TN USA; 9 Department of Neurology, Massachusetts General Hospital, Boston, MA USA; 10 Center for Alzheimer Research and Treatment, Department of Neurology, Brigham and Women’s Hospital, Boston, MA USA; 11 BHF Centre of Excellence, School of Public Health, Imperial College London, London, UK; 12 UK Dementia Research Institute, Imperial College London, London, UK; 13 Systems Biology, Biomedical Research Foundation of the Academy of Athens, Athens, Greece

**Keywords:** Diseases, Genetics, Neurology, Neuroscience

## Abstract

Genetic research on Alzheimer’s disease (AD) has primarily focused on amyloid-β (Aβ), with fewer studies exploring tau pathology. We performed common variant GWAS on tau-PET SUVRs from A4 (*n* = 311 preclinical AD) and ADNI (*n* = 375 across diagnostic groups) cohorts. We complemented this with locus-specific rare variant analyses in 1561 individuals across five cohorts. Genetic findings were evaluated using circulating plasma proteins from the UK Biobank Pharma Proteomics Project (*n* = 54,129). Polygenic risk scores (PRS) for tau and amyloid-SUVR were tested for association with AD. GWAS identified two loci: rs78636169 (*P* = 5.76 × 10^−10^) in *JARID2* and rs7292124 (*P* = 2.20 × 10^−8^) near *ISX*. Rare-variant analysis in *JARID2* revealed chr6:15257832:A:G (*P* = 7.08 × 10^−05^) in harmonized analysis and chr6:15492808:C:T (*P* = 1.65 × 10^−09^) in rare-variant meta-analysis. Pleiotropy analyses suggested limited overlap between tau- and amyloid-related genetic signals. Gene-based analysis highlighted *JARID2*, a component of the PRC2 multi-protein complex. Mendelian randomization analysis identified LRRFIP1, a protein that binds with PRC2, as potentially causally linked to tau pathology. Amyloid-PRS, but not tau-PRS, was associated with AD clinical status, with age-dependent effects in *APOE*-ε4 carriers. Leveraging both GWAS and a large rare-variant cohort, we identified *JARID2* as a candidate gene associated with tau pathology and observed patterns consistent with partially distinct roles of Aβ and tau in AD progression.

## Introduction

Alzheimer’s disease (AD) is a complex neurodegenerative disease characterized by the abnormal deposition of extracellular amyloid-β (Aβ) protein in neuritic plaques and intracellular hyperphosphorylated tau protein in neurofibrillary tangles within the brain^1^. Neurodegeneration in AD is accompanied by hyperphosphorylated forms of tau. Cognitive impairment may begin during early stages of tau accumulation in the medial temporal lobe, while the spread of tau pathology to the neocortex is associated with more severe cognitive decline and disease progression^2^. Despite the crucial role tau pathology plays in AD, the genetic risk factors and gene pathways underlying tau-mediated AD pathology remain unclear.

Genome-wide association studies (GWAS) of cerebral Aβ measures acquired from positron emission tomography (PET) images have identified several genetic loci, including *APOE*^3,4^*, BCHE*^4^*, IL1RAP*^5^*, CR1, ABCA7*, and *FERMT2*^3^. In our previous study, we conducted a large multi-ethnic meta-analysis of amyloid PET measures and identified a novel loci in the *RBFOX1* gene^6^. Unlike amyloid PET imaging, tau PET remains less widely available, reflecting not only later regulatory approval but also technical challenges associated with radioligands for imaging intracellular tau aggregates compared to extracellular amyloid plaques^7,8^. Studies combining amyloid PET, tau PET, and structural magnetic resonance imaging (MRI) have identified tau accumulation as a primary contributor to cognitive decline in AD. This underscores the importance of tau PET imaging in evaluating AD-associated cognitive decline and neurodegeneration^9–11^.

In a candidate gene study, single nucleotide polymorphisms (SNPs) in *BIN1* were associated with tau deposition^12^. Previous tau-PET GWAS studies have pinpointed a few genetic loci, including *PPP2R2B, IGF2BP3*^13^*, ZBTB20*, and *EYA4*^14^, associated with tau deposition in the brain. However, these tau PET GWASs were carried out using single cohorts with limited sample sizes.

In this study, we harmonized tau-PET measures from two cohorts and conducted a genome-wide meta-analysis of common variants to identify genetic loci associated with cerebral tau deposition. Building on these findings, we performed locus-specific rare-variant analyses to validate the rare-variant associations. Subsequently, we performed Mendelian randomization analysis of circulating plasma proteins in UK Biobank to identify proteins whose cognate genes may have causal link to tau deposition. We hypothesized that common genetic pathways could drive amyloid and tau pathologies in AD progression. Therefore, we analyzed genes, and their enriched pathways associated with both amyloid and tau pathologies. We also evaluated the predictive accuracy of tau polygenic scores (PRS) and amyloid PRS for clinical and pathological AD status.

## Results

### Tau-PET GWAS identifies the *JARID2* locus

**Fig. 1:**
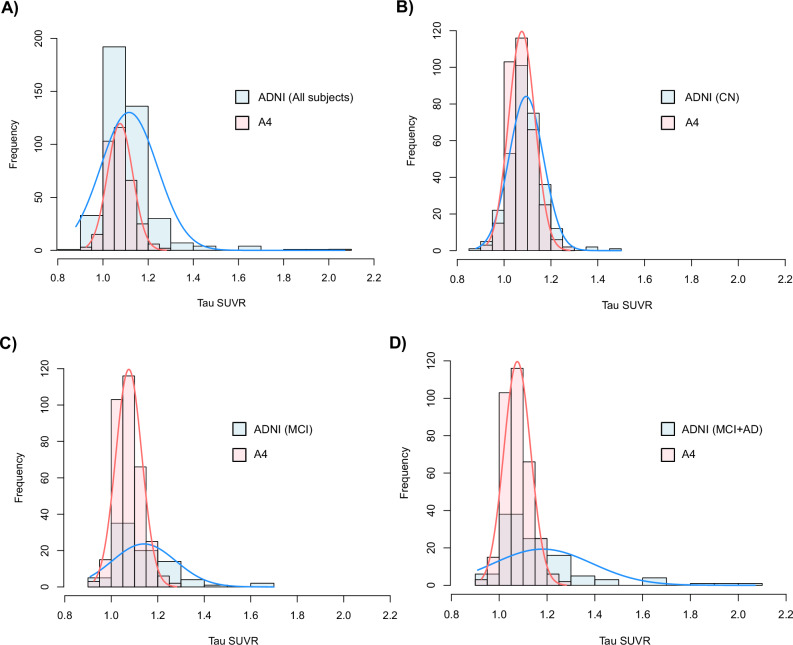
Tau standard uptake value ratios (SUVRs) histograms for ADNI and A4 cohort individuals. **A** All individuals from both cohorts. **B** Cognitively normal (CN) individuals from ADNI and all individuals from A4. **C** Mildly cognitively impaired (MCI) individuals from ADNI and all individuals from A4. **D** MCI and Alzheimer’s disease (AD) individuals from ADNI compared with all individuals from A4. The solid lines in the over the histograms denotes the estimated density curve. The red line indicates A4 cohort and the blue line indicates ADNI cohort.

The tau SUVR distribution in the ADNI cohort was right-skewed compared to that of A4 cohort (Fig. 1A–D). Mildly cognitively impaired (MCI) and AD patients in ADNI had higher levels of tau-deposition compared to cognitively normal (CN) in ADNI and A4 participants, who had similar levels. We used clinical AD as a covariate for the ADNI dataset, while no such adjustments were made for A4.

**Fig. 2:**
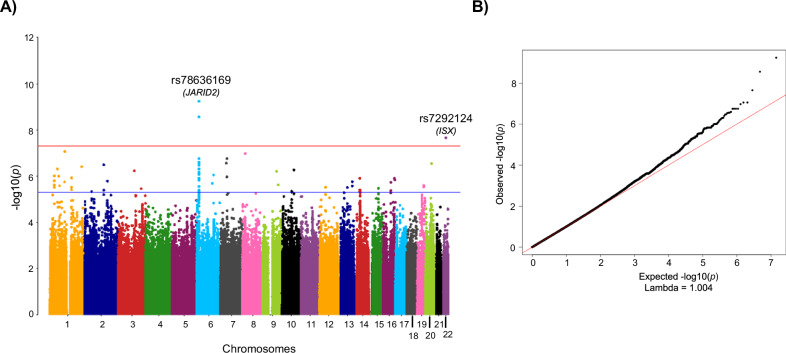
SNPs associated with cerebral tau deposition. **A** Manhattan plot showing meta-analysis *P* values (depicted on the –log_10_ scale) from linear regression on cerebral tau deposition involving non-Hispanic White (NHW) subjects. The threshold for genome-wide significance is represented by a red line at *P* = 5 × 10^−8^, while suggestive significance is indicated by a blue line at *P* = 5 × 10^−6^ threshold. **B** Quantile-Quantile (QQ) plots for the SNPs associated with cerebral tau deposition involving multi-ethnic subjects. The QQ plot showed no spurious genomic inflation (*λ* = 1.004).

**Fig. 3:**
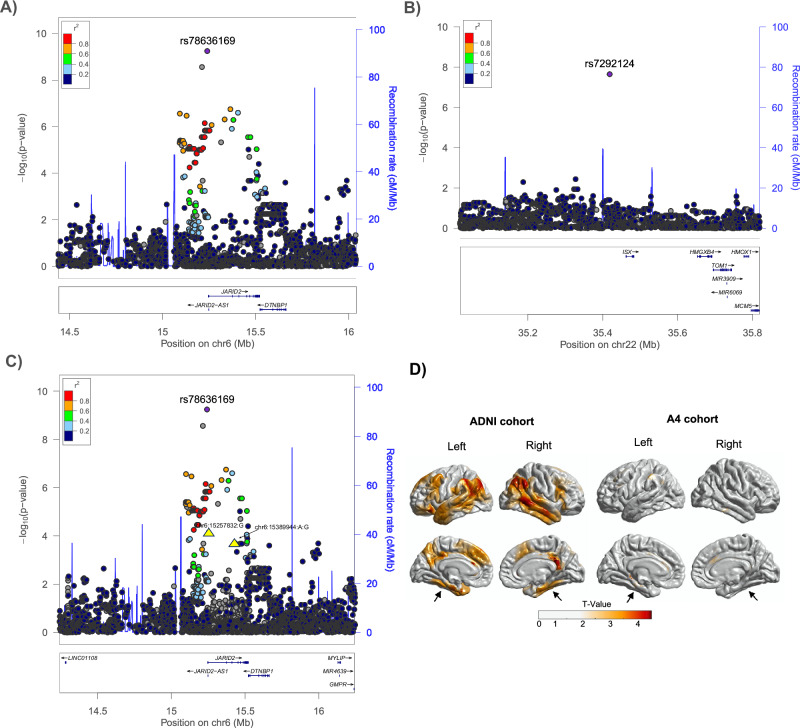
Regional plots showing tau associated SNPs and the brain cortical atrophy patterns. **A** Magnified LocusZoom regional association plots show the regions and common variants around rs78636169 in the *JARID2* loci and **B** rs7292124 near *ISX* loci with common variants. **C** Rare and common variants around rs78636169 in the *JARID2* loci. The purple dot indicates the most significant SNP in the region and the yellow triangle shows the most significant rare variants from harmonized tau-SUVR analysis. **D** Brain cortical atrophy associated with rs78636169 in *JARID2*. Brian cortical patterns associated with rs78636169 represented in t-value. rs78636169 AA + AG carriers were compared with GG carriers to infer the cortical atrophy in ADNI cohort (left) and A4 cohort (right). The black arrow indicates greater atrophy in the parahippocampal region among rs78636169 AA + AG carriers in the ADNI cohort, while mild atrophy was observed in the same region among individuals from the A4 cohort.

The SNP-based genome-wide meta-analysis of non-Hispanic White (NHW) individuals from A4 (N = 311) and ADNI (N = 375) (Supplementary Tables 1 and 2), identified a significant association of rs78636169 (*P* = 5.76 × 10^−10^) near the *JARID2* (Jumonji and AT-Rich Interaction Domain containing 2) gene on chromosome 6 with tau deposition (Figs. 2A, B, and 3A, B, Table 1, and Supplementary Table 3). The association remained genome-wide significant when we included all multi-ethnic individuals (Supplementary Fig. 1 and Supplementary Table 4).

**Table 1:** Genome-wide significant associations with tau deposition among non-Hispanic White participants

SNP	Nearest gene	Chr	BP	Variant type	A1	ADNI cohort				A4 cohort				Tau-SUVR Meta-analysis*					
						*β*	SE	*P* value	MAF	β	SE	*P*-value	MAF	*β*	SE	*P* value	Dir	Het ChiSq	Het *P* val
rs78636169	*JARID2*	6	15242005	Upstream variant	A	0.73	0.17	1.66E-05	0.04	0.7	0.16	1.44E-05	0.06	0.71	0.12	5.76E-10	++	0.01	0.9
rs200751686	*JARID2*	6	15213555	Intergenic region	CA	0.66	0.16	6.70E-05	0.05	0.7	0.16	1.65E-05	0.06	0.68	0.11	2.74E-09	++	0.03	0.86
rs7292124	*ISX*	22	35419122	Intergenic region	C	0.77	0.16	1.75E-06	0.05	0.58	0.2	4.39E-03	0.04	0.69	0.12	2.20E-08	++	0.53	0.46

*SNP* single nucleotide polymorphism, *Chr* chromosome, *BP* base pair location (GRCh37), *A1* effect allele, *SE* standard error, *MAF* minor allele frequency, *Dir* Effect direction, *Het ChiSq* Chi-square value for heterogeneity test, *Het PVal* *P* value for heterogeneity in effect sizes in meta-analysis.

*P value of 5E-08 was used to assess genome-wide significance in the meta-analysis.

Regulatory annotation indicated that rs79670124 in *JARID2* is located within a promoter region and is in strong linkage disequilibrium (*r²* > 0.9) with rs78636169. Expression analyses using the GTEx database (https://www.gtexportal.org/)^15^ showed that *JARID2* is highly expressed in brain tissues, and both rs78636169 and rs79670124 are associated with reduced *JARID2* expression in whole blood (Supplementary Fig. 2). These findings suggest a potential role for *JARID2* in neurodegeneration.

In addition, rs7292124 (*P* = 2.20 × 10^−8^), near the *ISX* (Intestine Specific Homeobox) gene on chromosome 22, was also genome-wide significant in NHW. These associations remained significant after adjustment for *APOE*-ε4 status, (rs78636169, *P* = 1.20 × 10^−8^ and rs7292124, *P* = 5.17 × 10^−9^). Additionally, rs112518363 (*P* = 3.51 × 10^−8^) near *INTS10* (Integrator Complex Subunit 10) became genome-wide significant when adjusted for *APOE* ε4 status.

In a recently published large tau-PET GWAS^16^, rs918804 near *RMDN2* showed the strongest genome-wide significant association (*P* < 5 × 10^−8^) with tau deposition. Consistently, in our study, rs918804 was also associated with global tau PET SUVR levels (*P* = 4.70 × 10^−3^). Likewise, the same GWAS reported rs78636169 (*P* = 2.77 × 10^−3^) in *JARID2* as significantly associated with tau deposition, which we also observed in our analysis.

### Association of rare variants in the *JARID2* gene with tau deposition

Following the discovery of *JARID2* in the genome-wide common variant analysis, we conducted rare-variant analyses in the *JARID2* gene region using harmonized tau-SUVR. In the large tau-PET WGS cohort, an intronic variant, chr6:15257832:A:G showed strong association (*P* = 7.08 × 10^−05^) with tau deposition (Fig. 3C). Additionally, nine other intronic variants were associated (*P* < 0.01) with tau deposition (Supplementary Fig. 3, Supplementary Tables 5 and 6).

In the rare-variant meta-analysis across multiple cohorts, another intronic variant, chr6:15492808:C:T in *JARID2* reached genome-wide significance for tau deposition (*P* = 1.65 × 10^−09^). Twenty-two additional intronic variants showed association (*P* < 0.01), including four that achieved genome-wide significance (*P* < 5 × 10^−08^) (Supplementary Table 7). Furthermore, a missense variant, chr6:15520178:G:C in *JARID2* was significantly associated with tau deposition (*P* = 0.02) in the meta-analysis (Supplementary Table 8).

### Relationship of tau-associated SNPs with amyloid deposition and clinical Alzheimer’s disease

Amongst 41,455 SNPs nominally associated (*P* < 0.005) with tau deposition, we identified 20 genetic loci that are also associated (*P* < 0.005) with amyloid-SUVR. Sixteen SNPs had the same direction of effect for both amyloid and tau deposition including SNPs related to *PVRL2* (Poliovirus receptor-related 2) and *APP* (Amyloid Precursor Protein) (Supplementary Table 9). Amongst 75 genetic loci associated for AD risk in a previous large-scale GWAS of clinical AD^17^, four variants in *SNORA73* (rs76928645), *INPP5D* (rs10933431), *ABCA7* (rs12151021) and *IL34* (rs4985556) were nominally associated (*P* < 0.05) with tau pathology (Supplementary Table 10).

Additionally, we evaluated associations with AD risk and amyloid deposition for all variants within *JARID2*, *ISX*, and *INTS10*, given that genome-wide significant loci for tau deposition were identified in proximity to these genes. Across these loci, 65 variants showed nominal associations (*P* < 0.05) with AD risk, and seven variants were nominally associated (*P* < 0.05) with amyloid accumulation. Notably, rs17391774 in *JARID2* demonstrated nominal associations with both tau and amyloid deposition, while rs7745729 in *JARID2* was nominally associated with both tau deposition and AD risk (Supplementary Table 11). These findings suggest limited but detectable overlaps across tau, amyloid, and AD-related genetic signals.

Additionally, we evaluated the association with AD risk and amyloid deposition of all variants within *JARID2*, *ISX*, and *INTS10*, given that genome-wide significant loci for tau deposition were identified in proximity to these genes. Across these loci, 65 variants showed nominal association (P < 0.05) with AD risk, and seven variants (P < 0.05) with amyloid accumulation. Notably, rs17391774 in *JARID2* demonstrated nominal association with both tau and amyloid deposition, while rs7745729 in *JARID2* was nominally associated with both tau deposition and AD risk (Supplementary Table 11). These findings suggest detectable but limited overlap across tau, amyloid, and AD-related genetic signals.

### Gene level association with tau deposition

Gene-based meta-analysis using MAGMA on tau-SUVR identified *APOE* (*P* = 2.55 × 10^−6^) on chromosome 19 and *CTNNA3* (*P* = 2.86 × 10^−6^) on chromosome 10 (Supplementary Table 12). *JARID2* had a suggestive association with tau deposition (*P* = 1.22 × 10^−4^). Additionally, we conducted a gene-based analysis with amyloid deposition using summary statistics from our previous study^6^. In addition, we identified 61 genes that were nominally significant association (*P* < 0.05) with both tau and amyloid deposition including *APOE*, *TOMM40*, and *COL5A2* (*P* < 0.005) with both tau and amyloid pathologies (Supplementary Fig. 4 and Supplementary Table 13). *JARID2* was also nominally significant (*P* = 0.047) for association with Tau deposition in an independent tau-PET GWAS dataset^13^ (Supplementary Table 14**)**.

### Mendelian randomization of circulating plasma proteins levels with tau deposition

Of the 916 genes nominally associated with tau SUVR levels, 84 overlapped with the UKBPPP and were used for the *cis*-MR analysis. Since JARID2 levels were not directly measured, we used other proteins in the PRC2 complex to confirm a causal link to Tau pathology. MR analysis of circulating plasma proteins found evidence of a potential causal effect of genetically predicted LRRFIP1 (β_Wald-ratio_ = −2.64 [−4.46, −1.21], p = 0.016), HADH (β_Wald-ratio_ = 3.47 [1.47, 5.47], p = 0.019), IST1 (β_Wald-ratio_ = −2.26 [−3.51, −1.01], p = 0.016), and PRTG (β_IVW_ = −0.42 [−0.68, −0.15], p = 0.04) on tau-PET levels (Supplementary Fig. 5, Table 2 and Supplementary Table 15). Among these, HADH showed suggestive evidence of co-localization (PPH4 = 61.6%) while LRRFIP1 and IST1 indicated limited power in colocalization to differentiate between a true causal variant or horizontal pleiotropy (Supplementary Fig. 6 and Supplementary Tables 16 and 17).

**Table 2:** Mendelian Randomization identified proteins with potential causal effect on Tau PET levels.

Mendelian Randomization analysis										Gene-based meta-analysis on non-Hispanic Whites showing the association with tau deposition			
Protein	Method	Panel	NSNP	Beta	SE	OR (LCI-UCI)	*P* value	FDR	GENE	Condition analysis 1*		Condition analysis 2^#^	
										ZSTAT	*P* value	ZSTAT	*P* value
LRRFIP1	Inverse variance weighted	Inflammation II	2	−2.6353	0.7250	0.07 (0.02–0.3)	2.78E-04	0.016	*LRRFIP1*	1.6247	0.05	2.6126	4.49E-03
IST1	Wald ratio	Inflammation II	1	−2.2590	0.6372	0.1 (0.03–0.36)	3.93E-04	0.016	*IST1*	2.8723	2.04E-03	2.3256	0.01
HADH	Wald ratio	Inflammation	1	3.4662	1.0206	32.01 (4.33–236.62)	6.83E-04	0.019	*HADH*	1.7522	0.04	2.741	3.06E-03
PRTG	Inverse variance weighted	Oncology	4	−0.4152	0.1348	0.66 (0.51–0.86)	2.07E-03	0.044	*PRTG*	1.5565	0.06	2.0335	0.02
PRTG	Weighted median	Cardiometabolic	4	−0.3959	0.1413	0.67 (0.51–0.89)	5.07E-03	NA					
PRTG	MR Egger	Oncology	4	−0.5782	0.3128	0.56 (0.3–1.04)	0.21	NA					

*MR Egger* Mendelian Randomization-Egger, NSNP number of single nucleotide polymorphism, *SE* standard error, *OR* Odds Ratio, *LCI* lower confidence interval, *UCI* upper confidence interval, *FDR* false discovery rate, *NA* not applicable, *ZSTAT* Z statistics.

*Condition analysis 1: adjusted for age, sex, three principal components and clinical diagnosis status.

^#^Condition analysis 2: adjusted for age, sex, three principal components, *APOE* ε4 status and clinical diagnosis status.

### Pathways associated with tau and amyloid deposition

Pathway analysis revealed that genes associated with tau deposition were enriched in pathways related to the positive regulation of sterol and cholesterol transport, fascia adherens, postsynaptic specialization, and asymmetric synapse (*q* value < 0.05) (Supplementary Fig. 7 and Supplementary Table 18).

Amongst genes showing significant associations with both tau and amyloid, no gene pathways remained significantly associated (*q*-value < 0.05) after multiple corrections, but endocytosis and signal transduction pathways were nominally significant (Supplementary Fig. 8 and Supplementary Table 19).

### Association of tau and amyloid PRS with clinical AD and neuropathological measures

In ADNI and A4 participants, we found a robust association of AD PRS with amyloid-SUVR levels (*P* = 1.94 × 10^−11^; *R*^2^ = 0.079) but only a weak association with tau-SUVR (*P* = 0.03; *R*^2^ = 0.0087). Summary statistics from a recently published large-scale tau-PET GWAS^16^ were evaluated for association with AD PRS. Consistent with our findings, tau showed only a weak association with AD PRS (*P* = 0.007; *R*^2^ = 0.004) compared to amyloid.

Next, we analyzed the association of tau and amyloid-SUVR PRS with the clinical and pathological diagnosis of AD^18^ in the ROSMAP cohort. Results showed a significant association between amyloid PRS and clinical AD status (*P* = 0.018; *R*^2^ = 0.0038), but not with tau PRS (*P* = 0.29; *R*^2^ = 0.00076). However, tau PRS was significantly associated with clinical AD status among *APOE*-ε4 carriers (*P* = 0.015; *R*^2^ = 0.017). In non-ε4 carriers, neither amyloid nor tau PRS were associated with clinical AD. Neither amyloid PRS nor tau PRS was associated with pathological AD status (Supplementary Table 20).

### Effects of rs78636169 on brain cortical atrophy

We analyzed the effects of rs78636169 SNP from the *JARID2* gene on brain cortical atrophy under an additive genetic model. In ADNI, which included individuals with clinical diagnoses ranging CN, MCI and AD, the results revealed cortical atrophy patterns in the entorhinal cortex, parahippocampal region, posterior cingulate, inferior parietal lobe, precuneus, middle temporal gyrus, inferior temporal gyrus, and superior frontal region. Additionally, the atrophy pattern was asymmetrical in some regions (Fig. 3D). Analysis of the A4 study cohort, which comprised pre-clinical individuals, showed cortical atrophy patterns in fewer regions, including parahippocampal, anterior cingulate and inferior parietal regions (Fig. 3D).

Mediation analyses were performed to evaluate whether tau SUVR mediates the association between rs78636169 and cortical thickness across Braak stage regions in the ADNI and A4 cohorts. In the ADNI cohort, significant indirect mediation effect of Tau-SUVR on rs78636169 were observed across multiple Braak stages, supporting a tau-mediated cortical atrophy linking rs78636169. Specifically, significant mediation effects were detected in Braak stages 1–2 (*β* = −0.109; *P* = 4.0 × 10^−4^), 3–4 (*β* = −0.032, *P* = 1.2 × 10^−3^), and 5-6 (*β* = −0.017, *P* = 1.6 × 10^−3^), as well as in the combined Braak 1–6 regions (*β* = −0.053, *P* = 4.0 × 10^−4^). The total effect, representing the overall association between rs78636169 and cortical atrophy, was also significant in Braak stages 1–2, 3-4, and Braak 1–6 (*P* < 0.01), whereas the direct effects, representing the association between rs78636169 and cortical atrophy independent of tau, were not statistically significant across regions (all *P* > 0.05). The direction of effects in ADNI indicated that the rs78636169 risk allele is associated with increased tau burden, which in turn relates to reduced cortical thickness, consistent with a predominantly indirect (tau-mediated) effect. The direct effect of rs78636169 on cortical atrophy was not statistically significant (Supplementary Table 21).

In the A4 cohort, there was no evidence of significant mediation by tau SUVR across any Braak stage. The indirect mediation effects were not statistically significant (*P* > 0.05) for Braak stages 1–2, 3-4, 5-6, or the combined Braak 1–6 regions. Similarly, neither the direct effects nor the total effects reached statistical significance across all regions (*P* > 0.05), indicating a lack of detectable mediation or overall association in this cohort (Supplementary Table 22). Since all subjects in the A4 cohort were preclinical, the distribution of cortical thickness more closely resembles that of CN individuals in ADNI, rather than those with MCI or AD, which may partly explain the lack of detectable mediation effects in A4 (Supplementary Fig. 9). These results were also in line with the whole brain atrophy results (Fig. 3D).

## Discussion

In this study, we conducted a genome-wide common variant meta-analysis using tau-SUVR measures derived from ^[18]^F-flortaucipir PET images from the A4 and ADNI study cohorts. Among 686 non-Hispanic White individuals, we identified two significant SNPs: rs78636169 (*P* = 5.76 × 10^−10^) near *JARID2* and rs7292124 (*P* = 2.20 × 10^−08^) near the *ISX* gene, associated with cerebral tau deposition. *JARID2* remained genome-wide significant when 47 multi-ethnic individuals were included and when adjusted for *APOE*-ε4 dosage, suggesting *APOE*-ε4 has no significant influence on these loci.

Building on this discovery, we conducted a large-scale locus-specific rare-variant analysis in the *JARID2* region across multiple cohorts, which revealed additional signals of strong association with tau deposition. These included chr6:15257832:A:G (β = 0.28, *P* = 7.08 × 10^−05^) in the harmonized tau-SUVR analysis and chr6:15492808:C:T (β = −1.51, *P* = 1.65 × 10^−09^) in the rare-variant meta-analysis, along with several other intronic variants that achieved genome-wide significance. Additional analyses using the GTEx database, together with regulatory annotations, suggest that variants in *JARID2* may influence gene expression. The convergence of evidence from both common and rare-variant analyses highlights *JARID2* as a robust and novel genetic locus influencing tau pathology. Importantly, pleiotropy analyses showed limited evidence of overlap between *JARID2* variants and amyloid burden or AD risk. While most variants did not demonstrate strong cross-trait associations, we observed nominal associations (*P* < 0.05) for several variants with AD risk and amyloid burden, including rs17391774 and rs7745729 in *JARID2*, which also showed associations with tau deposition. These findings are consistent with a predominantly tau-related signal. However, given the modest sample size, they should be interpreted cautiously, and shared genetic architecture with amyloid-related traits cannot be fully excluded.

Gene-based analyses identified *APOE* and *CTNNA3*, while *JARID2* showed suggestive significance. Our study reinforces the association of *APOE* with both amyloid tau deposition^4,6,19^,.

*JARID2* (Jumonji, AT-rich interactive domain 2) is a protein-coding gene involved in regulating gene expression and chromatin structure^20^. JARID2 is also a component of PRC2 (Polycomb Repressive Complex 2) multi-protein complex, necessary for transcriptional silencing through histone modification^21^. PRC2 methylates JARID2, promoting PRC2 activity by guiding it to target genomic regions^21,22^. PRC2 is essential for processes like cell differentiation, proliferation, and maintaining stem-cell plasticity. With age, *PRC2* expression decreases in the brain, leading to abnormal expression of genes linked to AD^23^. Neuronal deficiency in *PRC2* is also associated with progressive neurodegeneration in mice^24^. Overall, *JARID2* and *PRC2* multi-protein complex play roles in tau pathology and neurodegeneration in AD, though further validation is needed.

To establish potential causal relationship between circulating plasma protein levels of the top genetic hits, we conducted MR analysis using the UKBPPP cohort. Since JARID2 protein levels were not measured directly, we assessed constituent proteins in the PRC2 complex. MR analysis revealed potential causal links of genetically predicted LRRFIP1 protein levels to lower tau deposition. *LRRFIP1*, along with *PRC2* multi-protein complex, is involved in the downregulation of tumor necrosis factor-α (TNF-α)^25^, a pro-inflammatory cytokine involved in regulating innate and adaptive immunity, playing an important role in AD pathology. Inhibiting TNF-α has demonstrated a protective effect against AD pathology, including Aβ and tau deposition^26,27^. Our analysis suggests *JARID2, LRRFIP1* and other proteins in the *PRC2* multi-protein complex plays a critical role in protecting against tau pathology. Despite this biological plausibility, colocalization evidence was limited for several proteins, raising the possibility that the observed associations may reflect shared loci or horizontal pleiotropy rather than a direct causal effect of circulating protein levels on tau deposition. Accordingly, these findings should be interpreted cautiously as hypothesis-generating, prioritizing PRC2-associated proteins for further validation rather than establishing causality. Larger cohorts will be required to replicate and extend these MR and colocalization analyses.

*CTNNA3* (α-3 catenin) forms the α-3/β-1 catenin complex with *CTNNB1* (β-1 catenin), binding to *PSEN1* (presenilin-1) and promoting higher Aβ42 levels^28,29^, linked to the early onset of familial AD^30,31^. *CTNNA3* is also associated with AD, particularly in females^32^ and Caribbean-Hispanics^33^. In this study, *CTNNA3* shows an *APOE*-dependent association with tau deposition, consistent with previous studies^34^.

In addition, we examined variation at the *MAPT* (Microtubule Associated Protein Tau) locus, given the central role of tau protein pathology in AD and the established involvement of MAPT variants in primary tauopathies^35–37^. We identified sixteen SNPs nominally associated with tau deposition, including rs63750072 (Supplementary Table 23), a missense variant previously reported in AD-related studies^38,39^.

SNPs from four AD-associated loci: *SNORA73, INPP5D, ABCA7*, and *IL34* showed nominal associations (*P* < 0.05) with tau pathology. The roles of *ABCA7* (ATP Binding Cassette Subfamily A Member 7)^40,41^, *IL34* (Interleukin 34)^42,43^ and *INPP5D* (Inositol Polyphosphate-5-Phosphatase D)^44,45^ in Aβ-mediated AD pathology have been well-studied. Our findings suggest a potential link between these genes and tau and amyloid pathologies.

Gene-based analysis from amyloid-PET and tau-PET GWASs identified 61 genes associated (*P* < 0.05) with both amyloid and tau pathology. *APOE, TOMM40* and *COL5A2* showed robust associations (*P* < 0.005). *COL5A2*, particularly noteworthy, is linked to reduced neuronal energy supply, leading to AD-related apoptosis^46^. No previous studies have explored genes associated with both tau and amyloid pathologies using amyloid-PET and tau-PET GWAS results.

Gene pathway analysis revealed tau-associated genes enriched in “positive regulation of sterol” and “cholesterol transport”, “fascia adherens”, “postsynaptic specialization”, and “asymmetric synapse” pathways. Positive regulation of sterol and cholesterol is crucial for cellular homeostasis, especially lipid metabolism^47^. Disturbed cholesterol homeostasis in neuronal cells is observed in AD, contributing to tau-related pathogenesis^46^. Disruptions in postsynaptic specialization may lead to synaptic dysfunction in AD^48^. Asymmetric synapses, or excitatory synapses, are activated by neurotransmitter release^49^. Aβ co-localizes with postsynaptic densities, contributing to the loss of excitatory synapses in AD^50^, suggesting tau-associated gene pathways likely contribute to synaptic dysfunctions.

AD PRS was strongly associated with amyloid-SUVR compared to tau, indicating that AD-associated genetic loci are linked strongly to Aβ pathology but not tau. Amyloid-SUVR PRS was significantly associated with clinical AD, whereas tau PRS showed no association, suggesting different genetic pathways for Aβ and tau in AD pathology. Among *APOE* ε4 carriers, Aβ seems crucial in early AD stages, while tau plays a role later in the middle and late stages of the disease. Hippocampal sclerosis, likely mediated by tau, occurs specifically among *APOE* non-ε4 carriers, though its association with tau PRS showed only a trend towards significance.

Cortical atrophy was associated with rs78636169 in *JARID2* in both ADNI and A4 cohorts. ADNI results showed rs78636169-A allele mediated cortical atrophy in Braak stage 1–6 regions^51^. Conversely, pre-clinical AD participants in the A4 cohort showed early signs of cortical atrophy in Braak stage 1, representing early tau accumulation stage. Previous studies demonstrated tau accumulation following Braak stages^52^, and share a similar topography with cortical atrophy patterns^53^. Consistent with prior findings, our results suggest that rs78636169-associated tau accumulation may contribute to cortical atrophy in similar topographical regions, supported by mediation analyses across Braak stage regions.

Although our common variant genome-wide meta-analysis was limited by modest sample size, we reinforced these findings through a large-scale, multi-cohort locus-specific rare-variant meta-analysis and a mega-analysis with harmonized tau-PET phenotypes across all cohorts. Power analyses using QUANTO software^54^ indicate that, at genome-wide significance (*α* = 5 × 10^−8^), our sample size (*N* = 686) among non-Hispanic White individuals is primarily powered (>80%) to detect higher-frequency variants with relatively large effect sizes, while remaining underpowered for lower-frequency variants or those with more modest effects. The convergence of evidence from both common and rare variants underscores the robustness and generalizability of our results. Importantly, we identified *JARID2*, a core component of the PRC2 multi-protein complex, and *CTNNA3* as novel genetic loci associated with tau deposition. Mendelian randomization further implicated the PRC2 complex as a causal driver of tau pathology, while cortical analyses revealed *JARID2*-mediated atrophy that closely followed Braak staging. In addition, we detected associations with *COL5A2* and pathways bridging amyloid and tau biology, pointing to interconnected yet distinct mechanisms. Consistent with prior studies, tau may initiate local neurodegeneration, whereas Aβ-related neurodegeneration appears to depend, at least in part, on its interaction with tau in AD^55,56^. Our findings align with this framework, suggesting partially distinct, though potentially overlapping, genetic influences on tau and amyloid-related processes.

Despite these strengths, the molecular mechanisms linking JARID-PRC2 activity to tau pathology remain to be elucidated. One compelling hypothesis is that PRC2-mediated repression normally suppresses *MAPT* transcription in adult neurons^57^, a plausible mechanism given that *MAPT* expression is developmentally regulated and largely silenced postnatally through epigenetic mechanisms^58–60^. Under this model, loss-of-function *JARID2* variants could relax PRC2*-*dependent repression at the *MAPT* locus, leading to aberrant re-expression or dysregulation of tau, thereby directly driving pathological accumulation. A parallel pathway may involve PRC2-dependent regulation of broader neuronal gene programs, including *BDNF*^61–63^, a key mediator of synaptic plasticity^64,65^ and neuronal resilience^61^. Altered *BDNF* signaling has been shown to influence tau phosphorylation dynamics^66,67^, raising the possibility that *JARID2* variants contribute to neuronal vulnerability through this axis as well.

Future studies integrating cell-type specific epigenomic profiling, animal models, and transcriptomic analyses will be essential to determine whether the JARID2-PRC2 complex directly regulates *MAPT* or other neuronal genes or acts through intermediate pathways such as *BDNF*. Such approaches will help clarify the causal chain linking epigenetic regulation to tau pathology and refine the biological interpretation of the genetic signals identified in this study.

Taken together, the findings here offer valuable insights into tau pathology mediated by the JARID2 and PRC2 complex, while not excluding shared mechanisms with amyloid-related pathways. These results highlight candidate genetic loci associated with tau and amyloid in AD progression and provide a foundation for future studies aimed at elucidating underlying biological mechanisms and potential therapeutic strategies.

## Methods

### Study participants

We obtained data from the Anti-Amyloid Treatment in Asymptomatic Alzheimer’s Disease (A4) study and the Alzheimer’s Disease Neuroimaging Initiative (ADNI) study, which are available in the ADNI database (http://adni.loni.usc.edu). Our analysis included data from 330 pre-clinical individuals from the A4, together with 303 cognitively normal (CN) individuals, 81 individuals with mild cognitive impairment (MCI), and 19 individuals with diagnosed AD from the ADNI. In ADNI, the clinical diagnosis of probable AD was based on the National Institute of Neurological Disorders and Stroke/Alzheimer’s Disease and Related Disorders Association (NINDS-ADRDA) criteria^68^. Eligibility criteria for participation in the ADNI study are described elsewhere^68^. Detailed information about the A4 study participants can be found in our previous study^6^. Demographic characteristics of the study participants are presented in Table 3.

**Table 3:** Demographic characteristic of multi-ethnic population and non-Hispanic Whites for Tau PET from ADNI and A4 cohorts

Demographic information	ADNI						A4	
	All ethnicity			Non-Hispanic White			All ethnicity	Non-Hispanic White
Clinically diagnosed sample groups	CN	MCI	AD	CN	MCI	AD	Pre-clinical AD	Pre-clinical AD
*N*	303	81	19	280	76	19	330	311
Age (y, mean ± SD)	71.43 ± 5.94	69.96 ± 7.62	73.79 ± 10.75	71.64 ± 5.87	70.28 ± 7.61	73.79 ± 10.75	78.41 ± 4.76	71.76 ± 4.76
Female (*n*, %)	184 (61%)	32 (39%)	7 (37%)	164 (59%)	28 (37%)	7 (37%)	206 (62%)	193 (62%)
Years of education (y, mean ± SD)	16.81 ± 2.38	16.36 ± 2.60	16.16 ± 2.75	16.82 ± 2.35	16.41 ± 2.63	16.16 ± 2.75	16.34 ± 2.73	16.33 ± 2.72
MMSE (mean ± SD)	29.18 ± 1.08	28.26 ± 1.51	22.68 ± 2.31	29.18 ± 1.10	28.24 ± 1.51	22.68 ± 2.31	28.73 ± 1.31	28.74 ± 1.29
Tau PET SUVR (mean ± SD)	1.09 ± 0.07	1.14 ± 0.14	1.36 ± 0.33	1.10 ± 0.07	1.14 ± 0.14	1.36 ± 0.33	1.08 ± 0.06	1.08 ± 0.06
Amyloid PET (Positive %)	41/147 (27.89%)	34/79 (43.04%)	7/7 (100%)	38/136 (27.94%)	34/74 (45.95%)	7/7 (100%)	288/330 (87.27%)	272/311 (87.45%)

*ADNI* Alzheimer’s Disease Neuroimaging Initiative, *A4* Anti-Amyloid Treatment in Asymptomatic Alzheimer’s Disease, *CN* Cognitively Normal, *MCI* Mild Cognitive Impairment, *AD* Alzheimer’s disease, *N* number of participants; *y* years, *SD* standard deviation, MMSE Mini mental state examination, *PET* positron emission tomography, *SUVR* standard uptake value ratio.

### PET imaging and processing

The baseline preprocessed [18]F-flortaucipir PET (tau PET) images of both A4 and ADNI studies were downloaded from the ADNI database (https://ida.loni.usc.edu/). The detailed acquisition procedures were previously reported^6,45,69^. In the ADNI cohort, we downloaded pre-processed PET images that were already co-registered, averaged, standardized, and had a voxel size and uniform resolution of 8 mm Full-Width at Half-Maximum (FWHM). For the A4 cohort, images with average of dynamic frames were downloaded.

T1-weighted magnetic resonance images (MRIs) that were acquired closest to the date of the baseline tau PET image scans and the respective tau PET images from the same participants were normalized to the standard native space using the SPM12 toolbox implemented in MATLAB (R2022a, Mathworks, Natick, MA, USA). Since ADNI PET images were downloaded with a uniform resolution of 8 mm FWHM, A4 cohort were smoothed to a resolution of 8 mm FWHM to ensure uniformity. The MRI images were processed using FreeSurfer, version 7.3.2 (https://surfer.nmr.mgh.harvard.edu/) to create anatomical regions of interest (ROIs) within a native space. Then, the tau PET images were co-registered with the respective FreeSurfer processed MRI data, and the SUVRs were calculated from the composite regions normalized to the cerebellar gray matter region, as described in the ADNI methods (https://adni.bitbucket.io/reference/docs/UCBERKELEYAV1451/UCBERKELEY_AV1451_Methods_2021-01-14.pdf). The composite regions include the entorhinal cortex, hippocampus, parahippocampal gyrus, fusiform gyrus, lingual gyrus, amygdala, lateral temporal regions, anterior and posterior cingulate, insula, temporal pole, frontal regions, lateral occipital regions, lateral parietal regions, bank of the superior temporal sulcus, pericalcarine, postcentral gyrus, cuneus, precentral gyrus, and paracentral lobule. All composite regions, including the cerebellar gray matter were derived from the Freesurfer Desikan-Killiany-Tourville cortical atlas.

Pre-processed amyloid-PET SUVR scores from the ADNI and A4 participants were accessed as described here^6^. Amyloid PET images were reconstructed, averaged, spatially aligned, interpolated, and smoothed. The mean standard uptake value ratios (SUVRs) were calculated using the whole gray matter cerebellum as the reference.

### SNP genotyping and imputation

The genotype data from A4, ADNI-1, ADNI-GO, ADNI-2, ADNI-3 and ADNI WGS cohorts were downloaded from the ADNI database (http://adni.loni.usc.edu). Genotyping methods for these cohorts are available at https://ida.loni.usc.edu/. Standard quality control (QC) procedures were performed separately for each genotype dataset. SNPs were excluded if there was a low genotype call rate <95%, Hardy–Weinberg equilibrium (HWE) test *P* value < 1.00 × 10^−6^ and the minor allele frequency (MAF) < 1%. Individuals with low genotype call rate <95%, cryptic relatedness (PI_HAT > 0.2) and sex discordance were removed. The number of SNPs and individuals included after quality control is shown in Supplementary Table 1. The genotype quality control for A4, ADNI-1, ADNI-GO, ADNI-2 and ADNI-WGS study cohorts are provided in our previous study^6^. ADNI-3 participants were genotyped using the Illumina Infinium Global Screening Array v2 platform. Before genotype QC, ADNI-3 included 327 participants and 759,993 SNPs. After QC, 322 participants and 503,036 SNPs remained.

After quality control, imputation was performed for all study datasets using the TOPMed reference panel implemented in the TOPMed imputation server. After imputation, SNPs with low quality (info score <0.9) and MAF < 0.01 were removed. The total number of SNPs remaining after imputation quality control in each study dataset is provided in Supplementary Table 1. We included individuals only if they had both genotype data and tau imaging data. This resulted in the inclusion of 733 individuals, which is shown in Table 1 and 7,725,775 SNPs for the association analysis. Of the 733 mutli-ethnic individuals, 95.8% were non-Hispanic whites, 3.5% were Hispanic, and the remaining 0.7% had unknown ethnicity. To address the population substructure within the dataset, we computed principal components (PCs) from the GWAS data using the “smartpca” script from the EIGENSOFT package. No PCs were significantly associated with tau-SUVRs, so we included the first 3PCs for our analysis.

### GWAS methods and statistical analysis

SNP-based GWAS was separately performed with Z-score standardized tau-SUVR measures adjusting for age, sex, and the first three principal components (PCs) in the A4 dataset and additionally AD diagnosis in the ADNI dataset. Because the A4 dataset consisted only of preclinical individuals, we treated it as a single group. In contrast, the ADNI dataset included CN, MCI, and AD participants. Therefore, clinical diagnosis was included as a covariate in the GWAS model for the ADNI dataset. Z-score normalization was applied to the tau-SUVR values in each cohort separately to harmonize the SUVR values across both cohorts.

A second model including *APOE*-ε4 dosage as a covariate was also tested. The analyses were conducted using PLINK software, version 1.9^70^. Results from A4 and ADNI studies were summarized in an inverse-weighted meta-analysis using METAL software^71^.

Rare variant analyses were conducted in tau-associated gene regions identified from the common variant GWAS using sequencing data from the Alzheimer’s Disease Sequencing Project (ADSP)^72^. Within each sub-cohort, linear regression models were applied adjusting for age, sex, and ancestry principal components, and results were subsequently meta-analyzed across cohorts.

Genome-wide gene-based analysis of tau-SUVR was implemented independently in A4 and ADNI using the MAGMA tool, version 1.09a^73^ with the ‘multi=all’ function, using the same covariates as described above. The ‘multi=all’ function aggregates the *P* value from three methods: *P*-value from linear regression for each gene, the mean of all SNP associations within each gene, and the sum of squared SNP Z statistics within each gene.

Results were summarized using the ‘meta’ function in MAGMA. Similarly, gene-based amyloid-SUVR analysis were conducted using summary statistics from a previous amyloid PET GWAS^6^.

Manhattan and quintile-quintile (Q-Q) plots for the SNP-based and gene-based GWAS results were generated using the ‘qqman’ package in the R program, version 4.3.1^74^. Pathways enrichment analyses were conducted in genes that were nominally significant with tau and amyloid SUVRs. Replication analysis was performed using summary statistics from a previously published tau-PET GWAS conducted by Ramanan et al. in 2020^13^, which included 754 individuals from the population-based Mayo Clinic Study of Aging.

### Rare-variant analysis

We selected genes of interest that showed significant associations in the common variant genome-wide analysis and conducted a rare variant meta-analysis within these regions. Variants with allele frequency less than 1% and minor allele count (MAC) ≥ 5 were included. Rare variant analyses were performed using data from the Alzheimer’s Disease Sequencing Project (ADSP)^75^, which comprises five sub-cohorts: A4, Alzheimer’s Disease Neuroimaging Initiative (ADNI), Health and Aging Brain Study-Health Disparities (HABS-HD), National Alzheimer’s Coordinating Center (NACC), and the Wisconsin Registry for Alzheimer’s Prevention (WRAP). Detailed sequencing procedures were provided elsewhere^75^.

Among these cohorts, 1561 individuals had available Tau-PET SUVR measures. Rare variants within the tau-associated gene regions of interest were included in the analysis. All variants were functionally annotated using SNPEff^76^ and ANNOVAR^77^. Regulatory annotation of SNPs was performed using publicly available functional genomics resources. Genomic context and regulatory features, including promoter and enhancer regions, were obtained from the ENCODE tracks^78^ downloaded from the UCSC Genome Browser (https://genome.ucsc.edu/). To capture brain-specific regulatory activity, we additionally queried the PsychENCODE database (https://www.psychencode.org/), which integrates epigenomic data from brain tissues^79^. SNPs were annotated based on overlaps with regulatory elements such as candidate cis-regulatory elements, chromatin accessibility peaks, and histone modification signals. Within each cohort, Tau-PET SUVR values were z-score normalized, and linear regression analyses were performed using PLINK v1.9^70^, adjusting for age, sex, and the first five principal components.

Cohort-specific association results were combined in a fixed-effect inverse-variance weighted meta-analysis using METAL^71^, provided there was no significant heterogeneity across cohorts (*P* > 0.05). For variants showing significant heterogeneity (p < 0.05), a random-effects meta-analysis was applied using GWAMA^80^. Additionally, we performed a rare-variant gene-based meta-analysis using variants from tau-associated gene regions, implemented in the skatMeta R package^81^.

In addition, tau-PET SUVR scores across all cohorts were harmonized using the ComBat R package, as PET scans varied by tracer: A4 and ADNI used [^18^F]-flortaucipir (https://ida.loni.usc.edu/), WRAP^82,83^ and NAAC^82^ used [^18^F]-MK-6240, and HABS-HD^84^ used [^18^F]-PI-2620. We then performed linear regression, adjusting for age, sex, and the first five principal components.

### Pleiotropy analysis

We evaluated whether tau-associated common variants exhibited pleiotropic effects with AD risk and amyloid levels using the “cfdr.pleio” R package (https://github.com/alexploner/cfdr.pleio). To evaluate AD risk, we used summary statistics from the tau-based meta-analysis together with results from the large-scale AD GWAS by Kunkle et al.^85^. Similarly, pleiotropic effects with amyloid were assessed by integrating tau-based meta-analysis results with summary statistics from our previously published large-scale amyloid GWAS.

### Mendelian randomization analysis

We applied the Mendelian Randomization (MR) framework to investigate whether genetically predicted circulating levels of plasma proteins are potentially causally linked with Tau PET levels using the GWAS summary data from A4 and ADNI.

### Study population and data sources (genetic associations of circulating plasma proteins)

Genetic associations of circulating plasma proteins were obtained from the UK Biobank Pharma Proteomics Project (UKBPPP) for individuals of European ancestry (*N* = 54,129; up to 95% Europeans)^86^. We limited our analyses to circulating plasma proteins whose cognate gene showed suggestive significance in our gene-based test (*P*_gene-based test_ < 0.05; *N* = 174) (Supplementary Table 15).

#### Instrument selection and MR analysis

To obtain genetic instrumental variables (IVs) for the 84 circulating plasma proteins^86^, we extracted *cis*-acting biallelic IVs located within ±1 Mb of the corresponding gene encoding the protein (defined as *cis*-pQTL [protein quantitative trait loci]). IVs were further filtered by the strength of association (*P* < 5 × 10^−8^), MAF > 0.01, and clumped at a pairwise linkage disequilibrium (LD) threshold of *r*^2^ < 0.001 in a window of 10000 KB using the TwoSampleMR R package to extract independent genetic variants^87^. IVs with F-statistics <10 was excluded to avoid weak instrument bias^88^. Data was harmonized using the “harmonized” function as implemented in the TwoSampleMR package. We utilized a randomly selected 10,000 European participants from the UKB for extracting IVs and the European reference panel of individuals from the 1000 genomes for performing clumping^89,90^. We considered Wald ratio (IV < 2), or inverse variance weighted (IVW) method (IV ≥ 2) as our primary methods, and IVW method was followed by weighted median and MR-Egger where possible. An FDR-corrected *P* < 0.05 was defined as statistically significant.

#### Co-localization analyses

To avoid confounding by LD in MR, we performed Bayesian genetic co-localization to identify shared causal variants between circulating plasma proteins and tau PET levels in the corresponding genomic region. The co-localization analysis was performed on the pre-defined *cis*-region (i.e., within ±500KB) of the corresponding coding gene. Priors were set as default that any SNP within the co-localization window was exclusively associated with the two traits with the probability of 1 × 10^−4^ and associated with both traits with the probability of 1 × 10^−5^ ^91^. A co-localization posterior probability (PP) higher than 70% [PPH4 > 70%] was considered strong evidence that the two traits are likely to co-localize in the region, while 50% < PPH4 < 70% was considered suggestive evidence. The co-localization analysis was conducted using the ‘coloc’ R package^91^.

### Polygenic risk score (PRS) analysis

The AD PGS was calculated using GWAS data from Kunkle et al.^85^, which included data from 94,437 participants. Relationships between AD PGS, tau, and amyloid SUVRs were analyzed in the A4 and ADNI cohorts, while controlling for age, sex, and the first 3 PCs, excluding the *APOE* region (500-kilobase pairs region upstream and downstream of the rs429358). Additionally, associations between tau and amyloid PGS with clinical and pathological AD were explored in the Religious Orders Study and the Memory and Aging Project (ROSMAP) cohort, along with related neuropathologies such as hippocampal sclerosis. PGS calculations utilized PRSice tool (v2)^92^ and PLINK (v1.9) software. For PGS estimation, SNPs were linkage disequilibrium (LD) clumped with *P* < 0.01 and *r²*>0.25. Detailed descriptions of the ROSMAP study cohort can be found elsewhere^93–95^, and demographic characteristics of the study participants are provided in Supplementary Table 2.

### Association analysis of brain cortical atrophy

The effect of tau-associated SNPs on brain cortical atrophy was evaluated through general linear models (GLM) adjusting for age and sex, in MATLAB (R2022a, The Mathworks, Natick, MA, USA) using the Surfstat toolbox (http://www.math.mcgill.ca/keith/surfstat/).

Mediation analyses were conducted to evaluate whether tau burden mediates the association between rs78636169 and cortical thickness across Braak stage regions using the R package ‘mediation’^96^. The SNP was coded additively, and covariates included age, sex, and principal components. The average causal mediation effect (ACME), representing the indirect effect of the SNP on cortical thickness through tau, and the average direct effect (ADE) were estimated using nonparametric bootstrap resampling with 5000 simulations. The total effect was defined as the sum of the indirects and direct effects. Analyses were performed separately for Braak stages 1–2, 3–4, and 5–6.

## Supplementary information

Supplementary Information

Supplementary Information

Supplementary Information

Supplementary Information

## Data Availability

The data analyzed in this study were obtained from existing publicly available databases. Alzheimer’s Disease Neuroimaging Initiative (ADNI) data are available at https://adni.loni.usc.edu/. Data from the Anti-Amyloid Treatment in Asymptomatic Alzheimer’s Disease (A4) Study are available through the Laboratory of Neuro Imaging (LONI) Image & Data Archive (IDA) (https://ida.loni.usc.edu/). Alzheimer’s Disease Sequencing Project (ADSP) data are available through the National Institute on Aging Genetics of Alzheimer’s Disease Data Storage Site (NIAGADS) (https://www.niagads.org/). Access to all datasets requires registration and approval in accordance with the respective data use agreements. No new data were generated for this study.
