# Supplementary material for: Common and rare variant analyses implicate JARID2 in cerebral tau deposition
Source: NPJ Dement. 2026 Jul 3;2(1):52. doi: 10.1038/s44400-026-00107-6 (PMC13331746; doi:10.1038/s44400-026-00107-6)
Supplement: Supplementary file 1 — Supplementary Information [file 44400_2026_107_MOESM1_ESM.pdf]

Common and Rare Variant Analyses Implicate *JARID2* in Cerebral Tau Deposition

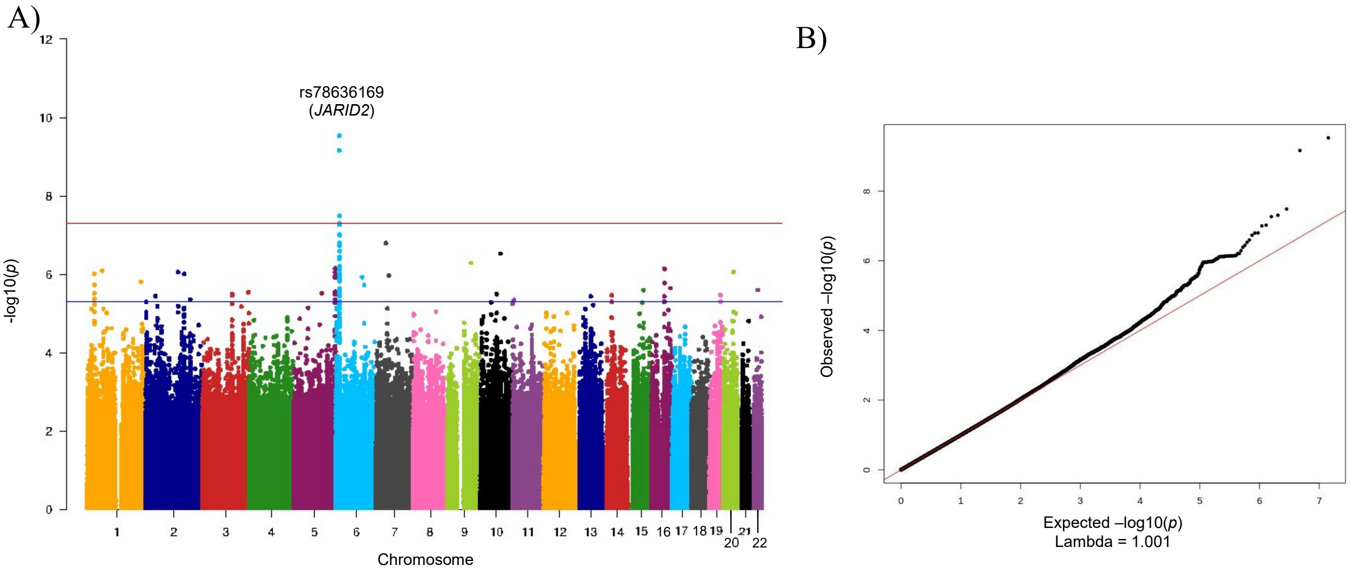

Supplementary Figure 1. SNPs associated with cerebral tau deposition in multi-ethnic subjects. (A) Manhattan plot showing meta-analysis *P*-values (depicted on the $-\log_{10}$ scale) from linear regression on cerebral tau deposition involving multi-ethnic subjects. The A4 cohort was adjusted for age, sex, and three principal components (PCs) for population substructure as covariates, and the ADNI cohort was adjusted for age, sex, diagnosis, and three PCs. The threshold for genome-wide significance is represented by a blue line at $P = 5\times10^{-8}$, while suggestive significance is indicated by a blue line at $P = 5\times10^{-6}$ threshold. **(B)** Quantile-Quantile (QQ) plots for the SNPs associated with cerebral tau deposition involving multi-ethnic subjects. The QQ plot showed no spurious genomic inflation (λ = 1.001).

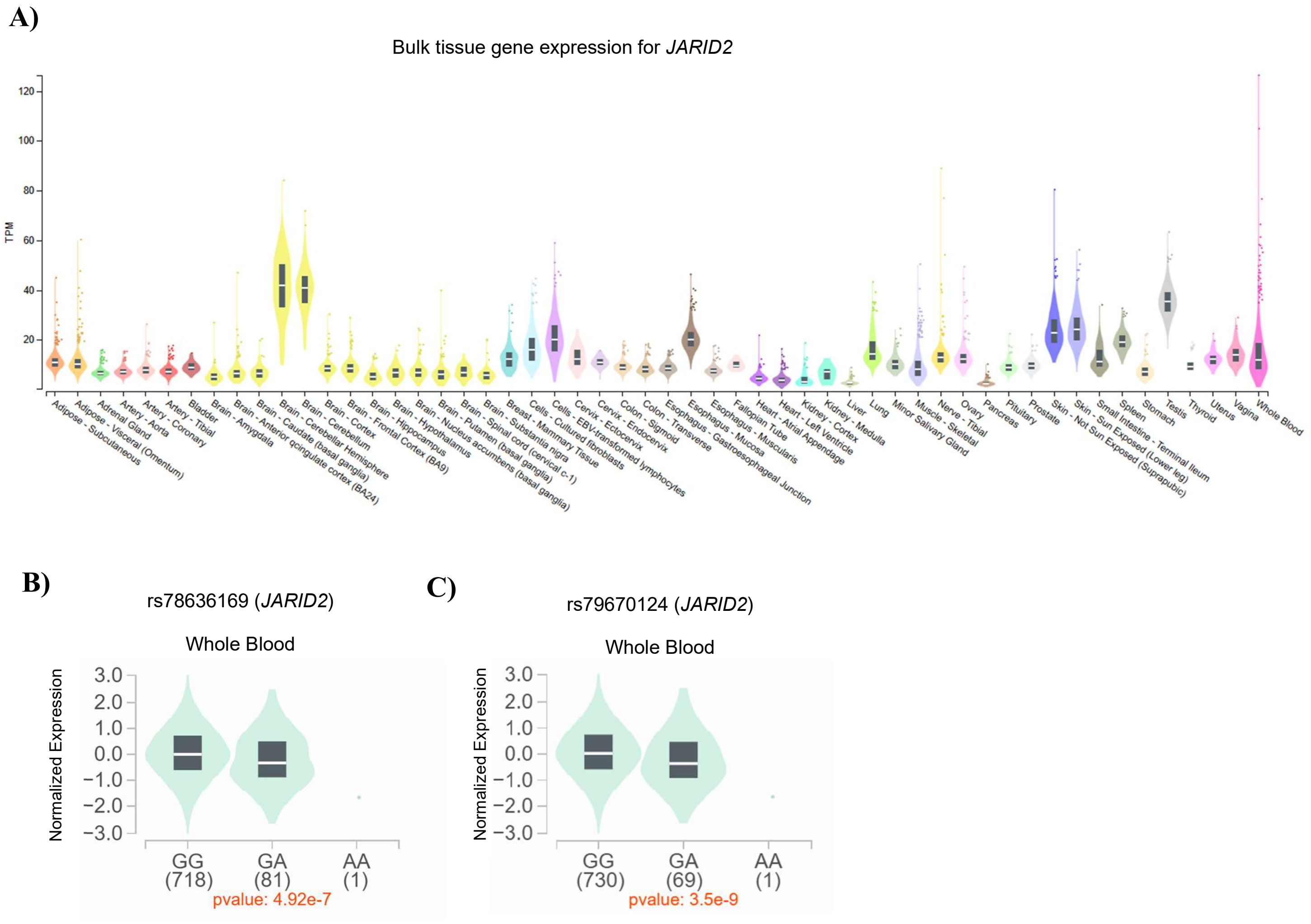

Supplementary Figure 2. Bulk tissue expression profile of *JARID2* across human tissues from GTEx.

A) Violin plots depict normalized transcript expression levels (TPM) of *JARID2* across diverse human tissues from the Genotype-Tissue Expression (GTEx) project. Each violin represents the distribution of expression values within a given tissue, with embedded boxplots indicating the median and interquartile range. *JARID2* shows relatively higher expression observed in select brain regions including cortex and cerebellum, and as well as in whole blood, supporting a potential role in brain-related biological processes. **B)** Top Tau GWAS hit SNP, rs78636169 in JARID2 showed reduced expression in whole blood, and **C)** rs7967012 in *JARID2* located in the active promoter region, which is also in strong linkage disequilibrium showed reduced expression in whole blood

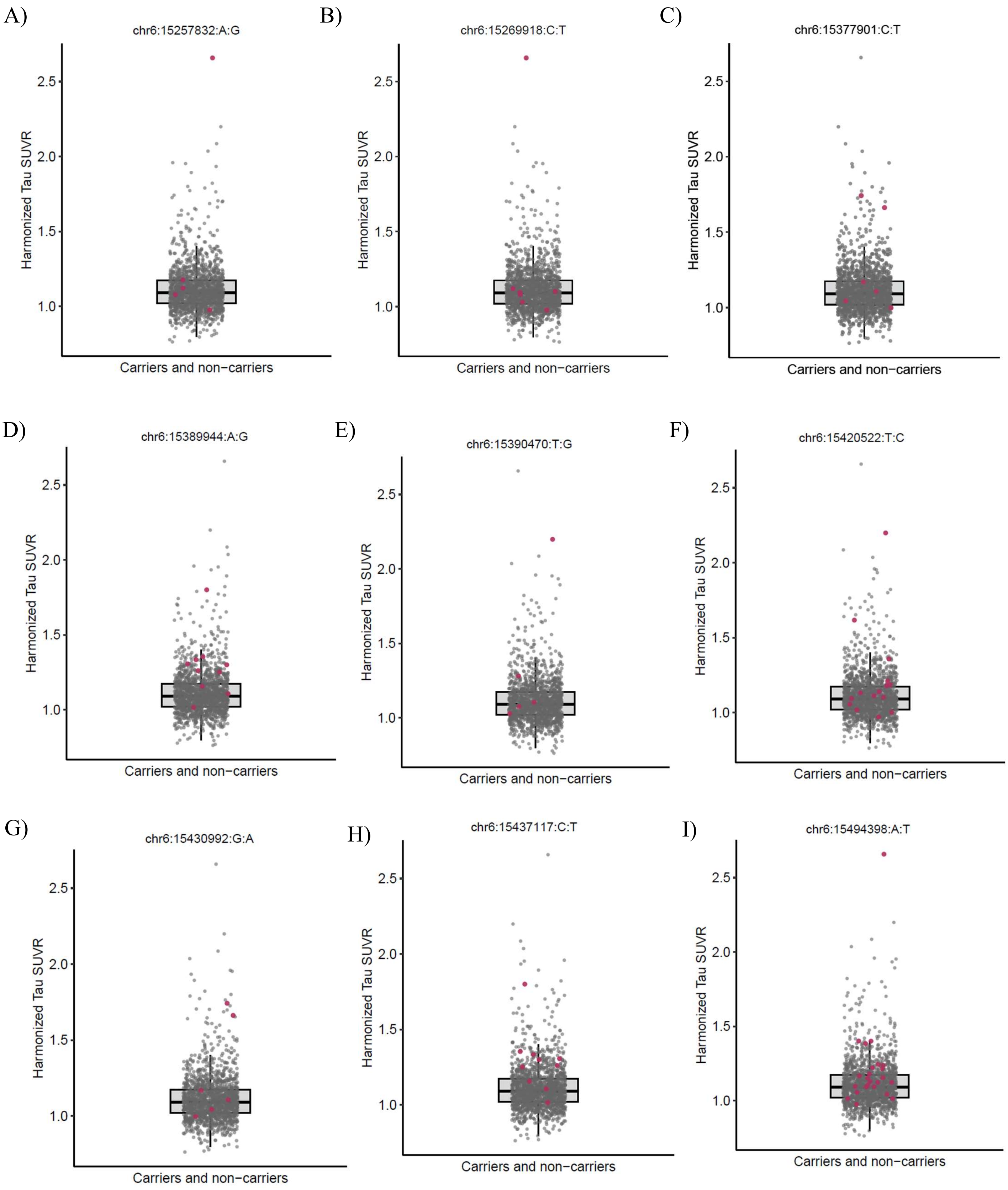
A)
chr6:15257832:A:G
B)
chr6:15269918:C:T
C)
chr6:15377901:C:T
D)
chr6:15389944:A:G
E)
chr6:15390470:T:G
F)
chr6:15420522:T:C
G)
chr6:15430992:G:A
H)
chr6:15437117:C:T
I)
chr6:15494398:A:T
Harmonized Tau SUVR
2.5
2.0
1.5
1.0
Carriers and non−carriers

J)

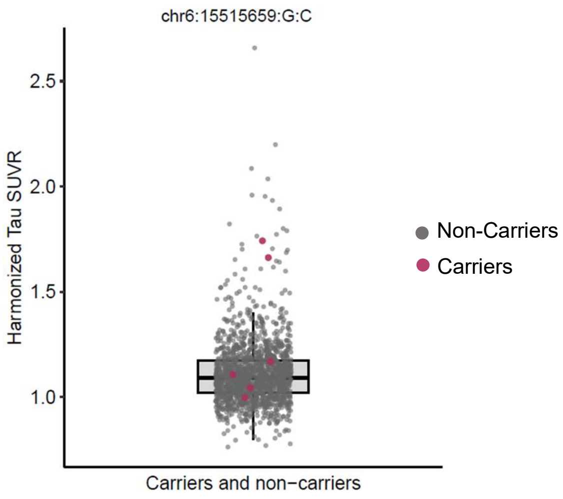

Supplementary Figure 3. Boxplots showing the distribution of harmonized tau SUVR levels in *JARID2* rare variants. **(A)** chr6:15257832:A:G, **(B)** chr6:15269918:C:T, **(C)** chr6:15377901:C:T, **(D)** chr6:15389944:A:G, **(E)** chr6:15390470:T:G, **(F)** chr6:15420522:T:C, **(G)** chr6:15430992:G:A, **(H)** chr6:15437117:C:T, **(I)** chr6:15494398:A:T, and **(J)** chr6:15515659:G:C variant carriers and non-carriers (x-axis) in the *JARID2* region showing harmonized tau SUVR distribution. Carriers are shown in maroon, and non-carriers are shown in gray.

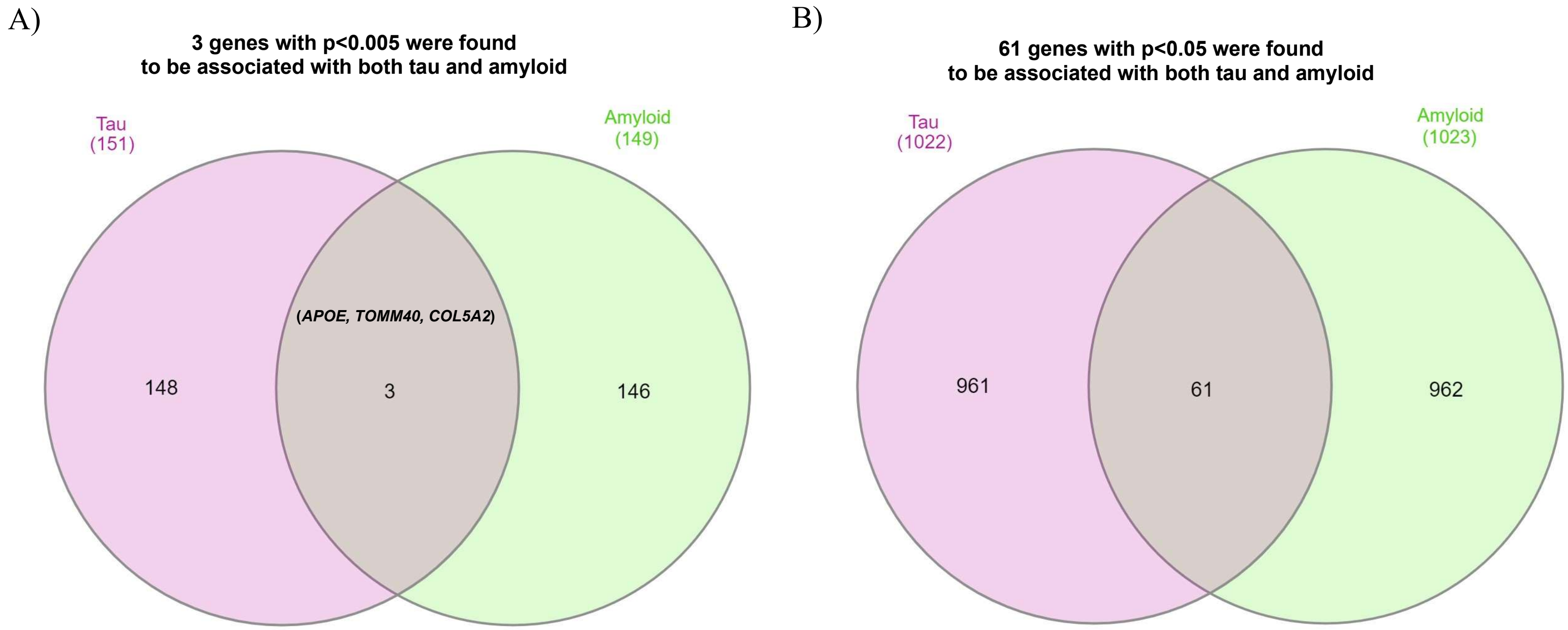

Supplementary Figure 4. Genes associated with amyloid and tau pathologies. **(A)** Genes that were strongly associated $p<0.005$ with tau and amyloid deposition were shown in figure **(A)** and the genes with nominal association were shown in figure **(B).**

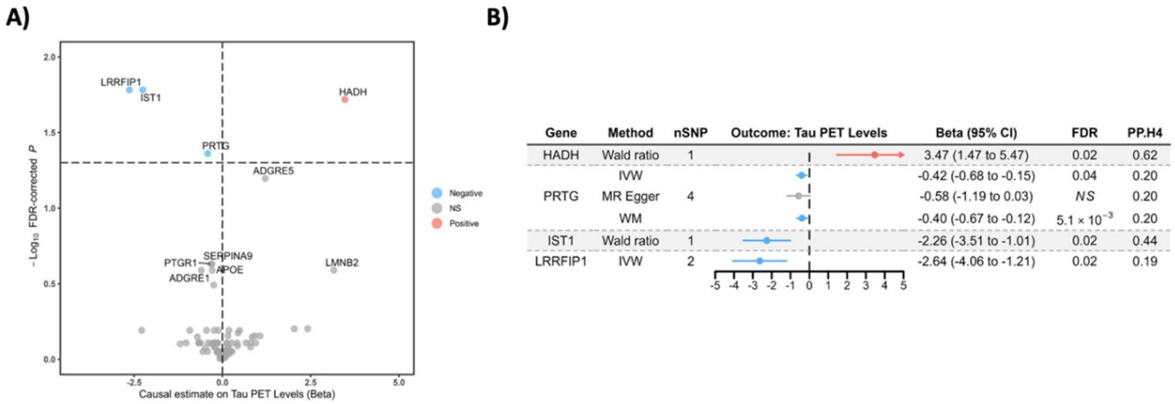

Supplementary Figure 5. Mendelian Randomization. MR effect of circulating plasma proteins on PET Tau levels using the UKBPPP proteomics data. **(A)** Volcano plot for the effect of 84 plasma proteins on PET Tau levels and **(B)** Forest plot showing the effect estimates for all proteins surpassing 5% FDR. FDR; false discovery rate, CI; confidence intervals, PP; posterior probability, SNP; single nucleotide polymorphism.

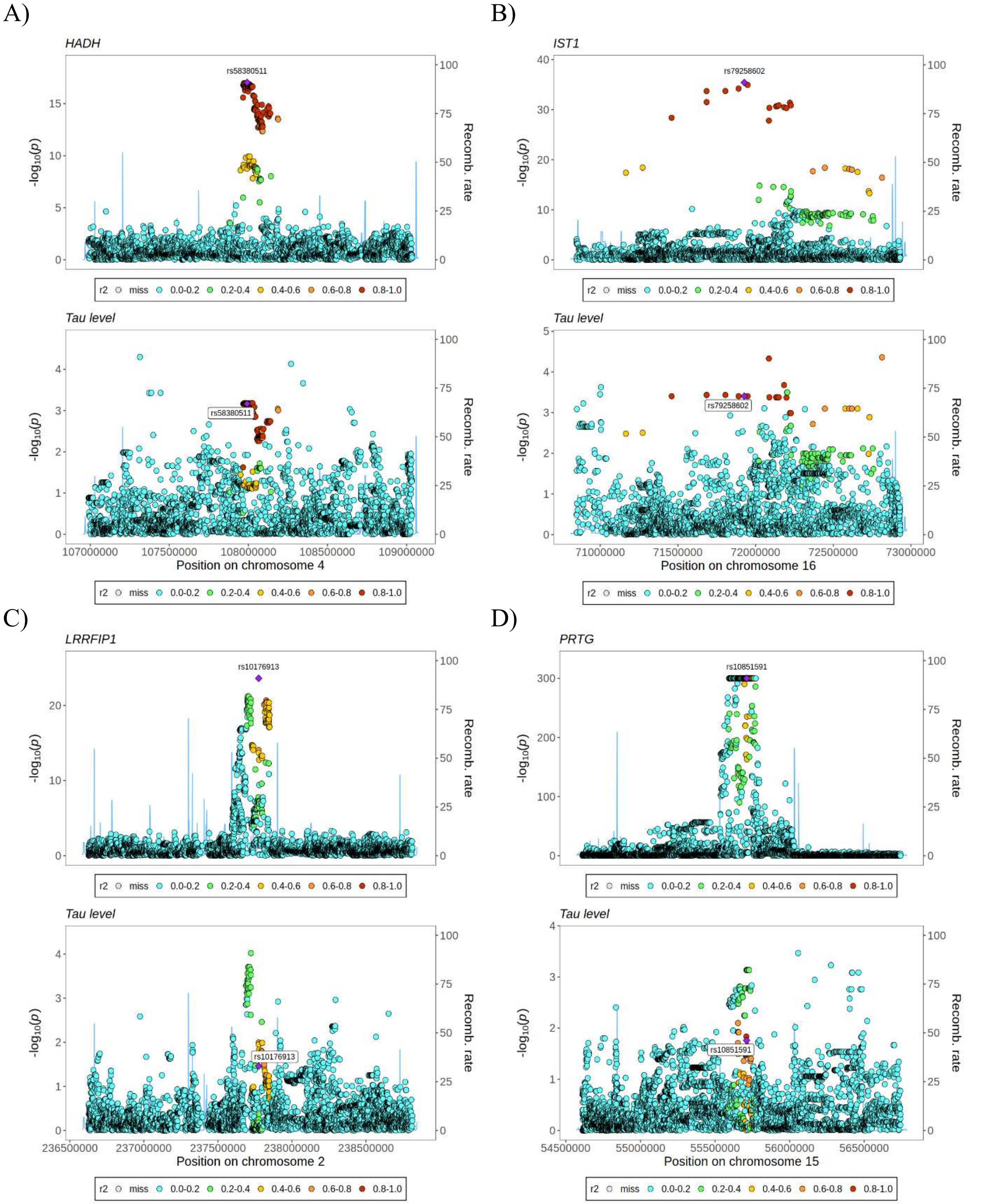

Supplementary Figure 6. Proteins co-localized with tau levels in Mendelian Randomization. Co-localization LocusZoom plots showing evidence of genetic co-localization for the **(A)** HADH **(B)** IST1 **(C)** LRRFIP1 and **(D)** PRTG that surpassed a 5% FDR threshold in the MR analysis.

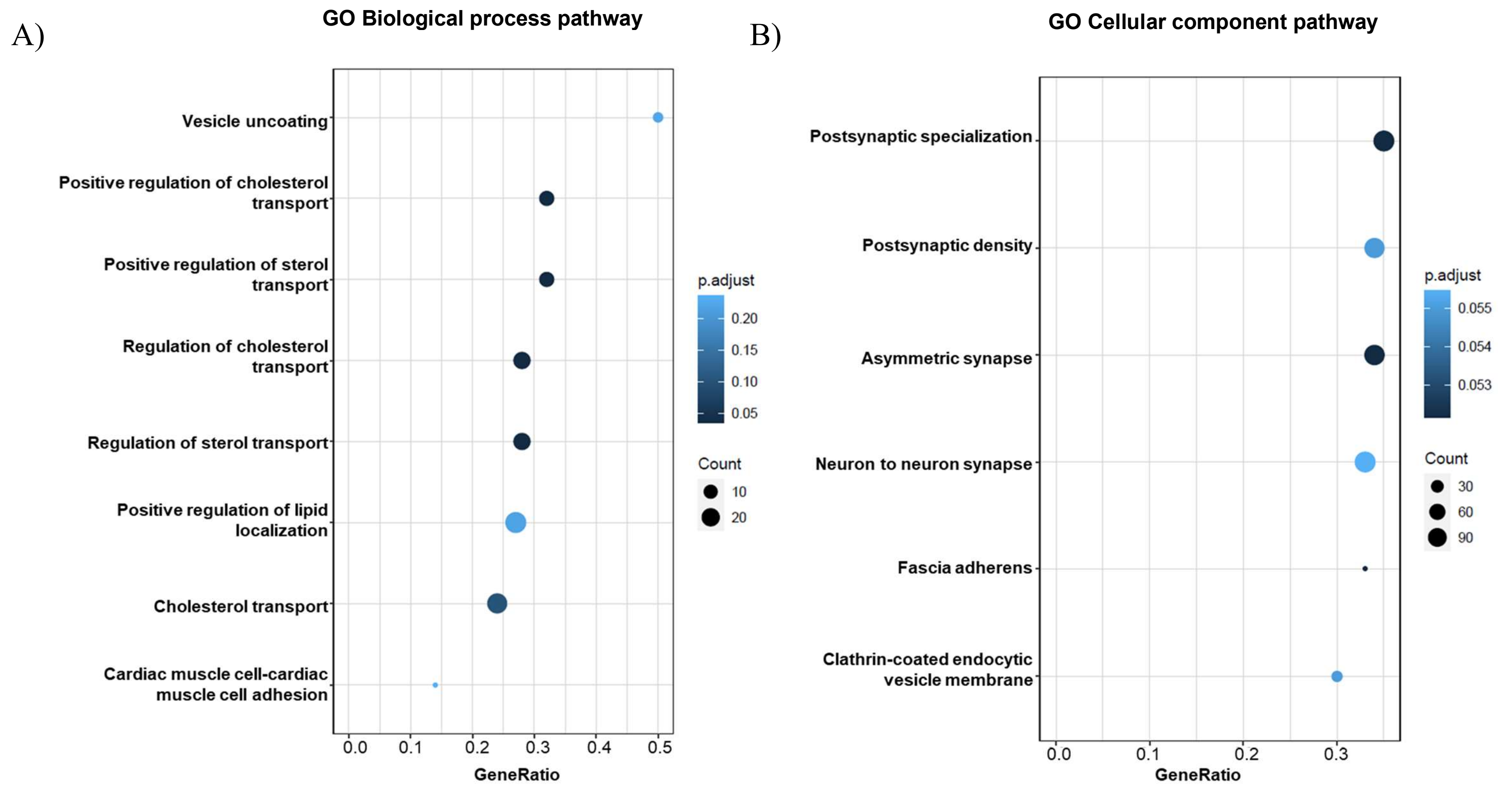

Supplementary Figure 7. Tau associated (*P*<0.05) genes enriched in Gene Ontology (GO) pathways. **(A)** GO Biological process pathway **(B)** GO Cellular component pathway.

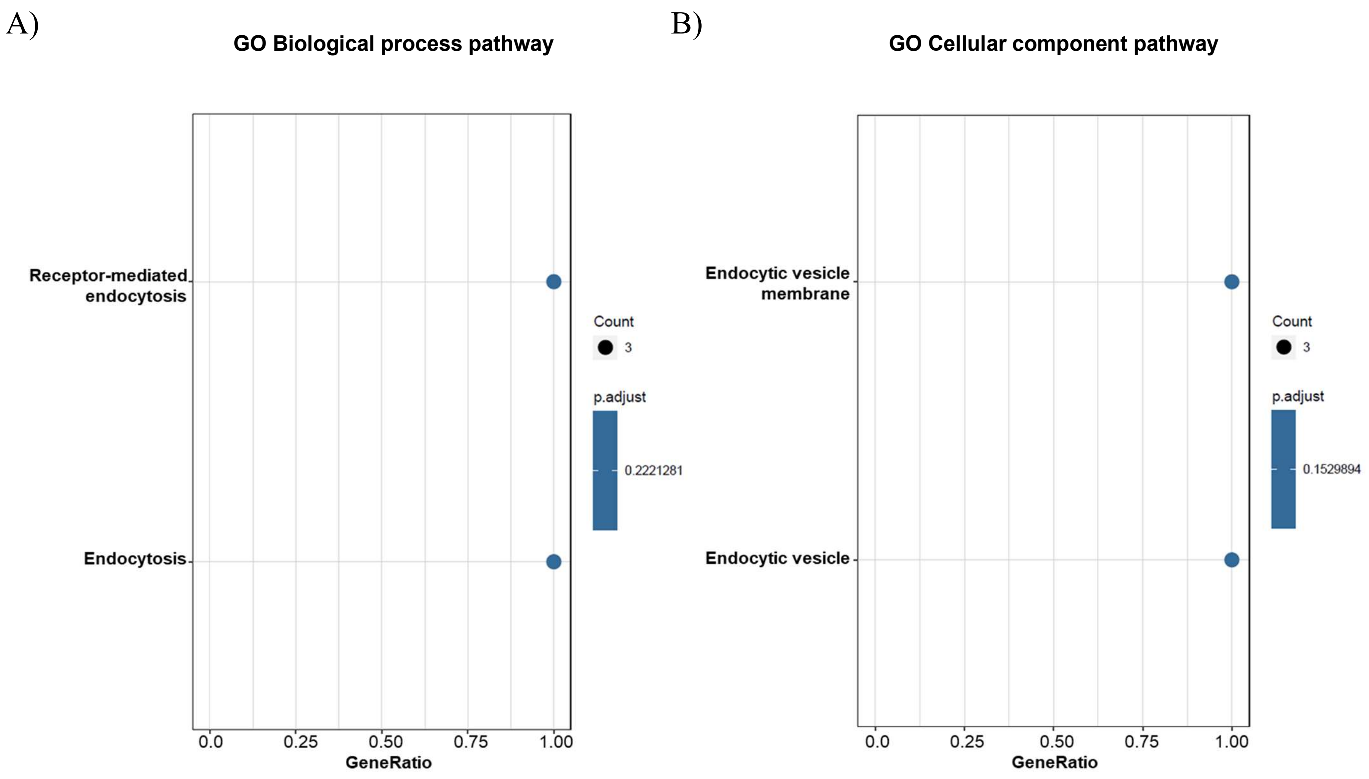

Supplementary Figure 8. Tau and Amyloid associated (*P*<0.05) genes enriched in Gene Ontology (GO) pathways. **(A)** GO Biological process pathway **(B)** GO Cellular component pathway.

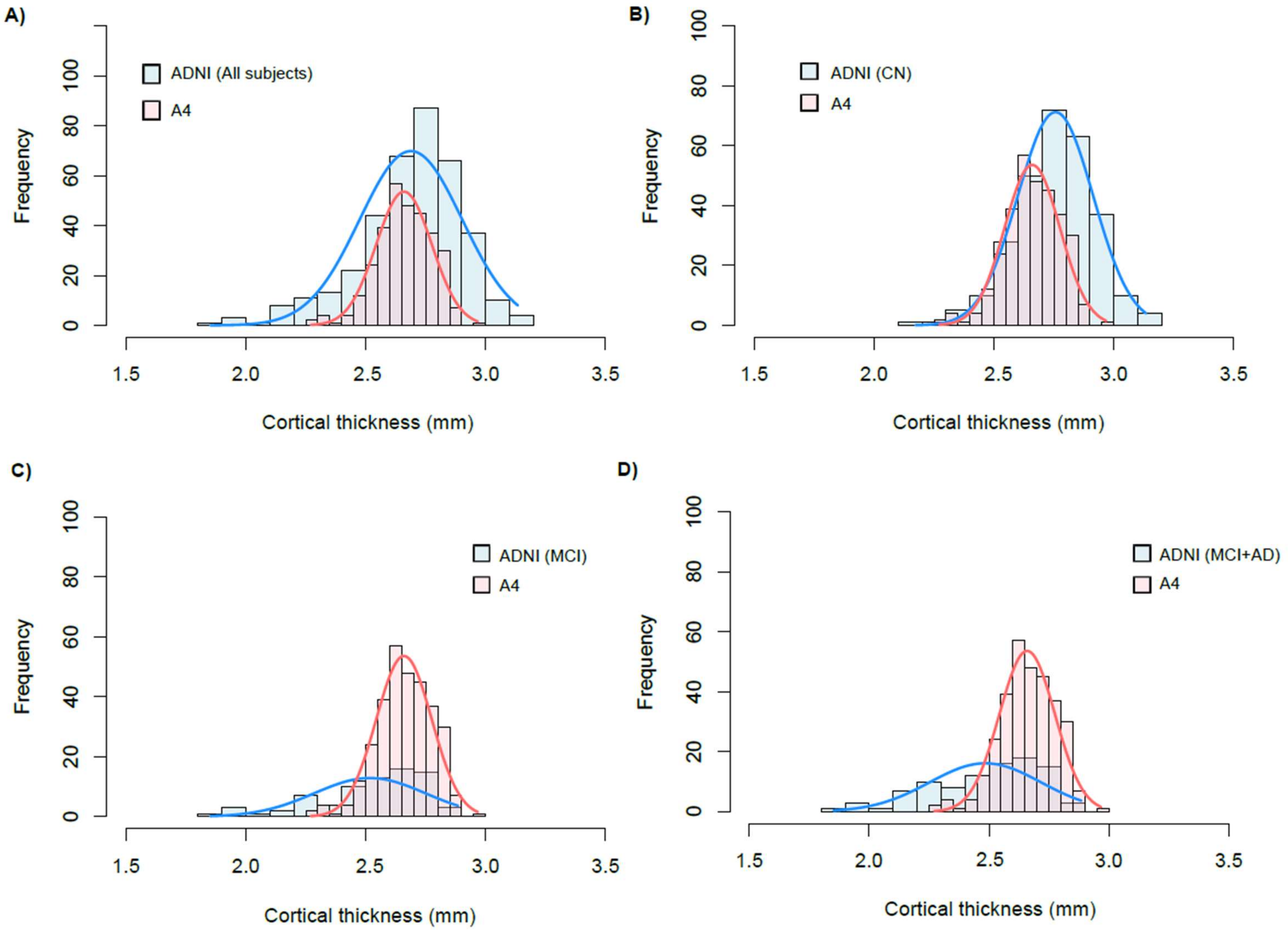

Supplementary Figure 9. Cortical thickness (mm) histograms for ADNI and A4 cohort individuals. A) All individuals from both cohorts. **B)** Cognitively normal (CN) individuals from ADNI and all individuals from A4. **C)** Mildly cognitively impaired (MCI) individuals from ADNI and all individuals from A4. **D)** MCI and Alzheimer's disease (AD) individuals from ADNI compared with all

individuals from A4. The solid line in the histograms denotes the estimated density curve. The red line indicates A4 cohort and the blue line indicates ADNI cohort.

Supplementary Table 1. Summary of each dataset after quality control and imputation

Study dataset	Genotype platform	Before QC		After QC		Post-imputation QC	
		No. of subjects	No. of SNPs	No. of subjects	No. of SNPs	No. of subjects	No. of SNPs
ADNI-1	Illumina 610-Quad	757	620,901	637	551,324	637	8,654,496
ADNI-GO/2	Illumina OmniExpress	432	730,525	429	644,362	429	7,117,117
ADNI-WGS	Illumina Omni 2.5M	812	2,379,855	807	1,530,732	807	8,855,414
ADNI-3	Illumina global screening array v2	327	759,993	322	503,036	322	8,372,458
A4	Illumina global screening array	3,502	700,078	3,452	489,637	3404	8,438,422

ADNI, Alzheimer's Disease Neuroimaging Initiative; A4, Anti-Amyloid Treatment in Asymptomatic Alzheimer's Disease; QC, quality control; No. of subjects, Number of subjects; No. of SNPs, Number of Single Nucleotide Polymorphisms

Supplementary Table 2. Demographic characteristic of ROSMAP cohort

**Sample groups**	**N**	**Age (y, mean ± SD)**	**Female (n, %)**	**Years of education (y, mean ± SD)**	**MMSE (mean ± SD)**	***APOE* ε4 (%)**
Cognitively normal	1094	81.04 ± 10.09	312 (28.52%)	16.25 ± 3.70	28.23 ± 1.70	188 (17.18%)
Mild cognitively impaired	562	87.16 ± 7.89	185 (32.92%)	16.04 ± 3.47	16.04 ± 3.47	118 (21.0%)
AD cases	849	89.63 ± 7.847	233 (27.44%)	16.03 ± 3.80	13.10 ± 8.67	297 (34.89%)
NIA-Reagan Diagnosis of AD cases	1589	89.70 ± 6.57	524 (32.97%)	16.24 ± 3.67	20.35 ± 9.48	398 (25.05%)
Hippocampal Sclerosis positive	129	81.98 ± 6.67	30 (23.26%)	15.75 ± 3.40	13.07 ± 10.26	2 (1.56%)
Hippocampal Sclerosis negative	1320	89.24 ± 6.52	450 (34.09%)	16.30 ± 3.66	21.18 ± 8.99	315 (23.86%)

AD, Alzheimer’s Disease; N, number of subjects; y Years; SD, Standard Deviation; MMSE, Mini Mental State Examination

Suuplementary Table 3. Association analysis involving non-Hispanic White subjects showing SNPs with genome-wide significant association for Tau deposition

SNP	Nearest gene	CHR	BP	Variant type	Overlapping regulatory region			Effect	Tau-SUVR Meta-analysis					
					Lymphoblastoid cell line	Embryonic stem cells	Adult human brain tissues		Beta	SE	*P* -value	Dir	HetISq	HetPVal
rs78636169	*JARID2*	6	15242005	Upstream variant	Heterochromatin; low signal	Weak transcribed	None	A	0.71	0.12	5.76E-10	++	0	0.9
rs200751686	*JARID2*	6	15213555	Intergenic region	Heterochromatin; low signal	Heterochromatin; low signal	None	CA	0.68	0.11	2.74E-09	++	0	0.86
rs7292124	*ISX*	22	35419122	Intergenic region	Heterochromatin; low signal	Heterochromatin; low signal	None	C	0.69	0.12	2.20E-08	++	0	0.46
rs116155001	*RP5*	1	107079904	Intergenic region	Heterochromatin; low signal	Heterochromatin; low signal	None	A	1.32	0.25	8.76E-08	++	0	0.65
rs114422826	*RP5*	1	107055366	Intergenic region	Heterochromatin; low signal	Heterochromatin; low signal	None	G	1.32	0.25	8.78E-08	++	0	0.65
rs112518363	*INTS10*	8	19661058	Intergenic region	Heterochromatin; low signal	Heterochromatin; low signal	None	G	1.15	0.22	1.05E-07	++	0	0.87
rs114940578	*AC004901.1*	7	47001136	Intron variant	Heterochromatin; low signal	Heterochromatin; low signal	None	A	1.15	0.22	1.76E-07	++	16.9	0.27
rs116560676	*AC004901.1*	7	47001137	Intron variant	Heterochromatin; low signal	Heterochromatin; low signal	None	T	1.15	0.22	1.76E-07	++	16.9	0.27
rs73102974	*AC004901.1*	7	46999060	Intron variant	Heterochromatin; low signal	Heterochromatin; low signal	None	G	1.15	0.22	1.76E-07	++	16.9	0.27
rs80257052	*JARID2*	6	15366460	Intron variant	Strong_Enhancer	Transcriptional transition	None	T	0.66	0.13	1.77E-07	++	0	0.95
rs79991339	*JARID2*	6	15407383	Intron variant	Weak transcribed	Transcriptional transition	None	C	0.63	0.12	2.51E-07	++	0	0.97
rs74671815	*RP11-146I2.1*	6	15094935	Upstream gene variant	Heterochromatin; low signal	Heterochromatin; low signal	None	A	0.67	0.13	2.75E-07	++	0	0.83
rs139965611	*AC005022.1*	7	41642607	Intergenic region	Heterochromatin; low signal	Heterochromatin; low signal	None	T	0.77	0.15	2.79E-07	++	0	0.43
rs7262931	*SGK2*	20	42188050	Intron variant	Heterochromatin; low signal	Weak transcribed	None	C	0.51	0.1	2.89E-07	++	0	0.64
rs56176153	*Y_RNA*	2	138561026	Intergenic region	Heterochromatin; low signal	Heterochromatin; low signal	None	T	0.37	0.07	3.29E-07	++	0	0.68
rs112441779	*RN7SL332P*	6	15131245	Intergenic region	Weak transcribed	Heterochromatin; low signal	None	T	0.64	0.13	3.38E-07	++	0	0.84
rs114272033	*ISCA1P2*	1	229192813	Intergenic region	Polycomb-repressed	Heterochromatin; low signal	None	A	1.01	0.2	3.92E-07	++	0	0.7
rs75305046	*JARID2*	6	15335703	Intron variant	Strong_Enhancer	Transcriptional transition	None	C	0.63	0.12	4.86E-07	++	0	0.91
rs10888752	*ZYG11B*	1	53193046	Intron variant	Weak_Promoter	Weak_Promoter	None	G	1.1	0.22	4.96E-07	++	35.9	0.21
rs138727281	*NRG3*	10	84674214	Intron variant	Weak transcribed	Heterochromatin; low signal	None	A	1.0207	0.2039	5.52E-07	++	0	0.5868
rs113253465	*LSAMP*	3	116995385	Intron variant	Heterochromatin; low signal	Heterochromatin; low signal	None	A	1.2037	0.2411	5.97E-07	++	0	0.891
rs118149470	*GABBR2*	9	101428700	Intron variant	Heterochromatin; low signal	Heterochromatin; low signal	None	A	1.1779	0.2365	6.36E-07	++	0	0.6511
rs12529719	*AL050335.1*	6	15228489	Intergenic region	Heterochromatin; low signal	Heterochromatin; low signal	None	A	0.6175	0.1245	7.03E-07	++	0	0.7468
rs543712182	*AL050335.1*	6	15232756	Intergenic region	Heterochromatin; low signal	Heterochromatin; low signal	None	A	0.6072	0.123	7.90E-07	++	0	0.8078
rs75049360	*JARID2*	6	15265899	Intron variant	Strong_Enhancer	Weak_Promoter	Distal Enhancer	T	0.6634	0.1347	8.47E-07	++	0	0.8587
rs142629644	*MIR3144*	6	120551246	Intergenic region	Heterochromatin; low signal	Insulator	None	G	1.1353	0.2311	8.98E-07	++	0	0.5112
rs61768462	*RP3-357I16.1*	1	30727316	Intergenic region	Heterochromatin; low signal	Weak_Enhancer	None	T	0.7544	0.1541	9.86E-07	++	0	0.4191
rs35330226	*RP3-357I16.1*	1	30713744	Intergenic region	Heterochromatin; low signal	Heterochromatin; low signal	None	T	0.6479	0.1324	9.91E-07	++	4.1	0.3071
rs4949560	*RP3-357I16.1*	1	30714577	Intergenic region	Heterochromatin; low signal	Heterochromatin; low signal	None	T	0.6479	0.1324	9.91E-07	++	4.1	0.3071
rs7525079	*RP3-357I16.1*	1	30716585	Intergenic region	Heterochromatin; low signal	Heterochromatin; low signal	None	T	0.6479	0.1324	9.91E-07	++	4.1	0.3071
rs6661218	*RP3-357I16.1*	1	30711813	Intergenic region	Heterochromatin; low signal	Heterochromatin; low signal	None	C	0.6479	0.1324	9.92E-07	++	4.1	0.3072
rs7529543	*RP3-357I16.1*	1	30716625	Intergenic region	Heterochromatin; low signal	Heterochromatin; low signal	None	G	0.6479	0.1324	9.92E-07	++	4.1	0.3072
rs114742337	*RP3-357I16.1*	1	63383903	Intergenic region	Heterochromatin; low signal	Weak_Enhancer	None	A	0.6483	0.1326	1.01E-06	++	0	0.583
rs138887421	*AC004870.4*	7	47008460	Upstream gene variant	Heterochromatin; low signal	Heterochromatin; low signal	None	A	1.098	0.2253	1.10E-06	++	62.8	0.1013
rs56106339	*LMNA*	1	156078267	Intron variant	Weak transcribed	Weak_Enhancer	None	C	0.8032	0.1654	1.20E-06	++	55.4	0.1342
rs34326651	*JARID2*	6	15374428	synonymous variant	Transcriptional elongation	Transcriptional transition	None	C	0.5638	0.1162	1.23E-06	++	0	0.7474
rs139265905	*RP11-662J14.2*	14	41183763	Intergenic region	Heterochromatin; low signal	Heterochromatin; low signal	None	C	0.8868	0.1829	1.24E-06	++	0	0.4201
rs62038344	*AC024590.1*	16	82390090	Intergenic region	Heterochromatin; low signal	Heterochromatin; low signal	None	A	0.5509	0.1137	1.26E-06	++	0	0.5952
rs140760383	*CTC-786C10.1*	16	85307121	Intergenic region	Heterochromatin; low signal	Weak transcribed	None	G	0.6104	0.1267	1.45E-06	++	72	0.05879

rs111646565	*JARID2*	6	15251149	Upstream gene variant	Weak_Promoter	Weak_Enhancer	None	A	0.5967	0.1239	1.46E-06	++	0	0.8674
rs12527312	*AL050335.1*	6	15230136	Intergenic region	Heterochromatin; low signal	Heterochromatin; low signal	None	T	0.5967	0.1239	1.46E-06	++	0	0.8674
rs12530231	*AL050335.1*	6	15229606	Intergenic region	Heterochromatin; low signal	Heterochromatin; low signal	None	A	0.5967	0.1239	1.46E-06	++	0	0.8674
rs73359689	*JARID2*	6	15254908	Intron variant	Strong_Enhancer	Transcriptional transition	Distal Enhancer	A	0.5967	0.1239	1.46E-06	++	0	0.8674
rs75764096	*JARID2*	6	15250495	Upstream gene variant	Active_Promoter	Transcriptional transition	None	A	0.5967	0.1239	1.46E-06	++	0	0.8674
rs77428746	*AL050335.1*	6	15237771	Intergenic region	Heterochromatin; low signal	Heterochromatin; low signal	None	A	0.5967	0.1239	1.46E-06	++	0	0.8674
rs79670124	*JARID2*	6	15246022	Upstream gene variant	Active_Promoter	Active_Promoter	Proximal Enhancer	A	0.5967	0.1239	1.46E-06	++	0	0.8674
rs79851650	*AL050335.1*	6	15233151	Intergenic region	Weak_Enhancer	Heterochromatin; low signal	None	A	0.5967	0.1239	1.46E-06	++	0	0.8674
6:15230238	*AL050335.1*	6	15230238	Intergenic region	Heterochromatin; low signal	Heterochromatin; low signal	None	T	0.5967	0.1239	1.47E-06	++	0	0.8675
6:15252987	*JARID2*	6	15252987	Upstream gene variant	Weak_Promoter	Transcriptional transition	None	GT	0.5967	0.1239	1.47E-06	++	0	0.8675
rs111483604	*AL050335.1*	6	15228997	Intergenic region	Heterochromatin; low signal	Heterochromatin; low signal	None	G	0.5967	0.1239	1.47E-06	++	0	0.8675
rs12527270	*AL050335.1*	6	15229776	Intergenic region	Heterochromatin; low signal	Heterochromatin; low signal	None	G	0.5967	0.1239	1.47E-06	++	0	0.8675
rs12529010	*AL050335.1*	6	15229811	Intergenic region	Heterochromatin; low signal	Heterochromatin; low signal	None	G	0.5967	0.1239	1.47E-06	++	0	0.8675
rs6904999	*AL050335.1*	6	15232306	Intergenic region	Heterochromatin; low signal	Heterochromatin; low signal	None	G	0.5967	0.1239	1.47E-06	++	0	0.8675
rs11800251	*RP3-357I16.1*	1	30724705	Intergenic region	Heterochromatin; low signal	Heterochromatin; low signal	None	T	0.748	0.1558	1.59E-06	++	0	0.3762
rs151003871	*RP3-357I16.1*	1	30726244	Intergenic region	Heterochromatin; low signal	Heterochromatin; low signal	None	A	0.748	0.1558	1.59E-06	++	0	0.3762
rs61766339	*RP3-357I16.1*	1	30712987	Intergenic region	Heterochromatin; low signal	Heterochromatin; low signal	None	A	0.748	0.1558	1.59E-06	++	0	0.3762
rs61766359	*RP3-357I16.1*	1	30722033	Intergenic region	Heterochromatin; low signal	Heterochromatin; low signal	None	A	0.748	0.1558	1.59E-06	++	0	0.3762
rs6685389	*RP3-357I16.1*	1	30709585	Intergenic region	Heterochromatin; low signal	Heterochromatin; low signal	None	A	0.748	0.1558	1.59E-06	++	0	0.3762
rs76054017	*RP3-357I16.1*	1	30721971	Intergenic region	Heterochromatin; low signal	Heterochromatin; low signal	None	A	0.748	0.1558	1.59E-06	++	0	0.3762
rs61766338	*RP3-357I16.1*	1	30710379	Intergenic region	Heterochromatin; low signal	Heterochromatin; low signal	None	C	0.748	0.1559	1.59E-06	++	0	0.3762
rs61768463	*RP3-357I16.1*	1	30727501	Intergenic region	Heterochromatin; low signal	Weak_Enhancer	None	G	0.748	0.1559	1.59E-06	++	0	0.3762
rs6702811	*RP3-357I16.1*	1	30709634	Intergenic region	Heterochromatin; low signal	Heterochromatin; low signal	None	C	0.748	0.1559	1.59E-06	++	0	0.3762
rs138338441	*AC092684.1*	2	164969273	Intron variant	Heterochromatin; low signal	Heterochromatin; low signal	None	T	1.0849	0.2262	1.62E-06	++	0	0.5345
rs61968319	*NALCN*	13	101581782	Intron variant	Heterochromatin; low signal	Heterochromatin; low signal	None	T	1.1597	0.2426	1.75E-06	++	0	0.8404
rs115191082	*FTLP17*	1	104640701	Intergenic region	Heterochromatin; low signal	Heterochromatin; low signal	None	T	1.0604	0.2219	1.77E-06	++	24.5	0.2497
rs112772777	*CES5A*	16	56079580	Intergenic region	Heterochromatin; low signal	Heterochromatin; low signal	None	A	0.6143	0.1288	1.85E-06	++	0	0.4601
rs78641048	*CES5A*	16	56080808	Intergenic region	Heterochromatin; low signal	Heterochromatin; low signal	None	C	0.6143	0.1288	1.85E-06	++	0	0.4601
rs150477486	*FYN*	6	112024290	Upstream gene variant	Transcriptional elongation	Heterochromatin; low signal	None	T	0.9363	0.1972	2.06E-06	++	52.3	0.1476
rs150964852	*PALM2*	9	112452018	Intron variant	Heterochromatin; low signal	Heterochromatin; low signal	None	T	0.9481	0.2009	2.38E-06	++	20.3	0.2625

SNP, Single Nucleotide Polymorphisms; Chr, Chromosome; A1, Effect allele; MAF, Minor Allele Frequency; SE, Standard error; Dir, Effect direction; Het ChiSq, Chi-square value for heterogeneity test; Het PVal, *P*-value for heterogeneity in effect sizes in meta-analysis.

Suuplementary Table 4. Association analysis involving multi-ethnic subjects showing SNPs with genome-wide significant association for Tau deposition

SNP	Gene	CHR	BP	Variant type	Overlapping regulatory region			Effect	Tau-SUVR meta-analysis					
					Lymphoblastoid cell line	Embryonic stem cells	Adult human brain tissues		Beta	SE	*P* -value	Dir	HetISq	HetPVal
rs78636169	*JARID2*	6	15242005	Upstream gene variant	Heterochromatin; low signal	Weak transcribed	None	A	0.7	0.11	2.96E-10	++	0	0.84
rs200751686	*AL050335.1*	6	15213555	Intergenic region	Heterochromatin; low signal	Heterochromatin; low signal	None	CA	0.67	0.11	6.84E-10	++	0	0.74
rs80257052	*JARID2*	6	15366460	Intron variant	Strong_Enhancer	Transcriptional transition	None	T	0.68	0.12	3.27E-08	++	0	0.95
rs79991339	*JARID2*	6	15407383	Intron variant	Weak transcribed	Transcriptional transition	None	C	0.65	0.12	4.93E-08	++	0	0.97
rs74671815	*RP11*	6	15094935	Upstream gene variant	Heterochromatin; low signal	Heterochromatin; low signal	None	A	0.69	0.13	5.42E-08	++	0	0.83
rs75305046	*JARID2*	6	15335703	Intron variant	Strong_Enhancer	Transcriptional transition	None	C	0.65	0.12	9.51E-08	++	0	0.92
rs17576261	*JARID2*	6	15382780	Intron variant	Weak_Enhancer	Transcriptional transition	None	A	0.62	0.12	9.99E-08	++	0	0.7
rs139965611	*AC005022.1*	7	41642607	Intergenic region	Heterochromatin; low signal	Heterochromatin; low signal	None	T	0.78	0.15	1.59E-07	++	0	0.34
rs75049360	*JARID2*	6	15265899	Intron variant	Strong_Enhancer	Weak_Promoter	Distal Enhancer	T	0.69	0.13	1.60E-07	++	0	0.87
rs112441779	*RN7SL332P*	6	15131245	Intergenic region	Weak transcribed	Heterochromatin; low signal	None	T	0.64	0.12	1.84E-07	++	0	0.65
rs34326651	*JARID2*	6	15374428	synonymous variant	Transcriptional elongation	Transcriptional transition	None	C	0.59	0.11	2.53E-07	++	0	0.73
rs138727281	*NRG3*	10	84674214	Intron variant	Weak transcribed	Heterochromatin; low signal	None	A	1.05	0.2	2.97E-07	++	0	0.53
rs12529719	*AL050335.1*	6	15228489	Intergenic region	Heterochromatin; low signal	Heterochromatin; low signal	None	A	0.6	0.12	3.62E-07	++	0	0.49
rs543712182	*AL050335.1*	6	15232756	Intergenic region	Heterochromatin; low signal	Heterochromatin; low signal	None	A	0.6	0.12	4.08E-07	++	0	0.55
rs118149470	*GABBR2*	9	101428700	Intron variant	Heterochromatin; low signal	Heterochromatin; low signal	None	A	1.1911	0.2372	5.13E-07	++	0	0.6058
rs76744906	*JARID2*	6	15461236	Intron variant	Transcriptional elongation	Transcriptional transition	None	G	0.5738	0.1151	6.23E-07	++	0	0.618
rs78795505	*JARID2*	6	15474235	Intron variant	Transcriptional elongation	Transcriptional transition	None	G	0.5738	0.1151	6.23E-07	++	0	0.618
rs4608937	*RP11*	5	177510571	Intergenic region	Weak transcribed	Weak_Enhancer	None	A	0.2647	0.0534	7.01E-07	++	67.2	0.08065
rs112772777	*CES5A*	16	56079580	Intergenic region	Heterochromatin; low signal	Heterochromatin; low signal	None	A	0.6334	0.1278	7.20E-07	++	0	0.3447
rs78641048	*CES5A*	16	56080808	Intergenic region	Heterochromatin; low signal	Heterochromatin; low signal	None	C	0.6335	0.1278	7.22E-07	++	0	0.3449
rs111646565	*JARID2*	6	15251149	Upstream gene variant	Weak_Promoter	Weak_Enhancer	None	A	0.5848	0.118	7.26E-07	++	0	0.5887
rs12527312	*AL050335.1*	6	15230136	Intergenic region	Heterochromatin; low signal	Heterochromatin; low signal	None	T	0.5848	0.118	7.26E-07	++	0	0.5887
rs75764096	*JARID2*	6	15250495	Upstream gene variant	Active_Promoter	Transcriptional transition	None	A	0.5848	0.118	7.26E-07	++	0	0.5887
6:15230238	*AL050335.1*	6	15230238	Intergenic region	Heterochromatin; low signal	Heterochromatin; low signal	None	T	0.5848	0.1181	7.35E-07	++	0	0.5888
rs111483604	*AL050335.1*	6	15228997	Intergenic region	Heterochromatin; low signal	Heterochromatin; low signal	None	G	0.5848	0.1181	7.35E-07	++	0	0.5888
rs12527270	*AL050335.1*	6	15229776	Intergenic region	Heterochromatin; low signal	Heterochromatin; low signal	None	G	0.5848	0.1181	7.35E-07	++	0	0.5888
rs12529010	*AL050335.1*	6	15229811	Intergenic region	Heterochromatin; low signal	Heterochromatin; low signal	None	G	0.5848	0.1181	7.35E-07	++	0	0.5888
rs12530231	*AL050335.1*	6	15229606	Intergenic region	Heterochromatin; low signal	Heterochromatin; low signal	None	A	0.5872	0.1187	7.60E-07	++	0	0.6014
rs73359689	*JARID2*	6	15254908	Intron variant	Strong_Enhancer	Transcriptional transition	Distal Enhancer	A	0.5872	0.1187	7.60E-07	++	0	0.6014
rs77428746	*AL050335.1*	6	15237771	Intergenic region	Heterochromatin; low signal	Heterochromatin; low signal	None	A	0.5872	0.1187	7.60E-07	++	0	0.6014
rs79670124	*JARID2*	6	15246022	Upstream gene variant	Active_Promoter	Active_Promoter	Proximal Enhancer	A	0.5872	0.1187	7.60E-07	++	0	0.6014
rs79851650	*AL050335.1*	6	15233151	Intergenic region	Weak_Enhancer	Heterochromatin; low signal	None	A	0.5872	0.1187	7.60E-07	++	0	0.6014
6:15252987	*JARID2*	6	15252987	Upstream gene variant	Weak_Promoter	Transcriptional transition	None	GT	0.5872	0.1188	7.68E-07	++	0	0.6015
rs6904999	*AL050335.1*	6	15232306	Intergenic region	Heterochromatin; low signal	Heterochromatin; low signal	None	G	0.5872	0.1188	7.68E-07	++	0	0.6015
rs114742337	*RP4-771M4.2*	1	63383903	Intergenic region	Heterochromatin; low signal	Weak_Enhancer	None	A	0.6561	0.1329	7.98E-07	++	0	0.5052
rs7262931	*SGK2*	20	42188050	Intron variant	Heterochromatin; low signal	Weak transcribed	None	C	0.4724	0.096	8.70E-07	++	0	0.5563
rs56176153	*Y_RNA*	2	138561026	Intergenic region	Heterochromatin; low signal	Heterochromatin; low signal	None	T	0.3454	0.0702	8.74E-07	++	0	0.9848
rs11739583	*RP11-889L3.1*	5	177504116	Intergenic region	Weak transcribed	Weak transcribed	None	G	0.2632	0.0535	8.77E-07	++	68.2	0.07602
rs11748761	*RP11-889L3.1*	5	177504158	Intergenic region	Weak transcribed	Weak transcribed	None	A	0.2623	0.0534	9.01E-07	++	66.2	0.08525
rs377699726	*RNU6*	6	15316243	Downstream gene variant	Strong_Enhancer	Transcriptional transition	None	CTT	0.6234	0.1272	9.49E-07	++	0	0.7513
rs138338441	*AC092684.1*	2	164969273	Intron variant	Heterochromatin; low signal	Heterochromatin; low signal	None	T	1.1112	0.2269	9.77E-07	++	0	0.5696

rs61768462	*RP3-357I16.1*	1	30727316	Intergenic region	Heterochromatin; low signal	Weak_Enhancer	None	T	0.7401	0.1512	9.82E-07	++	0	0.3349
6:15226060	*AL050335.1*	6	15226060	Intergenic region	Heterochromatin; low signal	Weak transcribed	None	T	0.5701	0.1167	1.04E-06	++	0	0.5062
rs112727302	*AL050335.1*	6	15225220	Intergenic region	Heterochromatin; low signal	Weak transcribed	None	A	0.5701	0.1167	1.04E-06	++	0	0.5062
rs73357720	*AL050335.1*	6	15225177	Intergenic region	Heterochromatin; low signal	Weak transcribed	None	A	0.5701	0.1167	1.04E-06	++	0	0.5062
rs9476802	*AL050335.1*	6	15221584	Intergenic region	Weak transcribed	Heterochromatin; low signal	None	T	0.5701	0.1167	1.04E-06	++	0	0.5062
rs112468787	*AL050335.1*	6	15220952	Intergenic region	Weak transcribed	Heterochromatin; low signal	None	G	0.5701	0.1168	1.05E-06	++	0	0.5063
rs12525768	*AL050335.1*	6	15223452	Intergenic region	Weak_Enhancer	Polycomb-repressed	None	G	0.5701	0.1168	1.05E-06	++	0	0.5063
rs73357717	*AL050335.1*	6	15225083	Intergenic region	Heterochromatin; low signal	Weak transcribed	None	G	0.5701	0.1168	1.05E-06	++	0	0.5063
rs73357719	*AL050335.1*	6	15225118	Intergenic region	Heterochromatin; low signal	Weak transcribed	None	C	0.5701	0.1168	1.05E-06	++	0	0.5063
rs9464775	*AL050335.1*	6	15224024	Intergenic region	Heterochromatin; low signal	Heterochromatin; low signal	None	G	0.5701	0.1168	1.05E-06	++	0	0.5063
rs7806790	*EGFR*	7	55107056	Intron variant	Heterochromatin; low signal	Heterochromatin; low signal	None	C	0.765	0.1568	1.06E-06	++	0	0.4572
rs34890405	*RP11-889L3.1*	5	177508458	Intergenic region	Weak transcribed	Weak_Enhancer	None	C	0.2623	0.0538	1.09E-06	++	71.7	0.0602
rs34893110	*RP11-889L3.1*	5	177509467	Intergenic region	Weak transcribed	Weak transcribed	None	T	0.2623	0.0538	1.09E-06	++	71.7	0.0602
rs4976806	*RP11-889L3.1*	5	177507767	Intergenic region	Weak transcribed	Weak transcribed	None	T	0.2623	0.0538	1.09E-06	++	71.7	0.0602
rs4976807	*RP11-889L3.1*	5	177507914	Intergenic region	Weak transcribed	Weak transcribed	None	G	0.2624	0.0538	1.09E-06	++	71.7	0.06031
rs4976808	*RP11-889L3.1*	5	177507917	Intergenic region	Weak transcribed	Weak transcribed	None	G	0.2624	0.0538	1.09E-06	++	71.7	0.06031
rs58184656	*RP11-889L3.1*	5	177508895	Intergenic region	Weak transcribed	Weak_Enhancer	None	C	0.2624	0.0538	1.09E-06	++	71.7	0.06031
rs62390501	*RP11-889L3.1*	5	177508392	Intergenic region	Weak transcribed	Weak transcribed	None	A	0.2623	0.0538	1.09E-06	++	71.7	0.0602
rs62390504	*RP11-889L3.1*	5	177508944	Intergenic region	Weak transcribed	Weak_Enhancer	None	G	0.2624	0.0538	1.09E-06	++	71.7	0.06031
rs62390530	*RP11-889L3.1*	5	177509459	Intergenic region	Weak transcribed	Weak transcribed	None	G	0.2624	0.0538	1.09E-06	++	71.7	0.06031
rs67596535	*RP11-889L3.1*	5	177509098	Intergenic region	Weak transcribed	Weak transcribed	None	C	0.2624	0.0538	1.09E-06	++	71.7	0.06031
rs113065758	*AL050335.1*	6	15212401	Intergenic region	Heterochromatin; low signal	Heterochromatin; low signal	None	A	0.5771	0.1187	1.17E-06	++	0	0.5392
rs150477486	*FYN*	6	112024290	Upstream gene variant	Transcriptional elongation	Heterochromatin; low signal	None	T	0.9531	0.1961	1.17E-06	++	55.9	0.1321
rs13177461	*RP11-889L3.1*	5	177509545	Intergenic region	Weak transcribed	Weak transcribed	None	T	0.2614	0.0538	1.21E-06	++	71.3	0.06207
rs61271709	*AL050335.1*	6	15228099	Intergenic region	Heterochromatin; low signal	Weak_Enhancer	None	A	0.5664	0.1173	1.37E-06	++	0	0.4831
rs77908444	*AL050335.1*	6	15225071	Intergenic region	Heterochromatin; low signal	Weak transcribed	None	T	0.5686	0.118	1.44E-06	++	0	0.4926
rs113782517	*AL050335.1*	6	15222906	Intergenic region	Weak transcribed	Polycomb-repressed	None	G	0.5685	0.118	1.46E-06	++	0	0.4927
rs77409453	*JARID2*	6	15507560	Intron variant	Transcriptional elongation	Transcriptional transition	None	T	0.5548	0.1153	1.51E-06	++	0	0.7223
rs114272033	*ISCA1P2*	1	229192813	Intergenic region	Polycomb-repressed	Heterochromatin; low signal	None	A	0.9228	0.1921	1.55E-06	++	0	0.9938
rs77206482	*CES5A*	16	56026433	Intergenic region	Heterochromatin; low signal	Heterochromatin; low signal	None	T	0.6131	0.128	1.67E-06	++	45.3	0.1764

SNP, Single Nucleotide Polymorphisms; Chr, Chromosome; A1, Effect allele; MAF, Minor Allele Frequency; SE, Standard error; Dir, Effect direction; Het ChiSq, Chi-square value for heterogeneity test; Het PVal, *P*-value for heterogeneity in effect sizes in meta-analysis.

Supplementary Table 5. Demographic characteristic for whole genome sequenced rare variant cohort

Cohort	N	Dementia (%)	Age (y, mean ± SD)	Female (n, %)	Years of education (y, mean ± SD)	Non-Hispanic Whites (n, %)	Tau SUVR (mean ± SD)
A4	336	0	71.65 (4.68)	204 (60.71%)	16.39 (2.73)	323 (96.13%)	1.15 (0.09)
ADNI	180	0	71.74 (6.62)	84 (46.67%)	16.48 (2.62)	174 (96.67%)	1.12 (0.11)
HABS-HD	616	0	69.22 (8.35)	365 (59.25%)	13.93 (4.04)	412 (66.88%)	1.07 (0.12)
NACC	87	54.35%	67.61 (10.51)	46 (52.87%)	16.04 (3.17)	87 (91.95%)	1.47 (0.46)
WRAP	342	0.003%	69.78 (7.14)	195 (57.01%)	16.66 (2.77)	293 (85.67)%)	1.05 (0.16)

A4, Anti-Amyloid Treatement in Asymptomatic Alzheimer's Disease; ADNI, Alzheimer's Disease Neuroimaging Initiative; HABS-HD, Health and Aging Brain Study-Health Disparities; NACC, National Alzheimer's Coordinating Centers; WRAP, Wisconsin Registry for Alzheimer's Prevention

Supplementary Table 6. Rare variant analysis in the *JARID2* gene region using harmonized tau-SUVR

#CHROM	ID	POS	REF	ALT	VARIANT_TYPE	Overlapping regulatory region			GENE	NMISS	BETA	SE	P
						Lymphoblastoid cell line	Embryonic stem cells	Adult human brain tissues					
6	chr6_15257832_A_G	15257832	A	G	Intron variant	Weak_Enhancer	Transcriptional transition	None	*JARID2*	1533	0.2751	0.06904	7.08E-05
6	chr6_15389944_A_G	15389944	A	G	Intron variant	Strong_Enhancer	Transcriptional transition	Distal Enhancer	*JARID2*	1533	0.1824	0.04892	2.00E-04
6	chr6_15437117_C_T	15437117	C	T	Intron variant	Strong_Enhancer	Transcriptional transition	None	*JARID2*	1533	0.1824	0.04892	2.00E-04
6	chr6_15494398_A_T	15494398	A	T	Intron variant	Transcriptional elongation	Transcriptional transition	None	*JARID2*	1533	0.1044	0.03178	1.04E-03
6	chr6_15390470_T_G	15390470	T	G	Intron variant	Strong_Enhancer	Transcriptional transition	Distal Enhancer	*JARID2*	1533	0.2076	0.06914	2.73E-03
6	chr6_15420522_T_C	15420522	T	C	Intron variant	Weak transcribed	Transcriptional transition	None	*JARID2*	1533	0.1175	0.04011	3.44E-03
6	chr6_15269918_C_T	15269918	C	T	Intron variant	Strong_Enhancer	Transcriptional transition	None	*JARID2*	1533	0.1704	0.05849	3.62E-03
6	chr6_15377901_C_T	15377901	C	T	Intron variant	Transcriptional elongation	Transcriptional transition	None	*JARID2*	1533	0.172	0.06316	6.53E-03
6	chr6_15430992_G_A	15430992	G	A	Intron variant	Weak_Enhancer	Transcriptional transition	None	*JARID2*	1533	0.172	0.06316	6.53E-03
6	chr6_15515659_G_C	15515659	G	C	Intron variant	Transcriptional elongation	Transcriptional transition	None	*JARID2*	1533	0.172	0.06316	6.53E-03
6	chr6_15415240_C_T	15415240	C	T	Intron variant	Weak transcribed	Weak transcribed	None	*JARID2*	1533	0.1644	0.06924	0.02
6	chr6_15473817_C_T	15473817	C	T	Intron variant	Transcriptional elongation	Transcriptional transition	None	*JARID2*	1533	0.1644	0.06924	0.02
6	chr6_15350512_G_A	15350512	G	A	Intron variant	Transcriptional elongation	Transcriptional transition	None	*JARID2*	1533	0.1382	0.05853	0.02
6	chr6_15386459_C_T	15386459	C	T	Intron variant	Strong_Enhancer	Transcriptional transition	None	*JARID2*	1532	-0.1295	0.055	0.02
6	chr6_15272559_A_G	15272559	A	G	Intron variant	Weak_Enhancer	Transcriptional transition	None	*JARID2*	1533	-0.05676	0.02547	0.03
6	chr6_15478159_G_A	15478159	G	A	Intron variant	Transcriptional elongation	Transcriptional transition	None	*JARID2*	1533	-0.1089	0.04909	0.03
6	chr6_15407647_G_A	15407647	G	A	Intron variant	Weak_Enhancer	Transcriptional transition	None	*JARID2*	1533	0.06906	0.03187	0.03
6	chr6_15283057_T_C	15283057	T	C	Intron variant	Weak transcribed	Transcriptional transition	None	*JARID2*	1533	-0.05265	0.0245	0.03
6	chr6_15396545_C_T	15396545	C	T	Upstream gene variant	Weak transcribed	Transcriptional transition	None	*JARID2*	1533	-0.09011	0.04306	0.04
6	chr6_15509490_G_A	15509490	G	A	Downstream gene variant	Transcriptional elongation	Transcriptional transition	None	*JARID2*	1533	-0.05947	0.02853	0.04
6	chr6_15263741_C_T	15263741	C	T	Intron variant	Strong_Enhancer	Transcriptional transition	None	*JARID2*	1533	0.08851	0.04359	0.04
6	chr6_15381951_C_A	15381951	C	A	Intron variant	Weak_Enhancer	Transcriptional transition	None	*JARID2*	1533	-0.1395	0.06931	0.04
6	chr6_15410144_G_A	15410144	G	A	Intron variant	Strong_Enhancer	Transcriptional transition	None	*JARID2*	1533	-0.1395	0.06931	0.04
6	chr6_15413144_C_T	15413144	C	T	Intron variant	Transcriptional elongation	Transcriptional transition	None	*JARID2*	1533	-0.1395	0.06931	0.04
6	chr6_15477288_G_T	15477288	G	T	Intron variant	Transcriptional elongation	Transcriptional transition	None	*JARID2*	1533	-0.1395	0.06931	0.04
6	chr6_15438357_C_G	15438357	C	G	Intron variant	Weak_Enhancer	Transcriptional transition	None	*JARID2*	1533	0.1381	0.06927	0.05
6	chr6_15512628_A_G	15512628	A	G	Downstream gene variant	Transcriptional elongation	Transcriptional transition	Distal Enhancer	*JARID2*	1533	-0.08269	0.04151	0.05
6	chr6_15366490_T_G	15366490	T	G	Intron variant	Strong_Enhancer	Transcriptional transition	None	*JARID2*	1531	0.156	0.07872	0.05
6	chr6_15450757_C_T	15450757	C	T	Upstream gene variant	Weak transcribed	Transcriptional transition	None	*JARID2*	1533	-0.108	0.05484	0.05
6	chr6_15359914_T_G	15359914	T	G	Intron variant	Strong_Enhancer	Transcriptional transition	None	*JARID2*	1533	0.0716	0.03677	0.05
6	chr6_15370497_C_A	15370497	C	A	Upstream gene variant	Transcriptional elongation	Transcriptional transition	None	*JARID2*	1533	0.1346	0.06928	0.05
6	chr6_15326154_G_A	15326154	G	A	Intron variant	Strong_Enhancer	Transcriptional transition	None	*JARID2*	1533	0.09248	0.04785	0.05
6	chr6_15347717_A_G	15347717	A	G	Intron variant	Strong_Enhancer	Transcriptional transition	None	*JARID2*	1533	0.09248	0.04785	0.05

Supplementary Table 7. Rare variant meta-analysis in the *JARID2* gene region

#CHROM	MarkerName	REF	ALT	VARIANT_TYPE	Overlapping regulatory region			GENE	Effect	StdErr	*P*-value	Direction	HetISq	HetPVal
					Lymphoblastoid cell line	Embryonic stem cells	Adult human brain tissues							
6	chr6_15492808_C_T	C	T	Intron variant	Transcriptional elongation	Transcriptional transition	None	*JARID2*	-1.507714	0.249904	1.65E-09	+----	48.268767	8.30E-10
6	chr6_15376752_C_A	C	A	Intron variant	Transcriptional elongation	Transcriptional transition	None	*JARID2*	-2.397765	0.403746	2.95E-09	-?+??	17.582745	2.75E-05
6	chr6_15331726_A_G	A	G	Intron variant	Strong_Enhancer	Transcriptional transition	None	*JARID2*	-1.063304	0.182715	6.06E-09	--+?-	68.011396	1.13E-14
6	chr6_15455147_T_G	T	G	Intron variant	Transcriptional elongation	Transcriptional transition	None	*JARID2*	-1.639922	0.296926	3.41E-08	+?---	46.467382	4.51E-10
6	chr6_15465929_G_A	G	A	Intron variant	Transcriptional elongation	Transcriptional transition	None	*JARID2*	-1.639922	0.296926	3.41E-08	+?---	46.467382	4.51E-10
6	chr6_15255162_G_T	G	T	Intron variant	Strong_Enhancer	Transcriptional transition	None	*JARID2*	-1.868823	0.345866	6.69E-08	-?--+	67.67026	1.34E-14
6	chr6_15445339_C_T	C	T	Intron variant	Transcriptional elongation	Transcriptional transition	Distal Enhancer	*JARID2*	-1.235174	0.27296	6.12E-06	+?-+-	55.027606	6.77E-12
6	chr6_15459464_G_A	G	A	Intron variant	Transcriptional transition	Transcriptional transition	None	*JARID2*	-1.235174	0.27296	6.12E-06	+?-+-	55.027606	6.77E-12
6	chr6_15472111_G_A	G	A	Intron variant	Transcriptional elongation	Transcriptional transition	None	*JARID2*	-1.235174	0.27296	6.12E-06	+?-+-	55.027606	6.77E-12
6	chr6_15350225_A_G	A	G	Intron variant	Transcriptional elongation	Transcriptional transition	None	*JARID2*	-1.478342	0.32989	7.52E-06	-++?+	17.777173	4.89E-04
6	chr6_15302284_C_T	C	T	Intron variant	Strong_Enhancer	Transcriptional transition	None	*JARID2*	-1.393445	0.327131	2.07E-05	-++??	9.988466	0.006777
6	chr6_15375134_T_C	T	C	Intron variant	Transcriptional elongation	Transcriptional transition	None	*JARID2*	-0.652951	0.157609	3.47E-05	--+?+	27.61825	4.37E-06
6	chr6_15396166_T_C	T	C	Upstream gene variant	Weak transcribed	Transcriptional transition	None	*JARID2*	-0.652951	0.157609	3.47E-05	--+?+	27.61825	4.37E-06
6	chr6_15416213_C_G	C	G	Intron variant	Weak transcribed	Weak transcribed	None	*JARID2*	-0.652951	0.157609	3.47E-05	--+?+	27.61825	4.37E-06
6	chr6_15506833_C_T	T	C	Intron variant	Transcriptional elongation	Transcriptional transition	None	*JARID2*	-1.1766	0.3269	3.20E-04	?---?	45.4	0.1602
6	chr6_15427690_C_T	C	T	Intron variant	Weak_Promoter	Transcriptional transition	Distal Enhancer	*JARID2*	-1.118614	0.320756	4.91E-04	+?-+-	56.483644	3.31E-12
6	chr6_15456914_G_T	G	T	Intron variant	Weak_Enhancer	Transcriptional transition	None	*JARID2*	-0.620198	0.190017	1.11E-03	+?--+	8.192142	0.042203
6	chr6_15290651_C_T	C	T	Intron variant	Strong_Enhancer	Transcriptional transition	None	*JARID2*	-0.838262	0.261246	1.34E-03	++-?-	12.30008	0.006423
6	chr6_15297851_A_G	A	G	Intron variant	Strong_Enhancer	Transcriptional transition	None	*JARID2*	-1.422397	0.449723	1.57E-03	+?+?-	54.41434	1.53E-12
6	chr6_15422903_C_T	C	T	Intron variant	Strong_Enhancer	Transcriptional transition	Distal Enhancer	*JARID2*	-0.873307	0.284791	2.18E-03	-?+?+	58.098289	2.42E-13
6	chr6_15514635_G_A	G	A	Intron variant	Transcriptional elongation	Transcriptional transition	None	*JARID2*	-0.951667	0.333631	4.35E-03	+-+??	55.219369	1.02E-12
6	chr6_15455209_C_A	C	A	Intron variant	Transcriptional elongation	Transcriptional transition	None	*JARID2*	-1.199012	0.444207	6.97E-03	?-+??	6.038242	0.013999
6	chr6_15392718_G_T	T	G	Intron variant	Weak_Enhancer	Transcriptional transition	None	*JARID2*	-1.0871	0.436	0.01	??-+?	52.9	0.145
6	chr6_15303976_C_T	T	C	Intron variant	Weak_Enhancer	Transcriptional transition	None	*JARID2*	-0.9085	0.3701	0.01	??--+	10.5	0.3273
6	chr6_15458858_G_A	A	G	Intron variant	Strong_Enhancer	Transcriptional transition	None	*JARID2*	-1.084	0.4523	0.02	?--??	0	0.3347
6	chr6_15439339_T_G	T	G	Intron variant	Strong_Enhancer	Transcriptional transition	None	*JARID2*	1.084	0.4523	0.02	?++??	0	0.3347
6	chr6_15417131_T_A	A	T	Intron variant	Weak_Enhancer	Weak transcribed	None	*JARID2*	-1.084	0.4523	0.02	?--??	0	0.3347
6	chr6_15447301_G_T	T	G	Upstream gene variant	Weak transcribed	Transcriptional transition	None	*JARID2*	-1.084	0.4523	0.02	?--??	0	0.3347
6	chr6_15426411_C_T	T	C	Intron variant	Weak transcribed	Transcriptional transition	None	*JARID2*	-1.084	0.4523	0.02	?--??	0	0.3347
6	chr6_15438939_A_G	A	G	Intron variant	Strong_Enhancer	Transcriptional transition	None	*JARID2*	1.084	0.4523	0.02	?++??	0	0.3347
6	chr6_15365262_T_C	T	C	Intron variant	Strong_Enhancer	Transcriptional transition	None	*JARID2*	1.084	0.4523	0.02	?++??	0	0.3347
6	chr6_15446596_A_G	A	G	Intron variant	Transcriptional elongation	Transcriptional transition	None	*JARID2*	1.084	0.4523	0.02	?++??	0	0.3347
6	chr6_15378527_A_G	A	G	Intron variant	Transcriptional elongation	Transcriptional transition	None	*JARID2*	1.084	0.4523	0.02	?++??	0	0.3347
6	chr6_15468627_A_G	A	G	synonymous_variant	Transcriptional elongation	Transcriptional elongation	None	*JARID2*	1.084	0.4523	0.02	?++??	0	0.3347
6	chr6_15419528_A_T	A	T	Intron variant	Weak transcribed	Weak transcribed	None	*JARID2*	1.084	0.4523	0.02	?++??	0	0.3347
6	chr6_15413513_A_G	A	G	Intron variant	Transcriptional elongation	Transcriptional transition	None	*JARID2*	1.084	0.4523	0.02	?++??	0	0.3347
6	chr6_15363343_GCA_G	G	GCA	Intron variant	Strong_Enhancer	Transcriptional transition	None	*JARID2*	-1.084	0.4523	0.02	?--??	0	0.3347
6	chr6_15377465_C_T	T	C	Intron variant	Transcriptional elongation	Transcriptional transition	None	*JARID2*	-1.084	0.4523	0.02	?--??	0	0.3347
6	chr6_15472360_G_T	T	G	Intron variant	Transcriptional elongation	Transcriptional transition	None	*JARID2*	-1.084	0.4523	0.02	?--??	0	0.3347
6	chr6_15377566_A_G	A	G	Intron variant	Transcriptional elongation	Transcriptional transition	None	*JARID2*	1.084	0.4523	0.02	?++??	0	0.3347
6	chr6_15471345_T_C	T	C	Intron variant	Transcriptional elongation	Transcriptional transition	None	*JARID2*	1.084	0.4523	0.02	?++??	0	0.3347
6	chr6_15468458_C_T	T	C	Intron variant	Transcriptional elongation	Transcriptional elongation	None	*JARID2*	-1.084	0.4523	0.02	?--??	0	0.3347
6	chr6_15441821_T_C	T	C	Intron variant	Insulator	Transcriptional transition	None	*JARID2*	1.084	0.4523	0.02	?++??	0	0.3347
6	chr6_15384088_C_T	T	C	Intron variant	Weak transcribed	Transcriptional transition	None	*JARID2*	-1.084	0.4523	0.02	?--??	0	0.3347

6	chr6_15455317_T_C	T	C	Intron variant	Transcriptional elongation	Transcriptional transition	None	*JARID2*	1.084	0.4523	0.02	?++??	0	0.3347
6	chr6_15325875_T_A	A	T	Intron variant	Strong_Enhancer	Transcriptional transition	None	*JARID2*	-1.084	0.4523	0.02	?--??	0	0.3347
6	chr6_15409953_G_A	A	G	Intron variant	Strong_Enhancer	Transcriptional transition	None	*JARID2*	-1.084	0.4523	0.02	?--??	0	0.3347
6	chr6_15374589_C_T	T	C	Intron variant	Transcriptional elongation	Transcriptional transition	None	*JARID2*	-1.084	0.4523	0.02	?--??	0	0.3347
6	chr6_15426074_C_T	T	C	Intron variant	Weak transcribed	Transcriptional transition	None	*JARID2*	-1.084	0.4523	0.02	?--??	0	0.3347
6	chr6_15386816_C_CT	CT	C	Intron variant	Strong_Enhancer	Transcriptional transition	None	*JARID2*	-1.084	0.4523	0.02	?--??	0	0.3347
6	chr6_15419557_C_T	T	C	Intron variant	Weak transcribed	Weak transcribed	None	*JARID2*	-1.084	0.4523	0.02	?--??	0	0.3347
6	chr6_15376275_T_C	T	C	Intron variant	Transcriptional elongation	Transcriptional transition	None	*JARID2*	1.084	0.4523	0.02	?++??	0	0.3347
6	chr6_15384024_C_G	C	G	Intron variant	Weak transcribed	Transcriptional transition	None	*JARID2*	1.084	0.4523	0.02	?++??	0	0.3347
6	chr6_15383328_G_A	A	G	Intron variant	Weak_Enhancer	Transcriptional transition	None	*JARID2*	-1.084	0.4523	0.02	?--??	0	0.3347
6	chr6_15455274_A_C	A	C	Intron variant	Transcriptional elongation	Transcriptional transition	None	*JARID2*	1.084	0.4523	0.02	?++??	0	0.3347
6	chr6_15415329_C_G	C	G	Intron variant	Weak transcribed	Weak transcribed	None	*JARID2*	1.084	0.4523	0.02	?++??	0	0.3347
6	chr6_15374940_T_C	T	C	Intron variant	Transcriptional elongation	Transcriptional transition	None	*JARID2*	1.084	0.4523	0.02	?++??	0	0.3347
6	chr6_15413283_G_A	A	G	Intron variant	Transcriptional elongation	Transcriptional transition	None	*JARID2*	-1.084	0.4523	0.02	?--??	0	0.3347
6	chr6_15367519_T_G	T	G	Intron variant	Weak_Enhancer	Transcriptional transition	None	*JARID2*	1.084	0.4523	0.02	?++??	0	0.3347
6	chr6_15392787_T_C	T	C	Intron variant	Weak_Enhancer	Transcriptional transition	None	*JARID2*	1.084	0.4523	0.02	?++??	0	0.3347
6	chr6_15428057_G_A	A	G	Intron variant	Weak_Enhancer	Transcriptional transition	Distal Enhancer	*JARID2*	-1.084	0.4523	0.02	?--??	0	0.3347
6	chr6_15477229_T_C	T	C	Intron variant	Transcriptional elongation	Transcriptional transition	None	*JARID2*	1.084	0.4523	0.02	?++??	0	0.3347
6	chr6_15467462_C_T	T	C	Intron variant	Transcriptional elongation	Transcriptional transition	None	*JARID2*	-1.084	0.4523	0.02	?--??	0	0.3347
6	chr6_15379067_C_T	T	C	Intron variant	Transcriptional elongation	Transcriptional transition	None	*JARID2*	-1.084	0.4523	0.02	?--??	0	0.3347
6	chr6_15366936_G_A	A	G	Intron variant	Strong_Enhancer	Transcriptional transition	None	*JARID2*	-1.084	0.4523	0.02	?--??	0	0.3347
6	chr6_15396038_C_T	T	C	Upstream gene variant	Weak transcribed	Transcriptional transition	None	*JARID2*	-1.084	0.4523	0.02	?--??	0	0.3347
6	chr6_15461206_G_T	T	G	Intron variant	Transcriptional elongation	Transcriptional transition	None	*JARID2*	-1.084	0.4523	0.02	?--??	0	0.3347
6	chr6_15392461_G_T	T	G	Intron variant	Weak_Enhancer	Transcriptional transition	None	*JARID2*	-1.084	0.4523	0.02	?--??	0	0.3347
6	chr6_15432045_G_C	C	G	Intron variant	Strong_Enhancer	Transcriptional transition	Distal Enhancer	*JARID2*	-1.084	0.4523	0.02	?--??	0	0.3347
6	chr6_15422799_G_C	C	G	Intron variant	Strong_Enhancer	Transcriptional transition	Distal Enhancer	*JARID2*	-1.084	0.4523	0.02	?--??	0	0.3347
6	chr6_15443418_A_G	A	G	Intron variant	Weak transcribed	Transcriptional transition	Distal Enhancer	*JARID2*	1.084	0.4523	0.02	?++??	0	0.3347
6	chr6_15449044_A_G	A	G	Upstream gene variant	Weak transcribed	Transcriptional transition	None	*JARID2*	1.084	0.4523	0.02	?++??	0	0.3347
6	chr6_15425576_G_C	C	G	Intron variant	Weak transcribed	Transcriptional transition	None	*JARID2*	-1.084	0.4523	0.02	?--??	0	0.3347
6	chr6_15397500_T_C	T	C	Upstream gene variant	Weak_Enhancer	Transcriptional transition	None	*JARID2*	1.084	0.4523	0.02	?++??	0	0.3347
6	chr6_15392881_C_T	T	C	Intron variant	Weak_Enhancer	Transcriptional transition	None	*JARID2*	-1.084	0.4523	0.02	?--??	0	0.3347
6	chr6_15445718_A_AAT	A	AAT	Intron variant	Transcriptional elongation	Transcriptional transition	None	*JARID2*	1.084	0.4523	0.02	?++??	0	0.3347
6	chr6_15412339_C_T	T	C	Intron variant	Transcriptional transition	Transcriptional transition	None	*JARID2*	-1.084	0.4523	0.02	?--??	0	0.3347
6	chr6_15379524_T_C	T	C	Intron variant	Transcriptional elongation	Transcriptional transition	None	*JARID2*	1.084	0.4523	0.02	?++??	0	0.3347
6	chr6_15421835_T_C	T	C	Intron variant	Strong_Enhancer	Transcriptional transition	None	*JARID2*	1.084	0.4523	0.02	?++??	0	0.3347
6	chr6_15466998_G_C	C	G	Intron variant	Transcriptional elongation	Transcriptional transition	None	*JARID2*	-1.084	0.4523	0.02	?--??	0	0.3347
6	chr6_15408823_C_T	T	C	Intron variant	Strong_Enhancer	Transcriptional transition	None	*JARID2*	-1.084	0.4523	0.02	?--??	0	0.3347
6	chr6_15410951_T_TAAC	T	TAAC	Intron variant	Strong_Enhancer	Transcriptional transition	None	*JARID2*	1.084	0.4523	0.02	?++??	0	0.3347
6	chr6_15424633_G_A	A	G	Intron variant	Transcriptional elongation	Transcriptional transition	None	*JARID2*	-1.084	0.4523	0.02	?--??	0	0.3347
6	chr6_15417727_T_G	T	G	Intron variant	Transcriptional elongation	Weak transcribed	None	*JARID2*	1.084	0.4523	0.02	?++??	0	0.3347
6	chr6_15434861_G_A	A	G	Intron variant	Strong_Enhancer	Transcriptional transition	None	*JARID2*	-1.084	0.4523	0.02	?--??	0	0.3347
6	chr6_15456824_T_G	T	G	Intron variant	Weak_Enhancer	Transcriptional transition	None	*JARID2*	1.084	0.4523	0.02	?++??	0	0.3347
6	chr6_15375218_T_C	T	C	Intron variant	Transcriptional elongation	Transcriptional transition	None	*JARID2*	1.084	0.4523	0.02	?++??	0	0.3347
6	chr6_15417524_A_G	A	G	Intron variant	Weak transcribed	Weak transcribed	None	*JARID2*	1.084	0.4523	0.02	?++??	0	0.3347
6	chr6_15421271_G_A	A	G	Intron variant	Strong_Enhancer	Transcriptional transition	None	*JARID2*	-1.084	0.4523	0.02	?--??	0	0.3347
6	chr6_15451946_G_A	A	G	Upstream gene variant	Weak_Enhancer	Transcriptional transition	None	*JARID2*	-1.084	0.4523	0.02	?--??	0	0.3347
6	chr6_15458392_C_T	T	C	Intron variant	Strong_Enhancer	Transcriptional transition	Distal Enhancer	*JARID2*	-1.084	0.4523	0.02	?--??	0	0.3347
6	chr6_15425442_A_G	A	G	Intron variant	Weak transcribed	Transcriptional transition	None	*JARID2*	1.084	0.4523	0.02	?++??	0	0.3347

6	chr6_15424533_T_C	T	C	Intron variant	Transcriptional elongation	Transcriptional transition	None	*JARID2*	1.084	0.4523	0.02	?++??	0	0.3347
6	chr6_15444028_A_G	A	G	Intron variant	Transcriptional elongation	Transcriptional transition	None	*JARID2*	1.084	0.4523	0.02	?++??	0	0.3347
6	chr6_15452442_A_G	A	G	Intron variant	Strong_Enhancer	Transcriptional transition	None	*JARID2*	1.084	0.4523	0.02	?++??	0	0.3347
6	chr6_15457231_G_T	T	G	Intron variant	Weak_Enhancer	Transcriptional transition	None	*JARID2*	-1.084	0.4523	0.02	?--??	0	0.3347
6	chr6_15435361_G_A	A	G	Intron variant	Strong_Enhancer	Transcriptional transition	None	*JARID2*	-1.084	0.4523	0.02	?--??	0	0.3347
6	chr6_15426714_C_T	T	C	Intron variant	Transcriptional transition	Transcriptional transition	None	*JARID2*	-1.084	0.4523	0.02	?--??	0	0.3347
6	chr6_15458617_A_G	A	G	Intron variant	Strong_Enhancer	Transcriptional transition	Distal Enhancer	*JARID2*	1.084	0.4523	0.02	?++??	0	0.3347
6	chr6_15476360_G_T	T	G	Intron variant	Transcriptional elongation	Transcriptional transition	None	*JARID2*	-1.084	0.4523	0.02	?--??	0	0.3347
6	chr6_15423096_T_C	T	C	Intron variant	Strong_Enhancer	Transcriptional transition	None	*JARID2*	1.084	0.4523	0.02	?++??	0	0.3347
6	chr6_15418345_G_A	A	G	Intron variant	Transcriptional elongation	Weak transcribed	None	*JARID2*	-1.084	0.4523	0.02	?--??	0	0.3347
6	chr6_15378683_A_C	A	C	Intron variant	Transcriptional elongation	Transcriptional transition	None	*JARID2*	1.084	0.4523	0.02	?++??	0	0.3347
6	chr6_15434102_C_T	T	C	Intron variant	Strong_Enhancer	Transcriptional transition	None	*JARID2*	-1.084	0.4523	0.02	?--??	0	0.3347
6	chr6_15452469_G_C	C	G	Intron variant	Strong_Enhancer	Transcriptional transition	None	*JARID2*	-1.084	0.4523	0.02	?--??	0	0.3347
6	chr6_15458089_G_A	A	G	Intron variant	Strong_Enhancer	Transcriptional transition	None	*JARID2*	-1.084	0.4523	0.02	?--??	0	0.3347
6	chr6_15376884_T_C	T	C	Intron variant	Transcriptional elongation	Transcriptional transition	None	*JARID2*	1.084	0.4523	0.02	?++??	0	0.3347
6	chr6_15368561_G_A	A	G	Intron variant	Weak_Enhancer	Transcriptional transition	Distal Enhancer	*JARID2*	-1.084	0.4523	0.02	?--??	0	0.3347
6	chr6_15452630_A_G	A	G	Intron variant	Strong_Enhancer	Transcriptional transition	None	*JARID2*	1.084	0.4523	0.02	?++??	0	0.3347
6	chr6_15457373_C_T	T	C	Intron variant	Weak_Enhancer	Transcriptional transition	None	*JARID2*	-1.084	0.4523	0.02	?--??	0	0.3347
6	chr6_15381952_T_C	T	C	Intron variant	Weak_Enhancer	Transcriptional transition	None	*JARID2*	1.084	0.4523	0.02	?++??	0	0.3347
6	chr6_15364656_T_C	T	C	Intron variant	Strong_Enhancer	Transcriptional transition	None	*JARID2*	1.084	0.4523	0.02	?++??	0	0.3347
6	chr6_15394380_G_A	A	G	Intron variant	Weak transcribed	Transcriptional transition	None	*JARID2*	-1.084	0.4523	0.02	?--??	0	0.3347
6	chr6_15339599_G_A	A	G	Intron variant	Weak_Enhancer	Transcriptional transition	None	*JARID2*	-1.084	0.4523	0.02	?--??	0	0.3347
6	chr6_15434968_T_G	T	G	Intron variant	Strong_Enhancer	Transcriptional transition	None	*JARID2*	1.084	0.4523	0.02	?++??	0	0.3347
6	chr6_15476150_A_G	A	G	Intron variant	Transcriptional elongation	Transcriptional transition	None	*JARID2*	1.084	0.4523	0.02	?++??	0	0.3347
6	chr6_15469494_G_A	A	G	Intron variant	Transcriptional elongation	Transcriptional elongation	None	*JARID2*	-1.084	0.4523	0.02	?--??	0	0.3347
6	chr6_15371445_G_A	A	G	Upstream gene variant	Transcriptional transition	Transcriptional transition	None	*JARID2*	-1.084	0.4523	0.02	?--??	0	0.3347
6	chr6_15379007_C_T	T	C	Intron variant	Transcriptional elongation	Transcriptional transition	None	*JARID2*	-1.084	0.4523	0.02	?--??	0	0.3347
6	chr6_15378389_T_C	T	C	Intron variant	Transcriptional elongation	Transcriptional transition	None	*JARID2*	1.084	0.4523	0.02	?++??	0	0.3347
6	chr6_15249464_C_G	C	G	Upstream gene variant	Active_Promoter	Weak_Promoter	Proximal Enhance	*JARID2-AS1*	-0.886673	0.370985	0.02	+-+?-	15.680338	0.001319
6	chr6_15275417_C_A	C	A	Intron variant	Weak_Enhancer	Transcriptional transition	None	*JARID2*	-0.886673	0.370985	0.02	+-+?-	15.680338	0.001319
6	chr6_15277342_T_G	T	G	Intron variant	Weak_Enhancer	Transcriptional transition	None	*JARID2*	-0.886673	0.370985	0.02	+-+?-	15.680338	0.001319
6	chr6_15282811_G_C	G	C	Intron variant	Weak_Enhancer	Transcriptional transition	None	*JARID2*	-0.368833	0.15521	0.02	-+-++	13.331095	0.009766
6	chr6_15499225_G_T	G	T	Intron variant	Transcriptional elongation	Transcriptional transition	Distal Enhancer	*JARID2*	-0.785698	0.333044	0.02	?++?-	13.776812	0.00102
6	chr6_15416451_C_T	T	C	Intron variant	Weak transcribed	Weak transcribed	None	*JARID2*	-0.4598	0.1991	0.02	++---	15.8	0.3141
6	chr6_15244854_C_A	A	C	Upstream gene variant	Active_Promoter	Active_Promoter	Proximal Enhance	*JARID2*	-0.4515	0.1967	0.02	++-?-	35.1	0.2014
6	chr6_15329200_A_T	A	T	Intron variant	Strong_Enhancer	Transcriptional transition	None	*JARID2*	0.8384	0.3654	0.02	?+???	0	1
6	chr6_15520178_G_C	C	G	Missense_variant	Transcriptional elongation	Transcriptional elongation	None	*JARID2*	-0.7844	0.342	0.02	?+-+-	47	0.1296
6	chr6_15357286_A_G	A	G	Intron variant	Strong_Enhancer	Transcriptional transition	None	*JARID2*	-0.853315	0.373847	0.02	+-+??	8.062262	0.017754
6	chr6_15516345_T_A	A	T	Intron variant	Transcriptional elongation	Transcriptional transition	Distal Enhancer	*JARID2*	-1.0326	0.455	0.02	??-?+	34.9	0.2153
6	chr6_15432562_A_G	A	G	Intron variant	Strong_Enhancer	Transcriptional transition	None	*JARID2*	0.9889	0.4415	0.03	-?+??	71.6	0.06077
6	chr6_15499095_T_G	T	G	Intron variant	Transcriptional elongation	Transcriptional transition	Distal Enhancer	*JARID2*	0.9889	0.4415	0.03	-?+??	71.6	0.06077
6	chr6_15283278_G_A	G	A	Intron variant	Weak transcribed	Transcriptional transition	None	*JARID2*	-0.817883	0.366042	0.03	+?+?-	17.328916	1.73E-04
6	chr6_15269918_C_T	C	T	Intron variant	Strong_Enhancer	Transcriptional transition	None	*JARID2*	-0.776669	0.348235	0.03	--+??	11.954645	0.002536
6	chr6_15286818_T_C	T	C	Intron variant	Weak_Enhancer	Transcriptional transition	None	*JARID2*	-0.799279	0.371452	0.03	+?-?+	6.908821	0.031606
6	chr6_15494400_G_T	G	T	Intron variant	Transcriptional elongation	Transcriptional transition	None	*JARID2*	-0.70094	0.327345	0.03	+?+?-	7.479739	0.023757
6	chr6_15281409_A_G	A	G	Intron variant	Strong_Enhancer	Transcriptional transition	None	*JARID2*	0.8877	0.4169	0.03	?-+??	54.5	0.1384
6	chr6_15270189_A_G	A	G	Intron variant	Transcriptional transition	Transcriptional transition	None	*JARID2*	0.8877	0.4169	0.03	?-+??	54.5	0.1384
6	chr6_15258662_A_G	A	G	Intron variant	Weak_Enhancer	Transcriptional transition	None	*JARID2*	0.8877	0.4169	0.03	?-+??	54.5	0.1384

6	chr6_15264486_T_C	T	C	Intron variant	Strong_Enhancer	Weak_Promoter	Distal Enhancer	*JARID2*	0.8877	0.4169	0.03	?-+??	54.5	0.1384
6	chr6_15510000_A_G	A	G	Downstream gene variant	Transcriptional elongation	Transcriptional transition	None	*JARID2*	0.8655	0.4205	0.04	?-+?-	18.5	0.2931
6	chr6_15281174_T_A	A	T	Intron variant	Strong_Enhancer	Transcriptional transition	None	*JARID2*	-0.4156	0.2021	0.04	+?--?	0	0.4416
6	chr6_15274724_A_T	A	T	Intron variant	Weak transcribed	Transcriptional transition	None	*JARID2*	0.4156	0.2021	0.04	-?++?	0	0.4416
6	chr6_15507857_G_A	A	G	Intron variant	Transcriptional elongation	Transcriptional transition	None	*JARID2*	-0.6047	0.2951	0.04	?+---	39.2	0.1766
6	chr6_15262172_G_T	T	G	Intron variant	Weak_Enhancer	Transcriptional transition	None	*JARID2*	-0.7449	0.3636	0.04	?-???	0	1
6	chr6_15419775_A_G	A	G	Intron variant	Weak transcribed	Weak transcribed	None	*JARID2*	0.9306	0.4573	0.04	??+?-	37.1	0.2072
6	chr6_15335752_G_A	A	G	Intron variant	Strong_Enhancer	Transcriptional transition	None	*JARID2*	-0.6329	0.311	0.04	++-?-	55.8	0.07898
6	chr6_15366774_T_TTCC	T	TTCC	Intron variant	Strong_Enhancer	Transcriptional transition	None	*JARID2*	0.8215	0.4151	0.05	?++??	0	0.4991

CHROM, Chromosome; REF, Reference allele; ALT, Alternate allele, SE, Standard error; Het ChiSq, Chi-square value for heterogeneity test; Het PVal, *P* -value for heterogeneity in effect sizes in meta-analysis; MAC, Minor Allele Count; MAF, Minor Allele Frequency

Supplementary Table 8. Functional rare variant meta-analysis in the *JARID2* gene region

#CHROM	MarkerName	Allele1	Allele2	VARIANT_TYPE	GENE	Effect	StdErr	*P*-value	Direction	HetISq	HetChiSq	HetDf	HetPVal
2	chr2_38075780_C_T	T	C	splice_region_variant	*CYP1B1*	0.8673	0.3282	8.24E-03	+++?+	35.9	4.678	3	0.197
6	chr6_15468627_A_G	A	G	synonymous_variant	*JARID2*	1.084	0.4523	0.02	?++??	0	0.931	1	0.3347
6	chr6_15520178_G_C	C	G	missense_variant	*JARID2*	-0.7844	0.342	0.02	?+-+-	47	5.656	3	0.1296
2	chr2_189057315_C_A	C	A	splice_region_variant&intron_variant	*COL5A2*	-0.373286	0.1734	0.03	-?+-+	15.764707	8.09702	4	0.001267

CHROM, Chromosome; REF, Reference allele; ALT, Alternate allele, SE, Standard error; Het ChiSq, Chi-square value for heterogeneity test;
Het PVal, *P*-value for heterogeneity in effect sizes in meta-analysis; MAC, Minor Allele Count; MAF, Minor Allele Frequency

Supplementary Table 9. Tau associated variants observed in amyloid GWAS conducted by Raghavan et al., (2020)

SNP	Nearest gene	CHR	BP	Variant type	Effect	Tau-SUVR meta-analysis						Raghavan et al., β-amyloid GWAS							
						Beta	SE	*P* -value	Dir	HetISq	HetPVal	Freq	Beta	SE	*P* -value	Dir	HetISq	HetChiSq	HetPVal
rs34095326	*CTB-129P6.4*	19	45395844	upstream gene variant	A	0.289	0.0703	3.93E-05	++	0	0.9258	0.1438	1.349	0.0825	4.84E-60	+++++++++	0.6	8.049	0.4287
rs443701	*RP11-156K13.1*	8	17695793	intron variant	T	0.2657	0.0686	1.07E-04	++	0	0.5143	0.1768	0.2162	0.0754	4.13E-03	++-+++-+-	0	3.784	0.8761
rs379407	*RP11-156K13.1*	8	17698128	intron variant	T	0.2647	0.069	1.27E-04	++	0	0.5204	0.1758	0.2129	0.0756	4.83E-03	++-+++-++	0	3.537	0.8963
rs450904	*RP11-156K13.1*	8	17693500	intron variant	C	0.2647	0.069	1.27E-04	++	0	0.5204	0.1753	0.2147	0.0758	4.61E-03	++-+++-+-	0	3.105	0.9276
rs612764	*RP11-156K13.1*	8	17693204	intron variant	T	0.2647	0.069	1.27E-04	++	0	0.5204	0.176	0.2171	0.0756	4.11E-03	++-+++-+-	0	3.43	0.9046
rs387617	*RP11-156K13.1*	8	17700159	intron variant	G	0.2647	0.0691	1.27E-04	++	0	0.5204	0.8247	-0.219	0.0758	3.86E-03	--+---+--	0	3.837	0.8715
rs412206	*RP11-156K13.1*	8	17692975	intron variant	C	0.2647	0.0691	1.27E-04	++	0	0.5204	0.8242	-0.2133	0.0757	4.86E-03	--+---+-+	0	3.474	0.9012
rs431848	*RP11-156K13.1*	8	17693074	intron variant	G	0.2647	0.0691	1.27E-04	++	0	0.5204	0.8242	-0.2164	0.0757	4.23E-03	--+---+-+	0	3.438	0.904
rs850843	*RP11-156K13.1*	8	17704276	intron variant	G	0.2647	0.0691	1.27E-04	++	0	0.5204	0.825	-0.2175	0.076	4.24E-03	--+---+--	0	3.834	0.8718
rs440550	*RP11-156K13.1*	8	17696519	intron variant	G	0.2576	0.068	1.53E-04	++	0	0.5793	0.8265	-0.2185	0.0761	4.11E-03	--+---+--	0	3.821	0.8729
rs672109	*RP11-156K13.1*	8	17697233	intron variant	G	0.2576	0.068	1.53E-04	++	0	0.5793	0.8188	-0.2142	0.0748	4.18E-03	--+---+-+	0	3.543	0.8958
rs453360	*RP11-156K13.1*	8	17693321	intron variant	A	0.2588	0.0685	1.60E-04	++	0	0.568	0.1919	0.2088	0.0741	4.85E-03	++-+++-+-	0	3.629	0.889
rs396056	*RP11-156K13.1*	8	17687526	intron variant	T	0.2564	0.0685	1.82E-04	++	0	0.5873	0.1717	0.2178	0.0769	4.63E-03	++-+++-++	0	4.16	0.8424
rs3901863	*RP11-156K13.1*	8	17696742	intron variant	G	0.2509	0.068	2.27E-04	++	0	0.6343	0.8253	-0.2184	0.076	4.05E-03	--+---+--	0	3.919	0.8644
rs6857	*PVRL2*	19	45392254	3 prime UTR variant	T	0.2245	0.062	2.94E-04	++	35.4	0.2133	0.2129	1.6742	0.0685	5.79E-132	+++++++++	70.5	27.164	0.0006623
rs11203924	*FGL1*	8	17717246	downstream gene variant	A	0.2502	0.0693	3.08E-04	++	0	0.4274	0.1726	0.216	0.0761	4.53E-03	++-+++-++	0	2.825	0.9449
rs12972156	*PVRL2*	19	45387459	intron variant	G	0.2426	0.0676	3.32E-04	++	0	0.9416	0.8221	-1.4756	0.0752	8.07E-86	---------	52.6	16.894	0.03124
3:117187325	*LSAMP*	3	117187325	intron variant	G	0.3699	0.1031	3.33E-04	++	35.7	0.2124	0.9389	-0.3679	0.1231	2.80E-03	---++--+-	0	4.658	0.7934
rs7004717	*RP11-156K13.1*	8	17719243	downstream gene variant	G	0.2489	0.0694	3.34E-04	++	0	0.5125	0.8276	-0.2175	0.0762	4.32E-03	--+---+-+	0	3.12	0.9266
rs7005657	*RP11-156K13.1*	8	17719139	downstream gene variant	C	0.2489	0.0694	3.34E-04	++	0	0.5125	0.8258	-0.2168	0.0757	4.17E-03	--+---+-+	0	2.976	0.9358
rs7005819	*RP11-156K13.1*	8	17719223	downstream gene variant	C	0.2489	0.0694	3.34E-04	++	0	0.5125	0.8276	-0.2175	0.0762	4.32E-03	--+---+-+	0	3.12	0.9266
rs2055465	*RP11-156K13.1*	8	17720157	downstream gene variant	G	0.2486	0.0694	3.43E-04	++	0	0.5082	0.827	-0.2136	0.0758	4.80E-03	--+---+--	0	2.847	0.9436
rs12972970	*PVRL2*	19	45387596	intron variant	A	0.2376	0.0677	4.46E-04	++	0	0.9982	0.1781	1.4787	0.0751	2.82E-86	+++++++++	52.4	16.794	0.03233
rs77780508	*LINC00333-LINC00375*	13	85456479	intergenic region	C	0.3808	0.1087	4.59E-04	++	39.3	0.1993	0.9286	-0.3702	0.1194	1.93E-03	---++-+-+	38.1	12.924	0.1145
rs11556505	*TOMM40*	19	45396144	synonymous variant	T	0.2339	0.0668	4.64E-04	++	0	0.935	0.1771	1.5039	0.0743	4.33E-91	+++++++++	55.6	18.018	0.0211
rs2075650	*CTB-129P6.4*	19	45395619	upstream gene variant	G	0.2339	0.0668	4.65E-04	++	0	0.935	0.8226	-1.5065	0.0742	1.01E-91	---------	49.8	15.945	0.04318
rs34404554	*CTB-129P6.4*	19	45395909	upstream gene variant	G	0.2339	0.0668	4.65E-04	++	0	0.935	0.8229	-1.5049	0.0743	3.38E-91	---------	58.2	19.116	0.01425
rs13257487	*FGL1*	8	17718220	downstream gene variant	G	0.2403	0.0689	4.88E-04	++	0	0.5007	0.8249	-0.2145	0.0756	4.57E-03	--+---+-+	0	3.235	0.9188
rs11691604	*COL5A2*	2	190044765	upstream gene variant	C	0.196	0.0568	5.61E-04	++	67.4	0.07995	0.6845	-0.2004	0.0633	1.53E-03	---++---+	0	4.198	0.8388
rs7822956	*RP11-156K13.1*	8	17719930	downstream gene variant	A	0.2311	0.0679	6.69E-04	++	0	0.4363	0.1781	0.2178	0.0752	3.77E-03	++-+++-+-	0	4.535	0.8059
rs34342646	*PVRL2*	19	45388130	intron variant	A	0.2278	0.0671	6.90E-04	++	0	0.8899	0.1802	1.4599	0.0748	9.90E-85	+++++++++	48.2	15.442	0.0511
rs71352238	*CTB-129P6.4*	19	45394336	upstream gene variant	C	0.2261	0.0667	7.00E-04	++	0	0.9761	0.8214	-1.503	0.0753	1.27E-88	---------	46.4	14.925	0.06063
rs9392240	*LOC100506207*	6	8834387	intergenic region	T	0.1737	0.0538	1.24E-03	++	0	0.5161	0.6027	-0.195	0.0583	8.24E-04	------+-+	21.7	10.219	0.25
rs9393067	*LOC100506207*	6	8833202	intergenic region	T	0.1737	0.0538	1.24E-03	++	0	0.516	0.3972	0.1957	0.0582	7.77E-04	++++++-+-	24.7	10.619	0.2242
rs117667977	*LINC00333-LINC00375*	13	85484603	intergenic region	T	0.3768	0.1186	1.49E-03	++	0	0.6206	0.0438	0.4087	0.1418	3.95E-03	+++--+--+	3.6	8.301	0.4046
rs79438074	*LINC00333-LINC00375*	13	85405595	intergenic region	G	0.3722	0.118	1.61E-03	++	0	0.5934	0.9546	-0.403	0.14	3.99E-03	---++-++-	23.9	10.512	0.2309
rs4837089	*MVB12B*	9	129227037	intron variant	A	-0.2439	0.0776	1.66E-03	--	0	0.7216	0.1295	0.2506	0.0863	3.67E-03	+++++-++-	0	7.787	0.4545
rs56758793	*SEC63P2*	4	35247976	intergenic region	T	0.2628	0.0843	1.82E-03	++	0	0.9266	0.1165	0.3072	0.0905	6.85E-04	+++++++++	26.3	10.851	0.2103
rs59525808	*snoU13-SEC63P2*	4	35248331	intergenic region	A	0.2628	0.0843	1.82E-03	++	0	0.9266	0.1179	0.2939	0.0905	1.16E-03	+++++++++	24.7	10.622	0.2241
rs9505548	*RP11-314C16.1-RP11-354I10.1*	6	8832801	intergenic region	T	0.1669	0.0537	1.88E-03	++	0	0.5136	0.6125	-0.1829	0.0585	1.76E-03	------+-+	0	7.53	0.4807
rs77960098	*LINC00333-LINC00375*	13	85370434	intergenic region	T	0.3622	0.1167	1.91E-03	++	0	0.5379	0.0619	0.3854	0.1295	2.91E-03	+++--+-+-	12.4	9.132	0.3313
rs2875427	*SRGAP3*	3	9283299	intron variant	C	0.1609	0.0521	2.03E-03	++	73.3	0.05284	0.6224	-0.1681	0.0594	4.66E-03	--++++-+-	0	4.641	0.7952
rs34137533	*CTD-2555K7.2*	14	23218022	intron variant	G	0.1577	0.0515	2.21E-03	++	0	0.9595	0.5454	-0.163	0.0578	4.84E-03	--+-++--+	52.3	16.758	0.03272
rs12974942	*PVRL2*	19	45352487	intron variant	T	0.1534	0.0502	2.26E-03	++	32	0.2253	0.5561	-0.2123	0.0582	2.67E-04	-----+---	8.4	8.737	0.3649
rs1883151	*RP3-448I9.1-RP11-330A16.1*	6	14541122	intergenic region	T	0.2452	0.0804	2.28E-03	++	52.2	0.1483	0.1224	0.2505	0.0892	4.98E-03	++++-+-++	0	2.984	0.9353
rs4715914	*RP3-448I9.1-RP11-330A16.1*	6	14541534	intergenic region	T	0.2452	0.0804	2.29E-03	++	52.1	0.1483	0.8776	-0.2505	0.0892	4.98E-03	----+-+--	0	2.984	0.9353
rs72679883	*CTD-2555K7.2*	14	23206251	intron variant	A	0.1559	0.0514	2.44E-03	++	0	0.6127	0.4381	0.1661	0.0582	4.33E-03	++-+--++-	47.7	15.303	0.05352
rs157581	*TOMM40*	19	45395714	synonymous variant	C	0.1804	0.06	2.65E-03	++	0	0.3608	0.7282	-1.2821	0.0638	1.05E-89	---------	71.1	27.686	0.0005378
rs17723452	*ANO2*	12	5760171	intron variant	T	-0.3321	0.1107	2.69E-03	--	0	0.452	0.0551	0.3558	0.1258	4.68E-03	++++-----	0	7.594	0.4741
rs11857760	*SEMA6D*	15	47990840	intron variant	A	0.1628	0.0543	2.71E-03	++	28	0.2387	0.3515	0.2046	0.0605	7.26E-04	+++-++++-	17	9.637	0.2915
rs8096254	*CABLES1*	18	20713215	upstream gene variant	G	-0.1786	0.0597	2.80E-03	--	0	0.8912	0.738	0.1914	0.0664	3.95E-03	++-+--+++	0	3.624	0.8894

rs72866024	*XDH-SRD5A2*	2	31679649	intergenic region	C	0.5574	0.1865	2.80E-03	++	0	0.8775	0.9789	0.5719	0.202	4.65E-03	+-++++-++	6.2	8.53	0.3834
rs17078360	*LINC00333-LINC00375*	13	85149478	intergenic region	A	0.3424	0.1153	2.97E-03	++	0	0.8055	0.0473	0.3885	0.1366	4.44E-03	+++--+---	14.8	9.394	0.3102
rs77767193	*LINC00333-LINC00375*	13	85150865	intergenic region	G	0.3424	0.1153	2.97E-03	++	0	0.8055	0.9527	-0.3865	0.1366	4.66E-03	---++-+++	12.6	9.154	0.3294
rs77884520	*LINC00333-LINC00375*	13	85151382	intergenic region	G	0.3424	0.1153	2.97E-03	++	0	0.8055	0.9527	-0.3864	0.1361	4.52E-03	---++-+++	0	7.653	0.4681
rs8061878	*RP11-2C15.1*	16	75876366	intron variant	G	-0.242	0.0817	3.04E-03	--	0	0.3829	0.8562	0.2921	0.0881	9.14E-04	+--+++-++	14.8	9.394	0.3102
rs116920155	*UBE2V1P10-STK33P1*	9	88040790	intergenic region	G	0.3213	0.1085	3.06E-03	++	11.2	0.2885	0.9542	-0.4641	0.1392	8.57E-04	--+-+-+--	0	3.671	0.8855
rs13333319	*RP11-2C15.1*	16	75884643	intron variant	A	-0.2458	0.083	3.06E-03	--	0	0.4272	0.1033	-0.2999	0.095	1.60E-03	-++---+--	0	6.632	0.5768
rs34875347	*RP11-2C15.1*	16	75883857	intron variant	A	-0.2458	0.083	3.06E-03	--	0	0.4272	0.1033	-0.2999	0.095	1.60E-03	-++---+--	0	6.632	0.5768
rs35155329	*RP11-2C15.1*	16	75883867	intron variant	A	-0.2458	0.083	3.06E-03	--	0	0.4272	0.1033	-0.2999	0.095	1.60E-03	-++---+--	0	6.632	0.5768
rs157582	*CTB-129P6.4*	19	45396219	upstream gene variant	T	0.1772	0.06	3.16E-03	++	0	0.3851	0.2702	1.2992	0.0641	1.78E-91	+++++++++	71.8	28.341	0.0004136
rs113498865	*snoU13-SEC63P2*	4	35231589	intergenic region	A	0.2429	0.0826	3.27E-03	++	0	0.6657	0.1283	0.2555	0.0873	3.42E-03	++-++++++	0	6.638	0.5762
rs66742906	*snoU13-SEC63P2*	4	35231266	intergenic region	G	0.2429	0.0826	3.28E-03	++	0	0.6657	0.8717	-0.2555	0.0873	3.42E-03	--+------	0	6.638	0.5762
rs68107470	*snoU13-SEC63P2*	4	35231072	intergenic region	C	0.2429	0.0826	3.28E-03	++	0	0.6657	0.8702	-0.2512	0.087	3.87E-03	--+------	0	6.336	0.6097
rs6816119	*snoU13-SEC63P2*	4	35234943	intergenic region	C	0.2429	0.0826	3.28E-03	++	0	0.6657	0.8702	-0.2501	0.087	4.03E-03	--+------	0	6.361	0.6069
rs6816663	*snoU13-SEC63P2*	4	35235185	intergenic region	G	0.2429	0.0826	3.28E-03	++	0	0.6657	0.8702	-0.2501	0.087	4.03E-03	--+------	0	6.361	0.6069
rs76888261	*RP11-2C15.1*	16	75886137	intron variant	T	-0.2438	0.0832	3.39E-03	--	0	0.4378	0.1033	-0.2974	0.095	1.75E-03	-++---+--	0	6.783	0.5602
rs283815	*TOMM40*	19	45390333	upstream gene variant	G	0.1754	0.0601	3.49E-03	++	0	0.3993	0.7276	-1.2804	0.0638	1.28E-89	---------	70	26.648	0.000813
rs11140970	*UBE2V1P10-STK33P1*	9	88059730	intergenic region	G	0.314	0.1075	3.49E-03	++	20.7	0.2615	0.9536	-0.459	0.1375	8.45E-04	--+-+-+-+	0	5.062	0.751
rs184017	*CTB-129P6.4*	19	45394969	upstream gene variant	G	0.1751	0.06	3.55E-03	++	2.5	0.3112	0.7293	-1.2735	0.0639	1.83E-88	---------	72.1	28.669	0.0003624
rs7283143	*APP*	21	27410976	intron variant	A	-0.3096	0.1066	3.68E-03	--	25.6	0.2464	0.0661	-0.338	0.1177	4.09E-03	---+-----	21	10.128	0.2561
rs59007384	*CTB-129P6.4*	19	45396665	upstream gene variant	T	0.1765	0.0609	3.77E-03	++	27.9	0.239	0.2512	1.3529	0.0648	7.02E-97	+++++++++	79.9	39.789	3.51E-06
rs16990865	*snoU13-SEC63P2*	4	35240166	intergenic region	A	0.2397	0.0828	3.78E-03	++	0	0.6398	0.1282	0.2555	0.0873	3.42E-03	++-++++++	0	6.638	0.5762
rs35525541	*RP11-2C15.1*	16	75868287	downstream gene variant	A	-0.2341	0.0812	3.94E-03	--	0	0.5116	0.15	-0.2627	0.0852	2.05E-03	-++---+--	17.4	9.689	0.2876
rs7200495	*RP11-2C15.1*	16	75871447	downstream gene variant	A	-0.2341	0.0812	3.94E-03	--	0	0.5116	0.1516	-0.2673	0.0852	1.72E-03	-++---+--	19	9.871	0.2742
rs1389570	*HNRNPUP1-KRT8P2*	14	43886107	intergenic region	G	0.2328	0.0809	4.01E-03	++	65.8	0.08748	0.8729	0.2544	0.0866	3.32E-03	++-+++-+-	19	9.874	0.274
rs59382520	*HNRNPUP1-KRT8P2*	14	43891164	intergenic region	C	0.2328	0.0809	4.01E-03	++	65.8	0.08748	0.8722	0.2509	0.0866	3.76E-03	++-+++-+-	15.6	9.48	0.3034
rs6597356	*RP11-314C16.1-RP11-354I10.1*	6	8840357	intergenic region	T	0.1548	0.0541	4.20E-03	++	0	0.6633	0.4006	0.18	0.0581	1.97E-03	++++++-+-	25.2	10.693	0.2197
rs4479681	*GLRA3*	4	175576109	intron variant	A	0.1538	0.0541	4.47E-03	++	0	0.6477	0.4221	0.1839	0.059	1.83E-03	++++++-++	0.4	8.032	0.4304
rs28573751	*RP11-2C15.1*	16	75887136	intron variant	G	-0.2305	0.0813	4.59E-03	--	0	0.5216	0.8869	0.2668	0.0914	3.50E-03	+--+++-++	1.9	8.152	0.4187
rs72811338	*RP11-805I24.4*	16	86189947	downstream gene variant	G	-0.2801	0.0997	4.96E-03	--	61.3	0.1078	0.9242	0.3483	0.11	1.54E-03	+++-+--+-	51.3	16.438	0.03652
rs10151183	*RP11-16O13.1-RP11-88E18.1*	14	43515152	intergenic region	G	0.2963	0.1055	4.97E-03	++	72.2	0.05766	0.9169	0.3324	0.1146	3.73E-03	++-+++-+-	10.1	8.897	0.351
rs76217456	*RP11-16O13.1-RP11-88E18.1*	14	43517126	intergenic region	C	0.2963	0.1055	4.97E-03	++	72.2	0.05766	0.9247	0.3819	0.1154	9.34E-04	++-+++-+-	10.7	8.954	0.3462
rs10134950	*RP11-16O13.1-RP11-88E18.1*	14	43517180	intergenic region	A	0.2963	0.1055	4.98E-03	++	72.3	0.05763	0.0823	-0.3523	0.1146	2.11E-03	--+---+-+	8.2	8.712	0.3672

SNP, Single Nucleotide Polymorphisms; Chr, Chromosome; A1, Effect allele; MAF, Minor Allele Frequency; Freq, Minor Allele Frequency; MinFreq, Minimum Minor Allele Frequency;
MaxFreq, Maximum Minor Allele Frequency; SE, Standard error; Dir, Effect direction; Het ChiSq, Chi-square value for heterogeneity test; Het PVal, *P*-value for heterogeneity in effect sizes in meta-analysis.

Supplementary Table 10. Tau associated variants observed in a previous AD GWAS conducted by Bellenguez et al., in 2022

SNP	Nearest gene	CHR	BP	Variant type	Effect	Tau-SUVR Meta-analysis								IGAP Bellenguez et al., AD GWAS			
						Beta	SE	*P* -value	Dir	HetISq	HetChiSq	HetDf	HetPVal	OR	*95% CI*	*P* -value	MAF
rs76928645	*SNORA73*	7	54941328	intergenic_region	T	-0.24	0.09	0.008	--	30.9	1.447	1	0.23	0.93	0.91-0.95	1.60E-10	0.1
rs10933431	*INPP5D*	2	233981912	upstream_gene_variant	G	-0.15	0.06	0.01	--	0	0.002	1	0.97	0.93	0.92–0.95	3.60E-18	0.23
rs12151021	*ABCA7*	19	1050874	upstream_gene_variant	A	0.12	0.05	0.03	++	45.1	1.821	1	0.18	1.1	1.09–1.12	1.60E-37	0.34
rs4985556	*IL34*	16	70694000	stop_gained	A	0.18	0.09	0.03	++	0	0.788	1	0.37	1.07	1.05–1.09	6.00E-10	0.12

SNP, Single Nucleotide Polymorphisms; Chr, Chromosome; A1, Effect allele; MAF, Minor Allele Frequency; SE, Standard error; Dir, Effect direction;
Het ChiSq, Chi-square value for heterogeneity test; Het PVal, *P* -value for heterogeneity in effect sizes in meta-analysis; OR, Odds Ratio; 95% CI, 95% Confidence Interval.

Supplementary Table 11. Tau associated variants in *JARID2, ISX* and *INTS10* genes observed in a previous AD GWAS conducted by Bellenguez et al., in 2022 and Amyloid PET GWAS conducted by Neha et al., in 2020

Tau GWAS (Discovery cohort)										AD GWAS conducted by Bellenguez et al., (2022)			Amyloid GWAS conducted by Raghavan et al., (2020)		
SNP	CHR	BP	Effect allele	Other allele	Variant type	Nearest gene	Beta	SE	*P*-value	OR	Beta	*P*-value	Beta	SE	*P*-value
rs7745729	6	15515287	G	T	Intron variant	*JARID2*	0.2061	0.079	9.09E-03	1.025	0.0248	0.04572	NA	NA	NA
rs55747333	6	15305479	A	G	Intron variant	*JARID2*	-0.1995	0.1434	0.1641	0.943	-0.0582	0.008623	NA	NA	NA
rs78823412	6	15364022	A	G	Intron variant	*JARID2*	-0.0693	0.1121	0.5365	0.967	-0.0339	0.04506	-0.0022	0.1286	0.9863
rs77007716	6	15516934	T	C	Intron variant	*JARID2*	0.0896	0.1547	0.5623	0.944	-0.0572	0.01179	NA	NA	NA
rs56111601	6	15380674	A	G	Intron variant	*JARID2*	-0.0388	0.1367	0.7765	0.953	-0.0478	0.02394	-0.1571	0.1563	0.3149
rs142286503	6	15483154	A	AC	Intron variant	*JARID2*	-0.0251	0.1357	0.8534	0.951	-0.0498	0.01939	NA	NA	NA
rs78734603	6	15490738	A	G	Intron variant	*JARID2*	-0.0251	0.1357	0.8534	0.955	-0.0456	0.02819	-0.1233	0.1545	0.4248
rs11432586	6	15499343	CT	C	Upstream gene variant	*JARID2*	-0.0234	0.1343	0.8617	0.949	-0.0527	0.01191	NA	NA	NA
rs140869333	6	15465862	CT	C	Intron variant	*JARID2*	-0.0234	0.1343	0.8617	0.95	-5.12E-02	0.01521	NA	NA	NA
rs76435383	6	15471361	T	C	Intron variant	*JARID2*	-0.0234	0.1343	0.8617	0.953	-4.82E-02	0.01873	-0.1371	0.1539	0.3732
rs77777716	6	15472816	T	G	Intron variant	*JARID2*	-0.0234	0.1343	0.8617	0.954	-0.0466	0.02308	-0.0801	0.1482	0.5889
rs75432242	6	15452760	C	T	Intron variant	*JARID2*	-0.0152	0.1365	0.9112	1.049	0.0476	0.02303	0.2032	0.1564	0.1938
rs56186721	6	15504875	T	C	Intron variant	*JARID2*	-0.0128	0.133	0.9234	0.961	-0.0399	0.04785	-0.1463	0.1513	0.3335
rs12627947	22	35290062	A	G	Intergenic region	*RP1-288L1.4-ISX*	0.1142	0.0643	0.0758	1.023	0.0232	0.0272	0.0428	0.0739	0.5625
rs5749993	22	35286321	T	G	Intergenic region	*RP1-288L1.4-ISX*	0.1142	0.0643	0.0758	1.024	0.024	0.02201	0.0384	0.0739	0.6032
rs5755402	22	35292144	T	C	Intergenic region	*RP1-288L1.4-ISX*	0.1075	0.0639	0.09261	1.022	0.0217	0.03825	0.0416	0.0738	0.5729
rs74348328	22	35282830	A	G	Intergenic region	*RP1-288L1.4-ISX*	0.1079	0.0642	0.093	1.023	0.0232	0.02643	0.0414	0.0737	0.5747
rs11704828	22	35282572	C	T	Intergenic region	*RP1-288L1.4-ISX*	0.1079	0.0642	0.09305	0.977	-0.0232	0.0263	-0.0401	0.0737	0.5858
rs9610148	22	35218492	C	A	Intergenic region	*RP1-288L1.4-ISX*	0.2009	0.1523	0.187	0.938	-0.0635	0.01261	NA	NA	NA
rs7286668	22	35442750	C	A	Intron variant	*ISX*	-0.0856	0.068	0.2085	1.031	0.0308	0.005092	-0.1007	0.076	0.1853
rs16995120	22	35353270	A	G	Intron variant	*ISX*	0.1665	0.173	0.3357	0.927	-0.0759	0.008894	NA	NA	NA
rs75694931	22	35273769	C	T	Intergenic region	*RP1-288L1.4-ISX*	0.103	0.2027	0.6115	0.942	-0.0593	0.02894	NA	NA	NA
rs111638444	22	35413972	T	C	Intron variant	*ISX*	-0.0541	0.1075	0.6146	0.963	-0.0377	0.03897	NA	NA	NA
rs12484883	22	35379526	C	T	Intron variant	*ISX*	0.0267	0.0545	0.6235	0.982	-0.0181	0.03693	NA	NA	NA
rs77117492	22	35417235	T	C	Intron variant	*ISX*	-0.0479	0.1055	0.6497	0.964	-0.0368	0.04026	NA	NA	NA
rs73166100	22	35381738	G	A	Intron variant	*ISX*	0.0167	0.0547	0.7599	0.982	-0.0177	0.04125	NA	NA	NA
rs7826068	8	19629686	A	G	Intergenic region	*CSGALNACT1-INTS10*	-0.0684	0.0568	0.2287	0.979	-0.0216	0.01494	0.0596	0.0626	0.3408

rs2119693	8	19720395	G	T	Intergenic region	*INTS10-LPL*	0.0795	0.0717	0.2673	0.974	-0.0263	0.0233	-0.0138	0.0851	0.8716
rs59347135	8	19750044	G	C	Intergenic region	*INTS10-LPL*	-0.1086	0.1216	0.3717	1.052	0.0506	0.008193	NA	NA	NA
rs12550077	8	19751399	A	G	Intergenic region	*INTS10-LPL*	-0.0511	0.0658	0.4371	1.031	0.0303	0.004546	0.0971	0.0751	0.196
rs75070501	8	19737592	G	A	Intergenic region	*INTS10-LPL*	-0.0516	0.0677	0.4454	0.968	-0.0321	0.002914	-0.0779	0.0771	0.3123
rs6586865	8	19637783	C	G	Intergenic region	*CSGALNACT1-INTS10*	-0.0483	0.0645	0.4541	0.981	-0.0196	0.04649	0.0849	0.0708	0.2304
rs6586866	8	19637784	T	A	Intergenic region	*CSGALNACT1-INTS10*	-0.0483	0.0645	0.4541	1.02	0.0194	0.04836	-0.085	0.0708	0.2297
rs17410407	8	19739243	T	G	Intergenic region	*INTS10-LPL*	-0.0504	0.0676	0.4558	1.033	3.29E-02	0.002194	0.0676	0.0769	0.3792
rs17482310	8	19746876	T	G	Intergenic region	*INTS10-LPL*	-0.0488	0.0671	0.4675	1.033	0.0325	0.002479	0.0626	0.0771	0.4166
rs4922113	8	19734951	A	G	Intergenic region	*INTS10-LPL*	-0.0488	0.0676	0.4702	1.034	0.0336	0.001793	0.0874	0.077	0.2565
rs79396901	8	19748448	C	T	Intergenic region	*INTS10-LPL*	-0.0482	0.0671	0.4726	0.969	-3.20E-02	0.00286	-0.0648	0.0771	0.4007
rs10503666	8	19744596	C	G	Intergenic region	*INTS10-LPL*	-0.0478	0.0672	0.4772	1.033	0.0324	0.002608	0.0673	0.0771	0.3826
rs112861901	8	19750951	C	T	Intergenic region	*INTS10-LPL*	-0.0466	0.0657	0.4784	0.971	-0.0295	0.005686	-0.1023	0.0751	0.1733
rs77829308	8	19741186	T	C	Intergenic region	*INTS10-LPL*	-0.0434	0.0661	0.5116	1.029	0.0287	0.006809	0.0685	0.0757	0.3657
rs17482386	8	19753521	G	A	Intergenic region	*INTS10-LPL*	-0.0419	0.0657	0.5232	0.97	-0.0301	0.004544	-0.0824	0.0752	0.2731
rs7818070	8	19734916	A	G	Intergenic region	*INTS10-LPL*	0.0307	0.0533	0.5649	0.983	-0.0172	0.03564	0.0652	0.0593	0.272
rs73607783	8	19742204	A	T	Intergenic region	*INTS10-LPL*	-0.0301	0.0636	0.6354	1.03	0.0294	0.003708	0.0691	0.0689	0.3162
rs138094414	8	19750240	C	G	Intergenic region	*INTS10-LPL*	-0.0312	0.0659	0.6355	1.029	0.0282	0.007302	0.0718	0.075	0.3383
rs11986182	8	19727447	T	A	Intergenic region	*INTS10-LPL*	-0.0309	0.0687	0.6526	0.966	-0.0348	0.001523	-0.0901	0.0768	0.2413
rs34955499	8	19747498	G	A	Intergenic region	*INTS10-LPL*	-0.0235	0.0524	0.6536	0.974	-0.026	0.001439	0.069	0.0583	0.2366
rs2197088	8	19737157	G	A	Intergenic region	*INTS10-LPL*	0.0237	0.0534	0.6567	1.018	0.0176	0.03095	-0.0696	0.0583	0.2331
rs7830586	8	19738878	G	A	Intergenic region	*INTS10-LPL*	0.0237	0.0534	0.6567	1.018	0.0177	0.03129	-0.0784	0.059	0.1842
rs717834	8	19740868	C	T	Intergenic region	*INTS10-LPL*	0.0233	0.0536	0.6645	1.019	0.0189	0.02131	-0.0604	0.0586	0.3032
rs35550741	8	19746807	G	C	Intergenic region	*INTS10-LPL*	-0.0225	0.0526	0.6689	0.974	-0.0266	0.001068	0.0653	0.0581	0.261
rs112329866	8	19716431	G	T	Intergenic region	*INTS10-LPL*	-0.0224	0.0536	0.6763	1.019	0.0188	0.02561	-0.0073	0.06	0.9032
rs1441767	8	19737279	C	A	Intergenic region	*INTS10-LPL*	0.0218	0.0535	0.6829	1.02	0.0195	0.01726	-0.0668	0.0582	0.2512
rs74549553	8	19732374	C	G	Intergenic region	*INTS10-LPL*	-0.0261	0.0654	0.69	1.031	0.0304	0.004131	0.118	0.0745	0.1132
rs28625109	8	19732587	C	T	Intergenic region	*INTS10-LPL*	0.0191	0.0541	0.7246	1.017	0.0168	0.04173	-0.0546	0.0592	0.3563
rs74974359	8	19743254	C	T	Intergenic region	*INTS10-LPL*	-0.0221	0.0652	0.7349	0.973	-0.0275	0.009308	-0.107	0.0754	0.1555
rs17091651	8	19749883	T	A	Intergenic region	*INTS10-LPL*	-0.0258	0.0889	0.7716	1.027	0.0265	0.04729	-0.0501	0.0985	0.6111
rs1031046	8	19729415	A	G	Intergenic region	*INTS10-LPL*	-0.0151	0.0528	0.7746	1.027	0.0265	0.001184	-0.089	0.0577	0.1228
rs79341217	8	19743217	T	C	Intergenic region	*INTS10-LPL*	-0.0186	0.0652	0.7751	1.029	0.0289	0.005931	0.0868	0.0748	0.2459
rs1561747	8	19733568	C	A	Intergenic region	*INTS10-LPL*	0.0153	0.0537	0.7756	1.017	0.0173	0.03481	-0.0491	0.059	0.4051
rs7828612	8	19727932	A	G	Intergenic region	*INTS10-LPL*	-0.0098	0.0529	0.8524	1.027	0.0266	0.001117	-0.0813	0.0577	0.1591
rs7822518	8	19748856	T	C	Intergenic region	*INTS10-LPL*	-0.0081	0.0529	0.8785	1.026	0.026	0.001383	-0.0657	0.0583	0.26

rs7844579	8	19748603	T	C	Intergenic region	*INTS10-LPL*	-0.0081	0.0529	0.8785	1.028	0.028	0.0005855	-0.0667	0.0584	0.2541
rs11995314	8	19752207	A	G	Intergenic region	*INTS10-LPL*	-0.0075	0.0531	0.8877	1.027	0.027	0.0009509	-0.0734	0.0577	0.2032
rs11204081	8	19748122	A	G	Intergenic region	*INTS10-LPL*	0.0031	0.0531	0.9541	1.026	0.0259	0.001516	-0.0665	0.0582	0.253
rs11446553	8	19744931	A	AG	Intergenic region	*INTS10-LPL*	-0.0022	0.053	0.9673	1.026	0.026	0.001842	NA	NA	NA
rs17391774	6	15508323	T	G	Intron variant	*JARID2*	0.4313	0.115	1.76E-04	1.003	0.0026	0.8894	0.3457	0.1404	0.0138
rs35041707	6	15508268	T	C	Intron variant	*JARID2*	-0.0291	0.0655	0.6568	1	-2.00E-04	0.9855	-0.1558	0.0706	0.02738
rs4518684	8	19728815	T	G	Intergenic region	*INTS10-LPL*	0.0948	0.066	0.151	1.008	0.0083	0.414	-0.1459	0.0725	0.0443
rs966773	8	19749287	A	G	Intergenic region	*INTS10-LPL*	0.0323	0.0563	0.566	1.004	0.0043	0.6278	-0.1343	0.063	0.03298
rs117130611	8	19713718	A	G	Downstream gene variant	*INTS10*	0.0872	0.1741	0.6165	1.034	0.0334	0.1679	0.4853	0.19	0.01065
rs1837844	8	19741755	T	C	Intergenic region	*INTS10-LPL*	0.0188	0.0569	0.7419	1.006	0.0055	0.5396	-0.1283	0.0644	0.04628
rs7012719	8	19735616	T	C	Intergenic region	*INTS10-LPL*	0.0048	0.0538	0.9287	0.998	-0.002	0.8113	-0.1159	0.0591	0.04998

SNP, Single Nucleotide Polymorphisms; CHR, Chromosome; OR, Odds Ratio

Supplementary Table 12. Gene-based meta-analysis on non-Hispanic Whites identifed genes associated with tau deposition

GENE	CHR	START	STOP	ADNI					A4					Meta-analysis					
				NSNPS	NPARAM	N	ZSTAT	*P* -value	NSNPS	NPARAM	N	ZSTAT	*P* -value	NSNPS	NPARAM	N	DATASETS	ZSTAT	Meta *P* -value
APOE	19	45409039	45412650	5	3	375	4.1814	1.45E-05	6	4	311	2.1822	0.01	6	4	686	2	4.5608	2.55E-06
CTNNA3	10	67672276	69455949	7161	123	375	4.3221	7.73E-06	7346	122	311	1.9918	0.02	7254	123	686	2	4.5366	2.86E-06
C14orf2	14	104000000	104000000	43	8	375	2.4556	7.03E-03	48	7	311	3.5787	1.73E-04	46	8	686	2	4.2252	1.19E-05
RD3L	14	104000000	104000000	6	3	375	3.3009	4.82E-04	8	3	311	2.5762	5.00E-03	7	3	686	2	4.1751	1.49E-05
TDRD9	14	104000000	105000000	388	18	375	3.0523	1.14E-03	458	20	311	2.6281	4.29E-03	423	19	686	2	4.0263	2.83E-05
ADIG	20	37209838	37217104	11	7	375	2.7202	3.26E-03	11	7	311	2.5518	5.36E-03	11	7	686	2	3.7294	9.60E-05
MYRFL	12	70274016	70352502	362	29	375	3.442	2.89E-04	367	28	311	1.7484	0.04	365	29	686	2	3.7221	9.88E-05
JARID2	6	15246206	15522273	746	43	375	1.6846	4.60E-02	777	43	311	3.5958	1.62E-04	762	43	686	2	3.6666	1.23E-04
AK8	9	136000000	136000000	413	34	375	3.7112	1.03E-04	445	37	311	1.3456	0.09	429	36	686	2	3.6499	1.31E-04
APOC1	19	45417577	45422606	12	5	375	3.5701	1.78E-04	14	7	311	1.4712	0.07	13	6	686	2	3.6302	1.42E-04
CHAF1B	21	37757689	37789125	115	13	375	3.6579	1.27E-04	118	15	311	1.3494	0.09	117	14	686	2	3.6131	1.51E-04
CDK5RAP1	20	31946645	31989375	44	10	375	2.9555	1.56E-03	58	14	311	2.0839	0.02	51	12	686	2	3.5883	1.66E-04
CCDC97	19	41816094	41830788	25	3	375	3.8023	7.17E-05	25	3	311	1.0827	0.14	25	3	686	2	3.5402	2.00E-04
PINX1	8	10622473	10697394	420	15	375	2.3587	9.17E-03	434	17	311	2.6628	3.87E-03	427	16	686	2	3.5369	2.02E-04
HNRNPUL1	19	41768391	41813811	63	8	375	4.1115	1.97E-05	68	10	311	0.67019	0.25	66	9	686	2	3.4911	2.40E-04
PPFIBP2	11	7534996	7678537	606	51	375	3.0614	1.10E-03	643	52	311	1.7928	0.04	625	52	686	2	3.4706	2.60E-04
LRRTM3	10	68685792	68861309	448	34	375	2.8748	2.02E-03	450	34	311	1.9653	0.02	449	34	686	2	3.4488	2.82E-04
VPS26A	10	70883908	70932617	135	14	375	1.7071	4.39E-02	137	15	311	3.2349	6.08E-04	136	15	686	2	3.4403	2.91E-04
DAAM1	14	59655329	59838123	616	27	375	4.0255	2.84E-05	622	26	311	0.61258	0.27	619	27	686	2	3.3887	3.51E-04
TSC1	9	136000000	136000000	106	18	375	3.0462	1.16E-03	116	19	311	1.6598	0.05	111	19	686	2	3.3698	3.76E-04
UTF1	10	135000000	135000000	5	3	375	3.5108	2.23E-04	7	4	311	1.1481	0.13	6	4	686	2	3.3687	3.78E-04
PKIB	6	123000000	123000000	682	42	375	3.3995	3.38E-04	683	38	311	1.2631	0.10	683	40	686	2	3.3639	3.84E-04
LRRC20	10	72058726	72142406	316	26	375	1.7908	3.67E-02	325	25	311	2.9891	1.40E-03	321	26	686	2	3.3366	4.24E-04
EBF4	20	2673524	2740754	198	22	375	1.5544	6.00E-02	216	25	311	3.2222	6.36E-04	207	24	686	2	3.3188	4.52E-04
FAM46B	1	27331511	27339333	7	3	375	3.2604	5.56E-04	8	3	311	1.3347	0.09	8	3	686	2	3.3093	4.68E-04
CRYGN	7	151000000	151000000	28	10	375	3.237	6.04E-04	28	10	311	1.3247	0.09	28	10	686	2	3.2853	5.09E-04

CHR, Chromosome; NSNPS, Number of Single Nucleotide Polymorphisms; NPARAM, Number of Parameters; N, Number of Samples; ZSTAT, Z-Statistics

Supplementary Table 13. Genes that are commonly associated with both tau and amyloid deposition

GENE	CHR	BP	STOP	Tau						Amyloid				
				NSNPS	NPARAM	N	DATASETS	ZSTAT	*P*-value	NSNPS	NPARAM	N	ZSTAT	*P*-value
APOE	19	45409039	45412650	6	4	686	2	4.5608	2.55E-06	2	2	3154	7.224	2.52E-13
ADIG	20	37209838	37217104	11	7	686	2	3.7294	9.60E-05	1	1	3154	2.2014	0.01
LMNA	1	156052369	156109880	144	15	686	2	3.2055	6.74E-04	103	7	3154	1.792	0.04
POLD4	11	67118236	67121067	4	3	686	2	3.1816	7.32E-04	2	1	3154	1.9642	0.02
HSF2	6	122720696	122754264	72	11	686	2	3.0855	1.02E-03	58	8	3154	1.7051	0.04
COL5A2	2	189896641	190044668	387	15	686	2	3.0732	1.06E-03	330	10	3154	2.8022	2.54E-03
YEATS2	3	183415587	183530413	222	21	686	2	2.9503	1.59E-03	147	10	3154	1.7846	0.04
SERINC1	6	122764493	122793051	74	15	686	2	2.8572	2.14E-03	51	8	3154	1.7378	0.04
ZSCAN2	15	85144249	85166947	52	14	686	2	2.8143	2.44E-03	31	9	3154	1.7429	0.04
CYP8B1	3	42913684	42917633	8	4	686	2	2.7976	2.57E-03	7	3	3154	2.3903	8.42E-03
YEATS4	12	69753490	69784576	83	10	686	2	2.7853	2.67E-03	103	8	3154	1.745	0.04
TOMM40	19	45394477	45406946	54	12	686	2	2.6495	4.03E-03	24	5	3154	11.585	2.45E-31
VWA2	10	115999013	116054259	117	20	686	2	2.6454	4.08E-03	52	9	3154	1.8328	0.03
IRF4	6	391739	411443	67	18	686	2	2.5887	4.82E-03	36	9	3154	1.8147	0.03
OGFOD2	12	123459354	123464588	7	4	686	2	2.5496	5.39E-03	4	1	3154	2.0385	0.02
AP2A1	19	50270180	50310369	105	15	686	2	2.5481	5.42E-03	67	9	3154	2.0549	0.02
SMAD4	18	48556583	48611412	59	11	686	2	2.5423	5.51E-03	33	3	3154	1.709	0.04
WDR75	2	190306159	190340287	185	7	686	2	2.5247	5.79E-03	158	5	3154	2.2056	0.01
OXA1L	14	23235731	23240998	16	7	686	2	2.4417	7.31E-03	1	1	3154	1.968	0.02
LRTM2	12	1929433	1945919	44	16	686	2	2.3858	8.52E-03	4	1	3154	2.2834	0.01
GLRA3	4	175545367	175750465	821	50	686	2	2.2578	0.01	479	34	3154	3.537	2.02E-04
ZDHHC6	10	114190058	114207301	30	6	686	2	2.2407	0.01	7	3	3154	3.0358	1.20E-03
FAM83D	20	37554955	37581703	70	12	686	2	2.2284	0.01	47	3	3154	1.6538	0.05
ZNF592	15	85291818	85349663	148	21	686	2	2.2104	0.01	96	13	3154	1.7419	0.04
ABCB9	12	123405498	123451056	48	13	686	2	2.2032	0.01	18	6	3154	2.2251	0.01
CAMK2A	5	149599054	149669403	164	25	686	2	2.1916	0.01	65	10	3154	2.816	2.43E-03
TRD	14	22891537	22935569	120	21	686	2	2.1283	0.02	88	12	3154	2.3455	9.50E-03
ANKRD13D	11	67056762	67069955	19	8	686	2	2.1221	0.02	1	1	3154	2.0044	0.02
RALGAPB	20	37101486	37207504	162	14	686	2	2.1155	0.02	46	5	3154	2.2547	0.01

ROM1	11	62380213	62382592	4	2	686	2	2.0687	0.02	3	1	3154	2.269	0.01
LOC101927260	2	112003803	112006256	7	4	686	2	2.0504	0.02	4	2	3154	2.1276	0.02
CHSY3	5	129240389	129522327	527	24	686	2	2.0062	0.02	396	16	3154	1.6699	0.05
BCL2L11	2	111878491	111926022	91	18	686	2	1.9844	0.02	56	8	3154	2.1722	0.01
C8orf22	8	49966895	49988642	40	8	686	2	1.9764	0.02	29	5	3154	1.851	0.03
FBXL7	5	15500305	15939905	1070	43	686	2	1.9752	0.02	686	26	3154	1.8968	0.03
CLCF1	11	67131635	67141648	21	7	686	2	1.9441	0.03	6	2	3154	1.9377	0.03
NUDCD1	8	110253148	110346486	253	16	686	2	1.9438	0.03	187	11	3154	2.1328	0.02
TBC1D10C	11	67171384	67177561	16	7	686	2	1.9374	0.03	4	2	3154	1.9217	0.03
XRCC2	7	152343583	152373250	84	11	686	2	1.9293	0.03	56	7	3154	1.9189	0.03
OR52R1	11	4824663	4825610	5	3	686	2	1.9292	0.03	4	2	3154	2.1856	0.01
CER1	9	14719731	14722715	15	5	686	2	1.9104	0.03	6	3	3154	2.3942	8.33E-03
SCOC	4	141178440	141303710	274	26	686	2	1.9048	0.03	126	15	3154	2.3709	8.87E-03
VSTM2L	20	36531499	36573752	125	24	686	2	1.8949	0.03	72	9	3154	1.8012	0.04
EIF2AK1	7	6061878	6098860	156	17	686	2	1.8945	0.03	116	11	3154	1.6645	0.05
FAM217A	6	4068593	4079457	36	8	686	2	1.8937	0.03	27	4	3154	2.1638	0.02
MED16	19	867961	893218	79	12	686	2	1.8623	0.03	26	4	3154	1.6901	0.05
RNF26	11	119205210	119208024	7	4	686	2	1.8404	0.03	5	3	3154	2.0448	0.02
HAVCR2	5	156512843	156536248	102	14	686	2	1.8359	0.03	48	6	3154	2.0871	0.02
PTPRN2	7	157331750	158380482	3504	161	686	2	1.7959	0.04	2211	112	3154	2.5918	4.77E-03
OR51G2	11	4935949	4936893	2	2	686	2	1.7802	0.04	2	2	3154	1.8322	0.03
SHC2	19	416583	460996	170	23	686	2	1.7752	0.04	5	2	3154	2.1573	0.02
COX7C	5	85913784	85916583	5	4	686	2	1.7664	0.04	3	2	3154	1.8381	0.03
NMB	15	85198360	85201802	12	6	686	2	1.7503	0.04	9	4	3154	2.1374	0.02
ITGA3	17	48133340	48167849	72	12	686	2	1.7458	0.04	35	5	3154	1.9965	0.02
NGEF	2	233743396	233877951	545	37	686	2	1.7328	0.04	391	31	3154	1.6607	0.05
DPYSL4	10	133998564	134019280	72	14	686	2	1.717	0.04	42	8	3154	2.4693	0.01
CENPQ	6	49431054	49460820	65	12	686	2	1.7042	0.04	44	6	3154	2.5407	5.53E-03
DCAF4	14	73393040	73428825	136	18	686	2	1.6882	0.05	109	14	3154	1.9222	0.03
SIPA1L2	1	232533711	232765907	788	60	686	2	1.664	0.05	458	30	3154	1.6851	0.05
RREB1	6	7107830	7252213	302	37	686	2	1.659	0.05	186	23	3154	1.844	0.03
ARL14	3	160394948	160396236	3	3	686	2	1.6575	0.05	2	2	3154	1.6586	0.05

CHR, Chromosome; NSNPS, Number of Single Nucleotide Polymorphisms; NPARAM, Number of Parameters; N, Number of Samples; ZSTAT, Z-Statistics

Supplementary Table 14. ***JARID2*** **gene was associated with tau deposition in an independent replication cohort**

GENE	CHR	START	STOP	Replication cohort				
				NSNPS	NPARAM	N	ZSTAT	*P*-value
JARID2	6	15246206	15522273	669	49	754	1.6702	0.047443
APOE	19	45409039	45412650	6	4	754	0.60187	0.27363
CTNNA3	10	67672276	69455949	6294	155	754	-0.24014	0.59489

CHR, Chromosome; NSNPS, Number of Single Nucleotide Polymorphisms; NPARAM, Number of Parameters; N, Number of Samples; ZSTAT, Z-Statistics

Supplementary Table 15. Mendelian Randomization on proteins with potential causal effect for tau-PET SUVR levels

Mendelian Randomization analysis													Gene-based meta-analysis on non-Hispanic Whites showing the association with tau deposition			
													Condition analysis 1*		Condition analysis 2*	
Protein	Outcome	Method	NSNP	Beta	Beta LCI	Beta UCI	SE	P-value	Intercept	Intercept SE	Intercept P-value	FDR	ZSTAT	P-value	ZSTAT	P-value
LRRFIP1	tau-PET SUVR levels	Inverse variance weighted	2	-2.64	-4.06	-1.21	0.73	2.78E-04	NA	NA	NA	0.02	1.6247	0.052115	2.6126	0.0044926
IST1	tau-PET SUVR levels	Wald ratio	1	-2.26	-3.51	-1.01	0.64	3.93E-04	NA	NA	0.905878972	0.02	2.8723	0.0020373	2.3256	0.01002
HADH	tau-PET SUVR levels	Wald ratio	1	3.47	1.47	5.47	1.02	6.83E-04	NA	NA	NA	0.02	1.7522	0.039873	2.741	0.0030631
PRTG	tau-PET SUVR levels	Inverse variance weighted	4	-0.42	-0.68	-0.15	0.13	2.07E-03	NA	NA	NA	0.04	1.5565	0.059797	2.0335	0.021002
ADGRE5	tau-PET SUVR levels	Inverse variance weighted	3	1.21	0.39	2.03	0.42	3.78E-03	NA	NA	NA	0.06	1.1179	0.1318	1.9108	0.028013
PRTG	tau-PET SUVR levels	Weighted median	4	-0.40	-0.67	-0.12	0.14	5.07E-03	NA	NA	NA	0.04	1.5565	0.059797	2.0335	0.021002
ADGRE5	tau-PET SUVR levels	Weighted median	3	1.23	0.29	2.16	0.48	0.01	NA	NA	NA	0.06	1.1179	0.1318	1.9108	0.028013
PTGR1	tau-PET SUVR levels	Inverse variance weighted	5	-0.33	-0.60	-0.05	0.14	0.02	NA	NA	NA	0.23	1.8196	0.034411	2.0252	0.021423
SERPINA9	tau-PET SUVR levels	Inverse variance weighted	6	-0.29	-0.54	-0.05	0.13	0.02	NA	NA	0.162112109	0.23	2.4041	0.0081055	2.8214	0.002391
APOE	tau-PET SUVR levels	Weighted median	7	-0.30	-0.56	-0.04	0.13	0.03	NA	NA	0.846563364	0.26	4.5608	2.55E-06	2.8444	2.22E-03
APOE	tau-PET SUVR levels	Inverse variance weighted	7	-0.29	-0.55	-0.03	0.13	0.03	NA	NA	NA	0.26	4.5608	2.55E-06	2.8444	2.22E-03
SERPINA9	tau-PET SUVR levels	Weighted median	6	-0.31	-0.59	-0.03	0.14	0.03	NA	NA	NA	0.23	2.4041	0.0081055	2.8214	0.002391
LMNB2	tau-PET SUVR levels	Wald ratio	1	3.15	0.31	6.00	1.45	0.03	NA	NA	0.798028366	0.26	1.9127	0.027895	2.4043	0.0081021
ADGRE1	tau-PET SUVR levels	Inverse variance weighted	4	-0.60	-1.15	-0.06	0.28	0.03	NA	NA	NA	0.26	1.6712	0.047345	1.7374	0.04116
ADGRE1	tau-PET SUVR levels	Weighted median	4	-0.62	-1.19	-0.04	0.29	0.04	NA	NA	NA	0.26	1.6712	0.047345	1.7374	0.04116
APOE	tau-PET SUVR levels	MR Egger	7	-0.65	-1.11	-0.19	0.23	0.04	0.213351425	0.121906925	NA	0.26	4.5608	2.55E-06	2.8444	2.22E-03
FGL1	tau-PET SUVR levels	Inverse variance weighted	10	-0.25	-0.48	-0.01	0.12	0.04	NA	NA	NA	0.32	1.2885	0.098786	2.0889	0.018358
PTGR1	tau-PET SUVR levels	Weighted median	5	-0.30	-0.61	0.02	0.16	0.06	NA	NA	0.589788384	0.23	1.8196	0.034411	2.0252	0.021423
SERPINA9	tau-PET SUVR levels	MR Egger	6	-0.97	-1.79	-0.15	0.42	0.08	0.236863779	0.140512727	NA	0.23	2.4041	0.0081055	2.8214	0.002391
BACH1	tau-PET SUVR levels	Wald ratio	1	2.04	-0.36	4.44	1.22	0.10	NA	NA	NA	0.63	0.98309	0.16278	2.1515	0.01572
CEND1	tau-PET SUVR levels	Wald ratio	1	2.41	-0.44	5.26	1.45	0.10	NA	NA	NA	0.63	1.8596	0.031473	2.1507	0.015751
FGL1	tau-PET SUVR levels	Weighted median	10	-0.23	-0.50	0.04	0.14	0.10	NA	NA	NA	0.32	1.2885	0.098786	2.0889	0.018358
BCL2L15	tau-PET SUVR levels	Inverse variance weighted	2	0.49	-0.11	1.08	0.30	0.11	NA	NA	NA	0.64	2.1729	0.014892	2.8427	0.0022368
IL5RA	tau-PET SUVR levels	Inverse variance weighted	5	-0.23	-0.53	0.07	0.15	0.13	NA	NA	NA	0.64	0.22363	0.41152	1.8726	0.03056
FABP4	tau-PET SUVR levels	Wald ratio	1	-0.92	-2.11	0.26	0.60	0.13	NA	NA	NA	0.64	0.74578	0.2279	1.6461	0.049867
SSH3	tau-PET SUVR levels	Wald ratio	1	-2.28	-5.25	0.68	1.51	0.13	NA	NA	NA	0.64	1.2007	0.11494	2.1709	0.014968
CNTN5	tau-PET SUVR levels	Inverse variance weighted	8	0.18	-0.06	0.43	0.13	0.14	NA	NA	NA	0.64	1.7408	0.040862	2.0065	0.022402
TIMP3	tau-PET SUVR levels	Weighted median	7	0.23	-0.08	0.54	0.16	0.14	NA	NA	NA	0.70	2.3056	0.010567	1.7194	0.04277
CD177	tau-PET SUVR levels	Inverse variance weighted	8	-0.12	-0.28	0.04	0.08	0.15	NA	NA	NA	0.64	0.49927	0.3088	1.6823	0.046252
PRTN3	tau-PET SUVR levels	Inverse variance weighted	5	-0.45	-1.07	0.17	0.31	0.15	NA	NA	0.425523687	0.64	2.5469	0.0054337	1.7829	0.037302
LAMA4	tau-PET SUVR levels	Inverse variance weighted	2	0.44	-0.18	1.06	0.32	0.17	NA	NA	NA	0.67	1.4182	0.078065	1.9901	0.023293
SCARA5	tau-PET SUVR levels	Weighted median	5	-0.28	-0.70	0.14	0.21	0.19	NA	NA	NA	0.83	2.9481	0.0015984	2.7688	0.0028133
RETN	tau-PET SUVR levels	Weighted median	4	0.51	-0.26	1.28	0.39	0.19	NA	NA	NA	0.78	1.9121	0.027931	1.9002	0.028705

HCLS1	tau-PET SUVR levels	Wald ratio	1	0.92	-0.47	2.32	0.71	0.20	NA	NA	NA	0.70	0.45056	0.32615	1.9813	0.023778
TIMP3	tau-PET SUVR levels	MR Egger	7	0.64	-0.20	1.47	0.43	0.20	-0.170003303	0.144467315	0.62654905	0.70	2.3056	0.010567	1.7194	0.04277
STX16	tau-PET SUVR levels	Inverse variance weighted	2	1.07	-0.56	2.69	0.83	0.20	NA	NA	NA	0.70	1.1328	0.12865	2.3374	0.0097086
PLB1	tau-PET SUVR levels	MR Egger	7	-0.26	-0.61	0.09	0.18	0.20	0.044515268	0.041455818	NA	0.78	1.2105	0.11304	1.8013	0.035826
TIMP3	tau-PET SUVR levels	Inverse variance weighted	7	0.15	-0.08	0.39	0.12	0.20	NA	NA	0.590335549	0.70	2.3056	0.010567	1.7194	0.04277
PRTG	tau-PET SUVR levels	MR Egger	4	-0.58	-1.19	0.03	0.31	0.21	0.051075372	0.088447033	NA	0.04	1.5565	0.059797	2.0335	0.021002
IL5RA	tau-PET SUVR levels	Weighted median	5	-0.21	-0.53	0.12	0.17	0.21	NA	NA	NA	0.64	0.22363	0.41152	1.8726	0.03056
IRAK4	tau-PET SUVR levels	Wald ratio	1	0.84	-0.49	2.17	0.68	0.22	NA	NA	NA	0.71	0.65877	0.25502	1.8858	0.029664
NUMB	tau-PET SUVR levels	Inverse variance weighted	2	-0.71	-1.84	0.42	0.58	0.22	NA	NA	NA	0.71	1.6066	0.05407	2.3969	0.008267
PLB1	tau-PET SUVR levels	Weighted median	7	-0.17	-0.44	0.11	0.14	0.23	NA	NA	0.882081192	0.78	1.2105	0.11304	1.8013	0.035826
DSCAM	tau-PET SUVR levels	Weighted median	6	-0.17	-0.44	0.11	0.14	0.23	NA	NA	NA	0.78	1.904	0.028454	2.8631	0.0020977
COL4A1	tau-PET SUVR levels	MR Egger	3	1.80	0.46	3.15	0.69	0.23	-0.259034825	0.094425717	NA	0.97	2.7228	0.003237	1.9286	0.026889
PRTN3	tau-PET SUVR levels	Weighted median	5	-0.40	-1.07	0.26	0.34	0.23	NA	NA	NA	0.64	2.5469	0.0054337	1.7829	0.037302
RBP7	tau-PET SUVR levels	Weighted median	3	0.27	-0.19	0.73	0.23	0.25	NA	NA	NA	0.78	1.5019	0.066559	2.1061	0.017596
IL5RA	tau-PET SUVR levels	MR Egger	5	-0.46	-1.09	0.18	0.33	0.26	0.046721877	0.059529974	NA	0.64	0.22363	0.41152	1.8726	0.03056
DTX3	tau-PET SUVR levels	Wald ratio	1	-0.67	-1.91	0.56	0.63	0.28	NA	NA	NA	0.78	0.87341	0.19122	2.1755	0.014798
CNTN5	tau-PET SUVR levels	Weighted median	8	0.15	-0.13	0.44	0.15	0.30	NA	NA	0.900593991	0.64	1.7408	0.040862	2.0065	0.022402
PSME1	tau-PET SUVR levels	Wald ratio	1	-0.62	-1.79	0.55	0.60	0.30	NA	NA	NA	0.78	1.8732	0.03052	1.6957	0.044973
NCAM2	tau-PET SUVR levels	MR Egger	7	-0.38	-1.04	0.28	0.34	0.31	0.083881056	0.075034536	NA	0.92	1.938	0.02631	2.3414	0.0096063
PLB1	tau-PET SUVR levels	Inverse variance weighted	7	-0.12	-0.36	0.11	0.12	0.31	NA	NA	NA	0.78	1.2105	0.11304	1.8013	0.035826
FKBP5	tau-PET SUVR levels	Inverse variance weighted	2	-0.27	-0.79	0.25	0.27	0.31	NA	NA	0.496751472	0.78	2.2257	0.013019	2.0952	0.018075
GAL	tau-PET SUVR levels	Weighted median	3	0.57	-0.54	1.69	0.57	0.31	NA	NA	0.405130629	0.89	1.3634	0.086376	2.0749	0.018997
TFPI	tau-PET SUVR levels	Weighted median	4	-0.21	-0.62	0.20	0.21	0.32	NA	NA	NA	0.78	1.8386	0.032987	2.0772	0.018893
CLGN	tau-PET SUVR levels	Weighted median	9	0.12	-0.12	0.35	0.12	0.32	NA	NA	0.921376279	0.90	1.4028	0.080344	2.2096	0.013565
RETN	tau-PET SUVR levels	Inverse variance weighted	4	0.42	-0.42	1.25	0.42	0.33	NA	NA	NA	0.78	1.9121	0.027931	1.9002	0.028705
PLAU	tau-PET SUVR levels	Inverse variance weighted	2	-1.03	-3.10	1.04	1.06	0.33	NA	NA	NA	0.78	1.1685	0.12129	1.7468	0.040334
NID2	tau-PET SUVR levels	Weighted median	6	0.15	-0.16	0.47	0.16	0.33	NA	NA	NA	0.79	1.6672	0.047736	2.0165	0.021876
QDPR	tau-PET SUVR levels	MR Egger	3	0.43	-0.06	0.92	0.25	0.33	-0.112798039	0.077314253	0.726289001	0.78	1.0336	0.15066	1.931	0.026741
RBP7	tau-PET SUVR levels	MR Egger	3	2.42	-0.36	5.21	1.42	0.34	-0.627357089	0.40644651	0.742776106	0.78	1.5019	0.066559	2.1061	0.017596
QDPR	tau-PET SUVR levels	Weighted median	3	0.13	-0.13	0.39	0.13	0.34	NA	NA	NA	0.78	1.0336	0.15066	1.931	0.026741
CRADD	tau-PET SUVR levels	Wald ratio	1	0.67	-0.72	2.05	0.71	0.35	NA	NA	NA	0.78	1.631	0.051446	1.6959	0.044951
CD177	tau-PET SUVR levels	Weighted median	8	-0.10	-0.30	0.10	0.10	0.35	NA	NA	NA	0.64	0.49927	0.3088	1.6823	0.046252
TFPI	tau-PET SUVR levels	Inverse variance weighted	4	-0.18	-0.55	0.19	0.19	0.35	NA	NA	NA	0.78	1.8386	0.032987	2.0772	0.018893
CDC27	tau-PET SUVR levels	Wald ratio	1	0.80	-0.91	2.51	0.87	0.36	NA	NA	0.709189221	0.78	2.7847	0.0026788	2.6379	0.004171
DSCAM	tau-PET SUVR levels	Inverse variance weighted	6	-0.16	-0.50	0.18	0.17	0.36	NA	NA	NA	0.78	1.904	0.028454	2.8631	0.0020977
CD177	tau-PET SUVR levels	MR Egger	8	-0.13	-0.39	0.13	0.13	0.36	0.008993058	0.085204311	0.532583855	0.64	0.49927	0.3088	1.6823	0.046252
TFF3	tau-PET SUVR levels	Weighted median	3	-0.46	-1.47	0.55	0.51	0.37	NA	NA	0.556779111	0.78	1.204	0.11429	1.9668	0.024601
QDPR	tau-PET SUVR levels	Inverse variance weighted	3	0.12	-0.15	0.39	0.14	0.37	NA	NA	NA	0.78	1.0336	0.15066	1.931	0.026741
PLA2G1B	tau-PET SUVR levels	Wald ratio	1	0.94	-1.15	3.03	1.07	0.38	NA	NA	0.696987143	0.78	1.4441	0.074353	2.4263	0.0076264
DCXR	tau-PET SUVR levels	Wald ratio	1	-0.66	-2.13	0.81	0.75	0.38	NA	NA	NA	0.78	1.7744	0.038001	3.1055	0.00094983

COL15A1	tau-PET SUVR levels	Inverse variance weighted	3	-0.22	-0.72	0.28	0.25	0.38	NA	NA	0.828623827	0.78	2.8437	0.0022299	2.6377	0.0041733
ADGRE1	tau-PET SUVR levels	MR Egger	4	-0.62	-1.72	0.48	0.56	0.38	0.002262077	0.059041784	0.1896309	0.26	1.6712	0.047345	1.7374	0.04116
RBP7	tau-PET SUVR levels	Inverse variance weighted	3	0.26	-0.33	0.86	0.30	0.39	NA	NA	NA	0.78	1.5019	0.066559	2.1061	0.017596
ADGRE5	tau-PET SUVR levels	MR Egger	3	1.80	-0.66	4.26	1.25	0.39	-0.05621554	0.113094709	NA	0.06	1.1179	0.1318	1.9108	0.028013
TFF3	tau-PET SUVR levels	Inverse variance weighted	3	-0.40	-1.31	0.51	0.47	0.39	NA	NA	NA	0.78	1.204	0.11429	1.9668	0.024601
PECR	tau-PET SUVR levels	Weighted median	3	-0.17	-0.56	0.23	0.20	0.40	NA	NA	NA	0.83	1.366	0.085963	1.9034	0.028497
PRELP	tau-PET SUVR levels	MR Egger	4	0.41	-0.36	1.17	0.39	0.41	-0.117147535	0.09014552	NA	0.97	2.0832	0.018617	2.0674	0.019349
CPTP	tau-PET SUVR levels	Wald ratio	1	-1.20	-4.04	1.65	1.45	0.41	NA	NA	0.902587175	0.79	0.65347	0.25673	1.7259	0.042187
LXN	tau-PET SUVR levels	Inverse variance weighted	2	-0.31	-1.05	0.44	0.38	0.42	NA	NA	0.630409303	0.79	-0.079952	0.53186	1.6928	0.045248
NID2	tau-PET SUVR levels	Inverse variance weighted	6	0.12	-0.18	0.43	0.15	0.42	NA	NA	NA	0.79	1.6672	0.047736	2.0165	0.021876
F2	tau-PET SUVR levels	MR Egger	3	-1.21	-3.07	0.66	0.95	0.42	0.20709288	0.122505233	NA	0.89	2.3201	0.010167	3.0528	0.0011336
ROBO4	tau-PET SUVR levels	Wald ratio	1	0.43	-0.67	1.53	0.56	0.45	NA	NA	NA	0.81	0.19709	0.42188	2.1645	0.015213
COL15A1	tau-PET SUVR levels	Weighted median	3	-0.20	-0.72	0.32	0.27	0.45	NA	NA	0.75495822	0.78	2.8437	0.0022299	2.6377	0.0041733
CD33	tau-PET SUVR levels	Weighted median	4	-0.10	-0.35	0.16	0.13	0.46	NA	NA	NA	0.89	0.36609	0.35715	1.7489	0.040158
SCP2	tau-PET SUVR levels	Wald ratio	1	0.80	-1.33	2.92	1.08	0.46	NA	NA	NA	0.83	2.3656	0.0090009	2.0466	0.020351
SCARA5	tau-PET SUVR levels	Inverse variance weighted	5	-0.20	-0.78	0.38	0.30	0.49	NA	NA	NA	0.83	2.9481	0.0015984	2.7688	0.0028133
PECR	tau-PET SUVR levels	Inverse variance weighted	3	-0.22	-0.86	0.41	0.32	0.49	NA	NA	0.462984394	0.83	1.366	0.085963	1.9034	0.028497
ARHGEF5	tau-PET SUVR levels	Inverse variance weighted	6	0.17	-0.32	0.65	0.25	0.49	NA	NA	0.433979844	0.83	0.14199	0.44354	2.797	0.0025788
OGFR	tau-PET SUVR levels	Wald ratio	1	-0.48	-1.92	0.95	0.73	0.51	NA	NA	NA	0.84	1.4479	0.073821	2.0457	0.020391
CLGN	tau-PET SUVR levels	MR Egger	9	0.11	-0.21	0.43	0.16	0.52	-0.057216952	0.093453399	NA	0.90	1.4028	0.080344	2.2096	0.013565
TFF3	tau-PET SUVR levels	MR Egger	3	-0.84	-2.58	0.91	0.89	0.52	0.045985361	0.080119974	0.1643011	0.78	1.204	0.11429	1.9668	0.024601
PDCD5	tau-PET SUVR levels	Inverse variance weighted	3	0.14	-0.29	0.58	0.22	0.52	NA	NA	0.888136133	0.84	1.644	0.050089	2.162	0.015311
CCL26	tau-PET SUVR levels	MR Egger	3	-9.81	-30.46	10.83	10.53	0.52	0.935719021	0.986513974	NA	0.90	1.8453	0.032496	2.08	0.018762
TFPI	tau-PET SUVR levels	MR Egger	4	-0.41	-1.45	0.63	0.53	0.52	0.051528921	0.111118067	NA	0.78	1.8386	0.032987	2.0772	0.018893
ARHGEF5	tau-PET SUVR levels	Weighted median	6	0.12	-0.28	0.53	0.20	0.54	NA	NA	NA	0.83	0.14199	0.44354	2.797	0.0025788
PDCD5	tau-PET SUVR levels	MR Egger	3	-0.49	-1.62	0.63	0.57	0.55	0.153375547	0.129204882	NA	0.84	1.644	0.050089	2.162	0.015311
ARHGEF5	tau-PET SUVR levels	MR Egger	6	0.42	-0.85	1.69	0.65	0.56	-0.087918593	0.20945377	NA	0.83	0.14199	0.44354	2.797	0.0025788
ANGPTL3	tau-PET SUVR levels	Weighted median	3	-0.13	-0.56	0.30	0.22	0.56	NA	NA	NA	0.89	2.1963	0.014034	2.0143	0.021988
TNFRSF10A	tau-PET SUVR levels	Weighted median	5	-0.10	-0.45	0.24	0.18	0.56	NA	NA	NA	0.89	1.6979	0.044761	1.708	0.043819
GRP	tau-PET SUVR levels	Weighted median	4	-0.09	-0.40	0.22	0.16	0.56	NA	NA	0.404486603	0.89	1.1095	0.1336	2.0377	0.020791
DSG3	tau-PET SUVR levels	Weighted median	3	-0.12	-0.54	0.29	0.21	0.56	NA	NA	NA	0.89	1.3413	0.089908	1.8206	0.034331
GRP	tau-PET SUVR levels	MR Egger	4	-0.17	-0.67	0.32	0.25	0.56	0.02485953	0.051334529	NA	0.89	1.1095	0.1336	2.0377	0.020791
F2	tau-PET SUVR levels	Inverse variance weighted	3	0.27	-0.65	1.19	0.47	0.56	NA	NA	NA	0.89	2.3201	0.010167	3.0528	0.0011336
PDCD5	tau-PET SUVR levels	Weighted median	3	0.10	-0.24	0.44	0.17	0.56	NA	NA	NA	0.84	1.644	0.050089	2.162	0.015311
GRP	tau-PET SUVR levels	Inverse variance weighted	4	-0.09	-0.40	0.22	0.16	0.57	NA	NA	0.450960302	0.89	1.1095	0.1336	2.0377	0.020791
SERPINA12	tau-PET SUVR levels	Weighted median	8	-0.10	-0.46	0.26	0.18	0.58	NA	NA	NA	0.89	2.2702	0.011598	1.6858	0.045914
DSG3	tau-PET SUVR levels	Inverse variance weighted	3	-0.12	-0.54	0.30	0.21	0.59	NA	NA	NA	0.89	1.3413	0.089908	1.8206	0.034331
NID2	tau-PET SUVR levels	MR Egger	6	0.17	-0.41	0.75	0.29	0.59	-0.012990589	0.063742694	NA	0.79	1.6672	0.047736	2.0165	0.021876
TCN2	tau-PET SUVR levels	Inverse variance weighted	6	0.07	-0.20	0.34	0.14	0.61	NA	NA	NA	0.89	1.3171	0.093897	1.843	0.032664
TNFRSF10A	tau-PET SUVR levels	Inverse variance weighted	5	-0.09	-0.45	0.26	0.18	0.61	NA	NA	NA	0.89	1.6979	0.044761	1.708	0.043819

TNFSF11	tau-PET SUVR levels	Wald ratio	1	0.24	-0.68	1.15	0.47	0.61	NA	NA	NA	0.89	2.5045	0.006131	3.0771	0.001045
DSG3	tau-PET SUVR levels	MR Egger	3	-0.40	-1.55	0.75	0.59	0.62	0.07656194	0.148519438	NA	0.89	1.3413	0.089908	1.8206	0.034331
PRTN3	tau-PET SUVR levels	MR Egger	5	-0.40	-1.85	1.04	0.74	0.62	-0.004739126	0.070302722	NA	0.64	2.5469	0.0054337	1.7829	0.037302
MVK	tau-PET SUVR levels	Inverse variance weighted	2	-0.42	-2.13	1.29	0.87	0.63	NA	NA	0.911736189	0.89	1.8095	0.035187	1.8278	0.033787
PVALB	tau-PET SUVR levels	Inverse variance weighted	4	0.06	-0.18	0.30	0.12	0.64	NA	NA	NA	0.89	0.83685	0.20134	1.7424	0.04072
DSCAM	tau-PET SUVR levels	MR Egger	6	-0.12	-0.62	0.37	0.25	0.65	-0.012278691	0.058083829	0.624756588	0.78	1.904	0.028454	2.8631	0.0020977
CD33	tau-PET SUVR levels	Inverse variance weighted	4	-0.07	-0.41	0.26	0.17	0.66	NA	NA	0.365452854	0.89	0.36609	0.35715	1.7489	0.040158
SCARA5	tau-PET SUVR levels	MR Egger	5	-0.27	-1.39	0.85	0.57	0.67	0.012988403	0.086815964	NA	0.83	2.9481	0.0015984	2.7688	0.0028133
SERPINB6	tau-PET SUVR levels	Inverse variance weighted	2	-0.14	-0.75	0.48	0.32	0.67	NA	NA	NA	0.89	2.5786	0.00496	1.7721	0.038193
SELP	tau-PET SUVR levels	Inverse variance weighted	3	0.08	-0.30	0.47	0.20	0.68	NA	NA	NA	0.89	0.44669	0.32755	1.7198	0.042731
TAB2	tau-PET SUVR levels	Wald ratio	1	-0.55	-3.17	2.06	1.33	0.68	NA	NA	NA	0.89	1.0848	0.13901	1.6746	0.047002
TNFRSF10A	tau-PET SUVR levels	MR Egger	5	-0.38	-2.04	1.28	0.85	0.69	0.071540862	0.205460818	0.98616895	0.89	1.6979	0.044761	1.708	0.043819
SBSN	tau-PET SUVR levels	MR Egger	4	0.25	-0.80	1.29	0.53	0.69	-0.057583454	0.091805416	NA	0.89	1.5707	0.058128	2.0592	0.01974
SERPINA12	tau-PET SUVR levels	MR Egger	8	-0.19	-1.11	0.72	0.47	0.69	0.038768859	0.128578322	NA	0.89	2.2702	0.011598	1.6858	0.045914
SERPINA12	tau-PET SUVR levels	Inverse variance weighted	8	-0.06	-0.36	0.24	0.15	0.69	NA	NA	0.648542645	0.89	2.2702	0.011598	1.6858	0.045914
F2	tau-PET SUVR levels	Weighted median	3	0.16	-0.66	0.98	0.42	0.70	NA	NA	NA	0.89	2.3201	0.010167	3.0528	0.0011336
GAL	tau-PET SUVR levels	Inverse variance weighted	3	0.19	-0.79	1.16	0.50	0.70	NA	NA	NA	0.89	1.3634	0.086376	2.0749	0.018997
ANGPTL3	tau-PET SUVR levels	Inverse variance weighted	3	-0.10	-0.62	0.42	0.27	0.71	NA	NA	NA	0.89	2.1963	0.014034	2.0143	0.021988
FGL1	tau-PET SUVR levels	MR Egger	10	-0.08	-0.51	0.34	0.22	0.71	-0.061631125	0.068757921	NA	0.32	1.2885	0.098786	2.0889	0.018358
PVALB	tau-PET SUVR levels	MR Egger	4	-0.10	-0.57	0.37	0.24	0.71	0.056099015	0.070125829	NA	0.89	0.83685	0.20134	1.7424	0.04072
SBSN	tau-PET SUVR levels	Inverse variance weighted	4	-0.07	-0.44	0.31	0.19	0.72	NA	NA	0.287205917	0.89	1.5707	0.058128	2.0592	0.01974
TCN2	tau-PET SUVR levels	Weighted median	6	0.05	-0.26	0.37	0.16	0.74	NA	NA	0.856398701	0.89	1.3171	0.093897	1.843	0.032664
CLGN	tau-PET SUVR levels	Inverse variance weighted	9	0.03	-0.16	0.22	0.10	0.75	NA	NA	NA	0.90	1.4028	0.080344	2.2096	0.013565
CCL26	tau-PET SUVR levels	Inverse variance weighted	3	0.17	-0.86	1.19	0.52	0.75	NA	NA	NA	0.90	1.8453	0.032496	2.08	0.018762
EPHA1	tau-PET SUVR levels	Inverse variance weighted	2	-0.15	-1.13	0.83	0.50	0.76	NA	NA	NA	0.90	1.1475	0.1256	2.7332	0.0031362
DPEP2	tau-PET SUVR levels	MR Egger	5	0.34	-1.66	2.35	1.02	0.76	-0.099707772	0.239159776	NA	0.93	1.1825	0.1185	1.6894	0.045574
SELP	tau-PET SUVR levels	Weighted median	3	0.06	-0.33	0.45	0.20	0.76	NA	NA	NA	0.89	0.44669	0.32755	1.7198	0.042731
PTGR1	tau-PET SUVR levels	MR Egger	5	-0.07	-0.50	0.36	0.22	0.77	-0.099649484	0.065329944	0.896105361	0.23	1.8196	0.034411	2.0252	0.021423
GAL	tau-PET SUVR levels	MR Egger	3	-0.57	-3.53	2.39	1.51	0.77	0.09306674	0.169808858	NA	0.89	1.3634	0.086376	2.0749	0.018997
COL4A1	tau-PET SUVR levels	Weighted median	3	0.08	-0.47	0.62	0.28	0.78	NA	NA	NA	0.97	2.7228	0.003237	1.9286	0.026889
NCAM2	tau-PET SUVR levels	Weighted median	7	-0.04	-0.33	0.25	0.15	0.79	NA	NA	NA	0.92	1.938	0.02631	2.3414	0.0096063
NCAM2	tau-PET SUVR levels	Inverse variance weighted	7	-0.03	-0.28	0.22	0.13	0.80	NA	NA	0.473580569	0.92	1.938	0.02631	2.3414	0.0096063
CXCL10	tau-PET SUVR levels	Inverse variance weighted	2	0.20	-1.30	1.69	0.76	0.80	NA	NA	NA	0.92	0.080748	0.46782	1.6594	0.048519
GGT5	tau-PET SUVR levels	MR Egger	4	0.16	-0.91	1.23	0.55	0.80	-0.054570606	0.187761655	0.74655753	0.99	0.90805	0.18193	1.7193	0.042784
DPEP2	tau-PET SUVR levels	Inverse variance weighted	5	-0.07	-0.64	0.51	0.29	0.82	NA	NA	NA	0.93	1.1825	0.1185	1.6894	0.045574
CNTN5	tau-PET SUVR levels	MR Egger	8	-0.06	-0.56	0.44	0.25	0.82	0.050379474	0.045788018	0.806018211	0.64	1.7408	0.040862	2.0065	0.022402
PECR	tau-PET SUVR levels	MR Egger	3	0.09	-0.62	0.80	0.36	0.84	-0.090138412	0.067731441	NA	0.83	1.366	0.085963	1.9034	0.028497
ANGPTL3	tau-PET SUVR levels	MR Egger	3	-0.25	-2.28	1.79	1.04	0.85	0.034091376	0.221481128	NA	0.89	2.1963	0.014034	2.0143	0.021988
APPL2	tau-PET SUVR levels	Inverse variance weighted	2	0.09	-0.88	1.07	0.50	0.85	NA	NA	NA	0.95	1.1341	0.12838	1.9233	0.027222
CCL26	tau-PET SUVR levels	Weighted median	3	0.09	-0.85	1.03	0.48	0.85	NA	NA	NA	0.90	1.8453	0.032496	2.08	0.018762

CD33	tau-PET SUVR levels	MR Egger	4	-0.08	-0.84	0.69	0.39	0.86	0.000387948	0.088963506	NA	0.89	0.36609	0.35715	1.7489	0.040158
PRELP	tau-PET SUVR levels	Weighted median	4	0.03	-0.38	0.44	0.21	0.88	NA	NA	NA	0.97	2.0832	0.018617	2.0674	0.019349
PRELP	tau-PET SUVR levels	Inverse variance weighted	4	-0.03	-0.42	0.36	0.20	0.88	NA	NA	NA	0.97	2.0832	0.018617	2.0674	0.019349
COL4A1	tau-PET SUVR levels	Inverse variance weighted	3	0.08	-0.99	1.14	0.54	0.89	NA	NA	NA	0.97	2.7228	0.003237	1.9286	0.026889
F10	tau-PET SUVR levels	MR Egger	4	-0.08	-1.08	0.93	0.51	0.90	0.013008077	0.066700025	NA	0.99	1.2631	0.10328	3.9946	3.24E-05
F10	tau-PET SUVR levels	Weighted median	4	-0.03	-0.52	0.47	0.25	0.91	NA	NA	NA	0.99	1.2631	0.10328	3.9946	3.24E-05
SELP	tau-PET SUVR levels	MR Egger	3	0.05	-0.73	0.84	0.40	0.92	0.008854525	0.096325064	NA	0.89	0.44669	0.32755	1.7198	0.042731
PRCP	tau-PET SUVR levels	Inverse variance weighted	2	-0.08	-1.95	1.79	0.96	0.93	NA	NA	NA	0.99	2.1949	0.014084	2.2495	0.01224
COL15A1	tau-PET SUVR levels	MR Egger	3	-0.08	-1.65	1.49	0.80	0.94	-0.020400285	0.105286388	NA	0.78	2.8437	0.0022299	2.6377	0.0041733
DPEP2	tau-PET SUVR levels	Weighted median	5	0.02	-0.61	0.66	0.32	0.94	NA	NA	0.987176131	0.93	1.1825	0.1185	1.6894	0.045574
THTPA	tau-PET SUVR levels	Wald ratio	1	0.02	-0.45	0.48	0.24	0.94	NA	NA	NA	0.99	1.5021	0.066539	2.3168	0.010258
RETN	tau-PET SUVR levels	MR Egger	4	-0.13	-3.23	2.98	1.59	0.94	0.068381631	0.190093921	0.723117122	0.78	1.9121	0.027931	1.9002	0.028705
CRYBB1	tau-PET SUVR levels	Wald ratio	1	-0.02	-0.69	0.64	0.34	0.95	NA	NA	0.80423284	0.99	1.5108	0.065422	2.8291	0.0023341
TCN2	tau-PET SUVR levels	MR Egger	6	-0.02	-0.74	0.70	0.37	0.95	0.018861072	0.06811367	NA	0.89	1.3171	0.093897	1.843	0.032664
F10	tau-PET SUVR levels	Inverse variance weighted	4	0.01	-0.46	0.49	0.24	0.96	NA	NA	NA	0.99	1.2631	0.10328	3.9946	3.24E-05
GGT5	tau-PET SUVR levels	Weighted median	4	-0.01	-0.35	0.33	0.17	0.96	NA	NA	0.906407446	0.99	0.90805	0.18193	1.7193	0.042784
GGT5	tau-PET SUVR levels	Inverse variance weighted	4	0.01	-0.31	0.33	0.16	0.97	NA	NA	NA	0.99	0.90805	0.18193	1.7193	0.042784
PVALB	tau-PET SUVR levels	Weighted median	4	0.00	-0.27	0.28	0.14	0.98	NA	NA	NA	0.89	0.83685	0.20134	1.7424	0.04072
AP1G2	tau-PET SUVR levels	Wald ratio	1	-0.02	-2.07	2.03	1.05	0.98	NA	NA	NA	0.99	1.4711	0.070632	2.5221	0.005833
S100A12	tau-PET SUVR levels	Inverse variance weighted	2	0.00	-0.46	0.45	0.23	0.99	NA	NA	0.303067034	0.99	1.046	0.14777	2.0227	0.02155
SBSN	tau-PET SUVR levels	Weighted median	4	0.00	-0.39	0.38	0.20	0.99	NA	NA	NA	0.89	1.5707	0.058128	2.0592	0.01974

NSNP, Number of Single Nucleotide; Beta LCI, Beta Lower Confidence Interval; Beta UCI, Beta Upper Confidence Interval; SE, Standard Error; FDR, False Discovery Rate

*Condition analysis 1: Adjusted for age, sex, three principal components and clinical diagnosis status

[#]Condition analysis 2: Adjusted for age, sex, three principal components, *APOE* ε4 status and clinical diagnosis status

Supplementary Table 16. Results of colocalization between FDR significant plasma proteins and tau-PET SUVR levels

HGNC.symbol	nsnps	PP.H0.abf	PP.H1.abf	PP.H2.abf	PP.H3.abf	PP.H4.abf	Exposure	UniProt	Assay	Panel
PRTG	6361	0	0.4764023	0	0.3250127	0.1985851	OID21443	Q2VWP7	PRTG	Oncology
HADH	4660	1.88E-13	0.2144864	1.49E-13	0.1694285	0.6160851	OID30132	Q16836	HADH	Cardiometabolic_II
IST1	3464	1.07E-29	0.3800568	5.17E-30	0.1836888	0.4362544	OID31497	P53990	IST1	Oncology_II
LRRFIP1	5936	6.45E-18	0.4659537	4.71E-18	0.3401388	0.1939075	OID31344	Q32MZ4	LRRFIP1	Oncology_II

nsnps, number of single nucleotide polymorphism; PP.H0.abf, Posterior probability of no causal variants in the locus; PP.H1.abf=Posterior probability of a causal variant on trait 1 (plasma proteins) only; PP.H2.abf=Posterior probability of a causal variant on trait 2 (tau-PET SUVR levels) only; PP.H3.abf=Posterior probability of distinct causal variants on trait 1 (plasma protein) and trait 2 (tau-PET SUVR levels); PP.H4.abf=Posterior probability of A shared causal variant on trait 1 (plasma protein) and trait 2 (tau-PET SUVR levels)

Supplementary Table 17. SNPs associated with plasma proteomic profiles and tau-PET SUVR levels

SNPs associated with plasma proteomic profiles of UK Biobank participants														SNP-based meta-analysis on non-Hispanic Whites showing the association with tau deposition							
SNP	CHR	POS	A2	A1	Protein	MAF	INFO	N	BETA	SE	CHISQ	*P*-value	LOG10P	VARIANT_TYPE	GENE	Condition analysis 1*			Condition analysis 2*		
																Beta	SE	*P*-value	Beta	SE	*P*-value
rs10851591	15	55711507	A	G	PRTG	0.67695	1.01297	51441	0.432841	0.00646786	4478.53	0	974.424	intron_variant	*PRTG*	0.1288	0.0542	0.01744	0.1224	0.0533	0.02152
rs2008336	15	55714806	C	T	PRTG	0.678343	1.01017	51441	0.430642	0.0064723	4427.05	0	963.244	intron_variant	*PRTG*	0.1229	0.0537	0.02218	0.1171	0.0528	0.02664
rs7162371	15	55709576	C	T	PRTG	0.671683	1.01355	51441	0.426828	0.00643256	4402.91	0	957.999	intron_variant	*PRTG*	0.1323	0.0542	0.01466	0.1269	0.0533	0.01722
rs6493811	15	55731573	G	A	PRTG	0.674262	0.986469	51441	0.432892	0.00654059	4380.52	0	953.136	intron_variant	*PRTG*	0.1195	0.0538	0.02624	0.1143	0.0529	0.03064
rs8034414	15	55721723	C	T	PRTG	0.675424	1.00896	51441	0.426946	0.00645914	4369.15	0	950.666	intron_variant	*PRTG*	0.1181	0.0539	0.02832	0.1118	0.053	0.03474
rs9672390	15	55704898	A	T	PRTG	0.669115	1.01328	51441	0.421519	0.00641876	4312.53	0	938.37	intron_variant	*PRTG*	0.1142	0.0538	0.03384	0.1101	0.0529	0.03729
rs11858195	15	55706397	A	C	PRTG	0.669359	1.01399	51441	0.421241	0.00641674	4309.55	0	937.721	intron_variant	*PRTG*	0.1153	0.0537	0.03183	0.1108	0.0528	0.03583
rs11857467	15	55706098	C	G	PRTG	0.669247	1.0136	51441	0.421248	0.00641769	4308.41	0	937.476	intron_variant	*PRTG*	0.1142	0.0538	0.03384	0.1101	0.0529	0.03729
rs11857453	15	55705882	G	T	PRTG	0.669237	1.01361	51441	0.421228	0.00641761	4308.12	0	937.412	intron_variant	*PRTG*	0.1185	0.0537	0.02732	0.114	0.0528	0.03072
rs4774803	15	55720508	T	A	PRTG	0.670613	1.00931	51441	0.421339	0.00642212	4304.33	0	936.588	intron_variant	*PRTG*	0.1215	0.0541	0.02462	0.117	0.0532	0.02773
rs7182733	15	55740777	G	A	PRTG	0.586704	1.01607	51441	0.378595	0.0061634	3773.21	0	821.228	intron_variant	*PRTG*	0.1064	0.0517	0.03965	0.1059	0.0508	0.03714
rs6493814	15	55739473	G	A	PRTG	0.586816	1.01593	51441	0.378357	0.00616193	3770.25	0	820.585	downstream_gene_variant	*PRTG*	0.1064	0.0517	0.03965	0.1059	0.0508	0.03714
rs12442771	15	55739901	A	G	PRTG	0.587539	1.01385	51441	0.377665	0.00616222	3756.11	0	817.514	downstream_gene_variant	*PRTG*	0.1064	0.0517	0.03963	0.1059	0.0508	0.03713
rs11630141	15	55735815	G	T	PRTG	0.590201	1.01333	51441	0.376264	0.00615989	3731.13	0	812.089	downstream_gene_variant	*PRTG*	0.1037	0.0515	0.04399	0.1035	0.0506	0.04063
rs4774802	15	55708272	T	C	PRTG	0.585123	1.02093	51441	0.374863	0.00615005	3715.25	0	808.639	intron_variant	*PRTG*	0.1152	0.0521	0.02698	0.116	0.0512	0.02341
rs11853512	15	55736378	A	C	PRTG	0.592106	0.993561	51441	0.376061	0.00622609	3648.25	0	794.087	downstream_gene_variant	*PRTG*	0.1037	0.0515	0.04397	0.1035	0.0506	0.04062
rs687128	15	55692407	A	C	PRTG	0.588685	1.01005	51441	0.370201	0.00614284	3631.92	0	790.54	intron_variant	*PRTG*	0.105	0.0521	0.04387	0.1041	0.0512	0.04204
rs12907892	15	55710821	T	C	PRTG	0.58634	1.02724	51441	0.366634	0.00618224	3517.01	0	765.581	intron_variant	*PRTG*	0.0868	0.0521	0.0955	0.0853	0.0512	0.09594
rs12901963	15	55728808	T	C	PRTG	0.58631	1.02658	51441	0.365362	0.00618715	3487.11	0	759.086	intron_variant	*PRTG*	0.0868	0.0521	0.0955	0.0853	0.0512	0.09594
rs12903239	15	55723569	T	C	PRTG	0.585544	1.02343	51441	0.36344	0.00616986	3469.89	0	755.344	intron_variant	*PRTG*	0.0791	0.0518	0.1266	0.0785	0.0509	0.1235
rs12912118	15	55730730	G	T	PRTG	0.584145	1.02618	51441	0.363497	0.0061806	3458.92	0	752.963	intron_variant	*PRTG*	0.0842	0.0519	0.1044	0.083	0.051	0.1039
rs11852746	15	55692067	T	C	PRTG	0.582048	1.02128	51441	0.362615	0.006175	3448.41	0	750.68	intron_variant	*PRTG*	0.0879	0.0523	0.09274	0.0856	0.0514	0.09606
rs7166952	15	55654184	C	A	PRTG	0.720816	1.00681	51441	0.390935	0.00670242	3402.09	0	740.618	downstream_gene_variant	*PRTG*	0.1506	0.0568	0.007969	0.1389	0.056	0.01306
rs7171834	15	55654420	C	T	PRTG	0.721414	1.00694	51441	0.389785	0.00670718	3377.31	0	735.235	downstream_gene_variant	*PRTG*	0.1426	0.0567	0.01196	0.1309	0.0559	0.01922
rs7171522	15	55654389	A	G	PRTG	0.7214	1.00715	51441	0.389731	0.00670637	3377.2	0	735.211	downstream_gene_variant	*PRTG*	0.1426	0.0567	0.01195	0.1309	0.0559	0.01922
rs7171318	15	55654268	A	C	PRTG	0.721471	1.00736	51441	0.389581	0.00670587	3375.09	0	734.754	downstream_gene_variant	*PRTG*	0.1426	0.0567	0.01195	0.1309	0.0559	0.01922
rs7165778	15	55653620	A	T	PRTG	0.72151	1.00692	51441	0.389378	0.00670762	3369.81	0	733.606	downstream_gene_variant	*PRTG*	0.1426	0.0567	0.01195	0.1309	0.0559	0.01922
rs7178379	15	55655354	G	A	PRTG	0.721896	1.00622	51441	0.388425	0.00671082	3350.14	0	729.335	3_prime_UTR_variant	*PRTG*	0.1426	0.0567	0.01196	0.1309	0.0559	0.01922
rs12438177	15	55659234	A	G	PRTG	0.619533	1.00743	51441	0.360288	0.0062247	3350.14	0	729.335	intron_variant	*PRTG*	0.0822	0.0524	0.1164	0.0763	0.0515	0.1387
rs11632762	15	55743123	G	A	PRTG	0.499491	1.03122	51441	0.348797	0.00612129	3246.84	0	706.895	upstream_gene_variant	*PRTG*	-0.0361	0.051	0.4786			
rs12903822	15	55666219	T	C	PRTG	0.608109	0.99318	51441	0.350035	0.00618389	3204.04	0	697.6	intron_variant	*PRTG*	0.0938	0.0526	0.07442	0.0873	0.0516	0.09069
rs11639086	15	55743484	T	A	PRTG	0.498786	1.03227	51441	0.345635	0.00611817	3191.48	0	694.871	upstream_gene_variant	*PRTG*	-0.0424	0.051	0.4063			
rs12440103	15	55743870	A	G	PRTG	0.498695	1.03226	51441	0.345465	0.00611846	3188.03	0	694.122	upstream_gene_variant	*PRTG*	-0.0429	0.051	0.4006			
rs66519039	15	55744616	A	G	PRTG	0.498579	1.03227	51441	0.345435	0.00611826	3187.69	0	694.047	upstream_gene_variant	*PRTG*	-0.0429	0.051	0.4006			
rs10152116	15	55740321	G	A	PRTG	0.496538	1.03164	51441	0.344184	0.00611504	3167.98	0	689.767	downstream_gene_variant	*PRTG*	-0.0428	0.0509	0.4005			
rs4774800	15	55658743	C	T	PRTG	0.728071	1.00502	51441	0.380458	0.00676796	3160.09	0	688.052	intron_variant	*PRTG*	0.1421	0.0566	0.01211	0.1314	0.0558	0.01856
rs11857254	15	55739042	C	T	PRTG	0.496947	1.02995	51441	0.342073	0.00610536	3139.17	0	683.509	downstream_gene_variant	*PRTG*	-0.0427	0.0508	0.4005			
rs12899976	15	55692241	A	C	PRTG	0.495692	1.0296	51441	0.342492	0.00611913	3132.72	0	682.109	intron_variant	*PRTG*	-0.0521	0.0516	0.3124			

rs12908813	15	55706050	C	T	PRTG	0.492895	1.02614	51441	0.34313	0.00613082	3132.43	0	682.044	intron_variant	*PRTG*	-0.0545	0.051	0.2855			
rs9920246	15	55653255	A	G	PRTG	0.728255	1.00373	51441	0.379076	0.00677594	3129.78	0	681.469	downstream_gene_variant	*PRTG*	0.132	0.0566	0.01967	0.1225	0.0558	0.02803
rs10851590	15	55697741	C	T	PRTG	0.492822	1.03	51441	0.341912	0.00611438	3126.98	0	680.861	intron_variant	*PRTG*	-0.0513	0.0512	0.3172			
rs12915423	15	55728117	C	T	PRTG	0.49701	1.02802	51441	0.341092	0.0061045	3122.06	0	679.793	intron_variant	*PRTG*	-0.0493	0.0514	0.3377			
rs4392003	15	55667220	A	C	PRTG	0.586945	0.956812	51441	0.348713	0.00624585	3117.12	0	678.719	intron_variant	*PRTG*	0.0917	0.0525	0.08068	0.0837	0.0515	0.1046
rs3985768	15	55705346	A	T	PRTG	0.49299	1.0328	51441	0.340858	0.00610722	3115.02	0	678.263	intron_variant	*PRTG*	-0.0495	0.0512	0.3337			
rs12914863	15	55743537	A	G	PRTG	0.500905	1.02568	51441	0.339784	0.0060958	3107.02	0	676.525	upstream_gene_variant	*PRTG*	-0.0424	0.051	0.4065			
rs4774222	15	55738558	T	C	PRTG	0.49339	1.02874	51441	0.335184	0.00609398	3025.27	0	658.767	downstream_gene_variant	*PRTG*	-0.0391	0.0505	0.4384			
rs9920546	15	55654534	C	T	PRTG	0.610751	1.04106	51441	0.343412	0.00630832	2963.49	0	645.348	downstream_gene_variant	*PRTG*	0.1117	0.0521	0.03205	0.1073	0.0512	0.03606
rs11855737	15	55691589	A	G	PRTG	0.520357	1.01678	51441	0.331897	0.00617294	2890.83	0	629.565	intron_variant	*PRTG*	0.0921	0.0523	0.07818	0.0846	0.0514	0.09992
rs11071204	15	55738828	A	G	PRTG	0.486749	1.02277	51441	-0.323134	0.00608025	2824.37	0	615.128	downstream_gene_variant	*PRTG*	0.0571	0.0509	0.2621	0.0608	0.05	0.2241
rs73423335	15	55669174	A	T	PRTG	0.116322	1.00326	51441	-0.491473	0.00936755	2752.62	0	599.543	intron_variant	*PRTG*	0.2479	0.0784	0.001561	0.2441	0.0767	0.00147
rs73423329	15	55666266	C	T	PRTG	0.112228	1.00317	51441	-0.49662	0.00950495	2729.91	0	594.609	intron_variant	*PRTG*	0.2369	0.0788	0.002623	0.2318	0.0771	0.002656
rs73408312	15	55670101	T	C	PRTG	0.111649	1.00392	51441	-0.496956	0.00952627	2721.38	0	592.756	intron_variant	*PRTG*	0.2453	0.0785	0.001779	0.2403	0.0769	0.001775
rs79523867	15	55657041	T	C	PRTG	0.110925	1.00621	51441	-0.497866	0.00955041	2717.57	0	591.929	intron_variant	*PRTG*	0.231	0.0787	0.003336	0.2261	0.0771	0.003356
rs73408332	15	55684762	C	T	PRTG	0.111547	1.00358	51441	-0.496823	0.0095325	2716.37	0	591.668	upstream_gene_variant	*PRTG*	0.2391	0.0788	0.002413	0.2362	0.0772	0.002205
rs56233400	15	55660254	T	A	PRTG	0.110885	1.00588	51441	-0.497822	0.00955416	2714.96	0	591.361	intron_variant	*PRTG*	0.2358	0.0789	0.002818	0.2309	0.0773	0.002814
rs76123597	15	55662309	T	C	PRTG	0.110977	1.00575	51441	-0.497641	0.00955116	2714.69	0	591.302	intron_variant	*PRTG*	0.2453	0.0785	0.001779	0.2403	0.0769	0.001775
rs79231895	15	55654817	G	A	PRTG	0.110489	1.00684	51441	-0.497808	0.00956857	2706.64	0	589.553	downstream_gene_variant	*PRTG*	0.2358	0.0789	0.002818	0.2309	0.0773	0.002814
rs75013819	15	55662498	C	G	PRTG	0.112354	1.00604	51441	-0.493573	0.00949966	2699.53	0	588.009	intron_variant	*PRTG*	0.2479	0.0784	0.001561	0.2441	0.0767	0.00147
rs4337238	15	55664847	T	A	PRTG	0.881816	1.00316	51441	0.483539	0.00932351	2689.71	0	585.875	intron_variant	*PRTG*	0.2451	0.0782	0.001727	0.2412	0.0766	0.001644
rs2053335	15	55664672	G	A	PRTG	0.881153	1.00459	51441	0.482288	0.00930938	2683.94	0	584.622	intron_variant	*PRTG*	0.2451	0.0782	0.001727	0.2412	0.0766	0.001644
rs76917877	15	55651001	C	G	PRTG	0.111227	1.00132	51441	-0.484123	0.00956857	2559.87	0	557.671	downstream_gene_variant	*PRTG*	0.231	0.0787	0.003336	0.2261	0.0771	0.003356
rs75996169	15	55650084	T	G	PRTG	0.112581	1.0015	51441	-0.480243	0.00951842	2545.62	0	554.575	downstream_gene_variant	*PRTG*	0.2394	0.0784	0.002274	0.2356	0.0768	0.00217
rs147191955	15	55646480	G	A	PRTG	0.112413	0.997889	51441	-0.4795	0.00954168	2525.39	0	550.18	intron_variant	*PRTG*	0.2443	0.0786	0.001897	0.2403	0.077	0.00181
rs75577582	15	55696861	T	C	PRTG	0.111698	1.00381	51441	-0.473406	0.00953195	2466.63	0	537.417	intron_variant	*PRTG*	0.2163	0.0783	0.005697	0.2131	0.0766	0.005425
rs79231255	15	55699759	G	A	PRTG	0.111084	1.00495	51441	-0.474453	0.00955715	2464.51	0	536.955	intron_variant	*PRTG*	0.2164	0.0782	0.005689	0.2131	0.0766	0.005419
rs16976479	15	55696340	A	G	PRTG	0.110775	1.00535	51441	-0.475116	0.00957197	2463.75	0	536.79	intron_variant	*PRTG*	0.2163	0.0783	0.005697	0.2131	0.0766	0.005425
rs74406062	15	55699976	T	G	PRTG	0.110671	1.00602	51441	-0.47499	0.0095746	2461.09	0	536.213	intron_variant	*PRTG*	0.2163	0.0783	0.005697	0.2131	0.0766	0.005425
rs4412917	15	55671646	G	A	PRTG	0.454128	1.0078	51441	0.305977	0.0061868	2445.94	0	532.921	intron_variant	*PRTG*	-0.0604	0.0517	0.2428	-0.0532	0.0509	0.2961
rs112595988	15	55630389	C	T	PRTG	0.110155	1.00232	51441	-0.475207	0.00961329	2443.56	0	532.403	intron_variant	*PRTG*	0.236	0.0789	0.00277	0.2343	0.0773	0.002427
rs142697499	15	55627592	G	A	PRTG	0.110711	1.00313	51441	-0.473824	0.00958832	2442.02	0	532.069	intron_variant	*PRTG*	0.236	0.0789	0.00277	0.2343	0.0773	0.002427
rs16976434	15	55627851	T	C	PRTG	0.11015	1.0034	51441	-0.474347	0.00960823	2437.28	0	531.041	intron_variant	*PRTG*	0.236	0.0789	0.002774	0.2342	0.0773	0.002429
rs113032684	15	55634225	G	A	PRTG	0.11149	1.00034	51441	-0.471975	0.00957344	2430.54	0	529.576	intron_variant	*PRTG*	0.2387	0.0788	0.002448	0.2382	0.0771	0.002008
rs76613233	15	55601008	G	A	PRTG	0.112004	1.0021	51441	-0.469136	0.00953889	2418.81	0	527.027	intergenic_region	*PYGO1-PRTG*	0.2354	0.0789	0.00284	0.2344	0.0772	0.002405
rs11853232	15	55623435	C	T	PRTG	0.111404	1.00432	51441	-0.469146	0.00955757	2409.47	0	524.999	intron_variant	*PRTG*	0.2387	0.0788	0.002448	0.2382	0.0771	0.002008
rs76674695	15	55622150	T	C	PRTG	0.11142	1.00463	51441	-0.468864	0.00955551	2407.61	0	524.595	intron_variant	*PRTG*	0.2387	0.0788	0.002451	0.2382	0.0771	0.00201
rs111509740	15	55621605	C	T	PRTG	0.111427	1.00483	51441	-0.468743	0.00955434	2406.96	0	524.453	intron_variant	*PRTG*	0.2286	0.0789	0.003757	0.2286	0.0772	0.003068
rs36016255	15	55621344	C	T	PRTG	0.111428	1.00483	51441	-0.468639	0.00955431	2405.9	0	524.224	intron_variant	*PRTG*	0.2387	0.0788	0.002448	0.2382	0.0771	0.002008
rs111493698	15	55607856	C	T	PRTG	0.111503	1.00289	51441	-0.468035	0.00956065	2396.53	0	522.187	downstream_gene_variant	*PRTG*	0.2286	0.079	0.003821	0.2277	0.0774	0.003255
rs78823862	15	55609285	T	C	PRTG	0.111559	1.00244	51441	-0.468026	0.00956067	2396.42	0	522.165	downstream_gene_variant	*PRTG*	0.2286	0.079	0.003825	0.2277	0.0774	0.003258
rs79482995	15	55608504	G	A	PRTG	0.111855	1.00119	51441	-0.467735	0.00955552	2396.02	0	522.077	downstream_gene_variant	*PRTG*	0.2286	0.079	0.003821	0.2277	0.0774	0.003255
rs1438914	15	55620677	T	A	PRTG	0.111477	1.00564	51441	-0.467137	0.00954892	2393.21	0	521.466	missense_variant	*PRTG*	0.2387	0.0788	0.002448	0.2382	0.0771	0.002008
rs112470651	15	55605744	G	C	PRTG	0.111791	1.00308	51441	-0.466861	0.009549	2390.33	0	520.842	intergenic_region	*PYGO1-PRTG*	0.2286	0.079	0.003821	0.2277	0.0774	0.003255

rs3988162	15	55596410	C	T	PRTG	0.112782	1.00772	51441	-0.462938	0.0094898	2379.75	0	518.542	intergenic_region	*PYGO1-PRTG*	0.2301	0.0786	0.003409	0.2308	0.077	0.002718
rs78081173	15	55603175	T	C	PRTG	0.114211	0.998226	51441	-0.461356	0.00946139	2377.73	0	518.103	intergenic_region	*PYGO1-PRTG*	0.2316	0.0786	0.003199	0.2324	0.0769	0.002514
rs720508	15	55638337	G	A	PRTG	0.106008	0.976391	51441	-0.482963	0.00990658	2376.73	0	517.887	intron_variant	*PRTG*	0.2473	0.0792	0.001791	0.2441	0.0775	0.001637
rs56132020	15	55602246	G	A	PRTG	0.112173	1.00348	51441	-0.464417	0.00953296	2373.35	0	517.151	intergenic_region	*PYGO1-PRTG*	0.2354	0.0789	0.00284	0.2344	0.0772	0.002405
rs617137	15	55612046	T	C	PRTG	0.884085	0.997129	51441	0.457446	0.00940267	2366.88	0	515.747	3_prime_UTR_variant	*PRTG*	0.2315	0.0788	0.0033	0.232	0.0771	0.002636
rs78504519	15	55596586	C	A	PRTG	0.112614	1.0081	51441	-0.461435	0.009494	2362.23	0	514.736	intergenic_region	*PYGO1-PRTG*	0.2301	0.0786	0.003409	0.2308	0.077	0.002718
rs74513809	15	55594205	G	C	PRTG	0.110922	0.990715	51441	-0.467259	0.00964	2349.43	0	511.955	intergenic_region	*PYGO1-PRTG*	0.2301	0.0786	0.003409	0.2308	0.077	0.002718
rs76105455	15	55594213	G	A	PRTG	0.110914	0.991096	51441	-0.467065	0.0096384	2348.26	0	511.701	intergenic_region	*PYGO1-PRTG*	0.2301	0.0786	0.003409	0.2308	0.077	0.002718
rs112282565	15	55621138	G	A	PRTG	0.108366	1.00178	51441	-0.469214	0.00968585	2346.75	0	511.374	intron_variant	*PRTG*	0.2056	0.081	0.01117	0.2061	0.0793	0.009362
rs11071200	15	55657884	C	A	PRTG	0.217773	0.999182	51441	-0.353259	0.00729736	2343.45	0	510.657	intron_variant	*PRTG*	0.0979	0.0625	0.1173	0.1032	0.0612	0.09191
rs77360292	15	55713497	A	G	PRTG	0.102908	1.00649	51441	-0.471137	0.00986965	2278.72	0	496.595	intron_variant	*PRTG*	0.2744	0.0813	0.000737	0.2737	0.0796	0.000583
rs999303	15	55716227	T	C	PRTG	0.103146	1.00539	51441	-0.470657	0.00986197	2277.62	0	496.356	intron_variant	*PRTG*	0.2744	0.0813	0.000737	0.2737	0.0796	0.000583
rs79539321	15	55728821	T	C	PRTG	0.10298	1.00438	51441	-0.471258	0.00987515	2277.35	0	496.297	intron_variant	*PRTG*	0.2744	0.0813	0.000737	0.2737	0.0796	0.000583
rs77837244	15	55710621	C	T	PRTG	0.102698	1.00669	51441	-0.471319	0.00987907	2276.14	0	496.033	intron_variant	*PRTG*	0.2744	0.0813	0.000735	0.2737	0.0796	0.0005821
rs75576399	15	55714297	T	C	PRTG	0.102736	1.00684	51441	-0.471114	0.0098768	2275.2	0	495.829	intron_variant	*PRTG*	0.2744	0.0813	0.000737	0.2737	0.0796	0.000583
rs56190205	15	55721279	C	A	PRTG	0.102584	1.00546	51441	-0.471658	0.00989199	2273.46	0	495.452	intron_variant	*PRTG*	0.2744	0.0813	0.000735	0.2737	0.0796	0.0005821
rs146981285	15	55727752	C	T	PRTG	0.102174	1.00648	51441	-0.471992	0.00991063	2268.12	0	494.292	intron_variant	*PRTG*	0.2744	0.0813	0.000735	0.2737	0.0796	0.0005821
rs2414428	15	55715497	T	C	PRTG	0.102859	1.00636	51441	-0.470141	0.00987333	2267.4	0	494.136	intron_variant	*PRTG*	0.2744	0.0813	0.000737	0.2737	0.0796	0.000583
rs112965090	15	55603340	G	C	PRTG	0.118041	0.9943	51441	-0.444691	0.00935067	2261.68	0	492.894	intergenic_region	*PYGO1-PRTG*	0.2266	0.0781	0.003722	0.223	0.0765	0.003553
rs74365454	15	55703940	G	A	PRTG	0.104343	1.00764	51441	-0.461317	0.00981402	2209.56	0	481.569	intron_variant	*PRTG*	0.2538	0.0808	0.001676	0.2554	0.0791	0.001236
rs117572622	15	55701607	C	T	PRTG	0.104792	1.00617	51441	-0.460452	0.00979581	2209.47	0	481.55	intron_variant	*PRTG*	0.2538	0.0808	0.001676	0.2554	0.0791	0.001236
rs116360825	15	55713728	T	C	PRTG	0.103237	0.977005	51441	-0.470159	0.010006	2207.83	0	481.193	intron_variant	*PRTG*	0.2744	0.0813	0.000737	0.2737	0.0796	0.000583
rs56220110	15	55722984	G	A	PRTG	0.104866	1.00468	51441	-0.460364	0.00979809	2207.6	0	481.144	intron_variant	*PRTG*	0.2538	0.0808	0.001676	0.2554	0.0791	0.001236
rs147782215	15	55730751	G	C	PRTG	0.105	1.00403	51441	-0.460086	0.00979355	2206.98	0	481.009	intron_variant	*PRTG*	0.2538	0.0808	0.001676	0.2554	0.0791	0.001236
rs76472232	15	55700464	T	G	PRTG	0.104373	1.00744	51441	-0.46104	0.00981401	2206.91	0	480.994	intron_variant	*PRTG*	0.2538	0.0808	0.001679	0.2554	0.0791	0.001238
rs75761038	15	55731711	C	T	PRTG	0.104823	1.00482	51441	-0.460178	0.00979896	2205.43	0	480.672	intron_variant	*PRTG*	0.2538	0.0808	0.001676	0.2554	0.0791	0.001236
rs76319732	15	55722183	C	T	PRTG	0.104753	1.00511	51441	-0.460311	0.00980227	2205.21	0	480.625	intron_variant	*PRTG*	0.2538	0.0808	0.001676	0.2554	0.0791	0.001236
rs77600232	15	55729943	G	A	PRTG	0.104818	1.00486	51441	-0.460116	0.00979886	2204.87	0	480.551	intron_variant	*PRTG*	0.2538	0.0808	0.001676	0.2554	0.0791	0.001236
rs78993121	15	55729908	G	A	PRTG	0.104816	1.00498	51441	-0.459936	0.0097984	2203.35	0	480.221	intron_variant	*PRTG*	0.2538	0.0808	0.001676	0.2554	0.0791	0.001236
rs80329464	15	55736444	G	T	PRTG	0.104977	1.00422	51441	-0.459395	0.00979535	2199.54	0	479.393	downstream_gene_variant	*PRTG*	0.2488	0.0799	0.001852	0.2505	0.0782	0.001352
rs76925378	15	55728975	T	C	PRTG	0.104379	1.00603	51441	-0.460453	0.00981834	2199.35	0	479.353	intron_variant	*PRTG*	0.2538	0.0808	0.001679	0.2554	0.0791	0.001238
rs77995643	15	55736341	C	G	PRTG	0.104907	1.0039	51441	-0.459594	0.00980043	2199.17	0	479.312	downstream_gene_variant	*PRTG*	0.2488	0.0799	0.001855	0.2505	0.0782	0.001353
rs79234996	15	55734930	A	G	PRTG	0.104397	1.00673	51441	-0.459873	0.00981652	2194.62	0	478.325	downstream_gene_variant	*PRTG*	0.2538	0.0808	0.001679	0.2554	0.0791	0.001238
rs8030790	15	55675199	A	G	PRTG	0.211361	1.00429	51441	-0.32978	0.00735736	2009.11	0	438.022	downstream_gene_variant	*PRTG*	0.1011	0.0634	0.1105	0.1043	0.0621	0.09304
rs1438915	15	55620330	C	T	PRTG	0.0810906	0.984556	51441	-0.468012	0.0111206	1771.16	0	386.325	intron_variant	*PRTG*	0.2618	0.0884	0.003055	0.253	0.0866	0.003489
rs117702444	15	55666531	T	G	PRTG	0.0444601	0.976675	51441	0.587849	0.0147475	1588.9	0	346.724	intron_variant	*PRTG*	-0.1497	0.1121	0.1817	-0.1145	0.1097	0.2967
rs492363	15	55651344	A	G	PRTG	0.77142	1.00088	51441	0.284684	0.00720728	1560.21	0	340.489	downstream_gene_variant	*PRTG*	0.0808	0.062	0.1924	0.0808	0.0606	0.1826
rs8025445	15	55674444	C	A	PRTG	0.31801	1.01723	51441	-0.253948	0.00652611	1514.19	0	330.49	downstream_gene_variant	*PRTG*	0.0638	0.0563	0.2568	0.0742	0.0552	0.179
rs1550330	15	55692461	C	T	PRTG	0.778789	1.01599	51441	0.279779	0.00731802	1461.65	0	319.074	intron_variant	*PRTG*	0.0069	0.0637	0.9134	-0.0029	0.0626	0.9635
rs7181496	15	55594770	A	G	PRTG	0.225496	0.989309	51441	-0.26921	0.0072325	1385.5	2.97E-303	302.527	intergenic_region	*PYGO1-PRTG*	0.0962	0.0624	0.1232	0.0939	0.0612	0.1249
rs4774797	15	55650374	G	C	PRTG	0.791154	0.995892	51441	0.270625	0.00741118	1333.4	6.24E-292	291.205	downstream_gene_variant	*PRTG*	0.0817	0.0632	0.1959			
rs8031192	15	55696882	G	A	PRTG	0.789204	1.02068	51441	0.272188	0.00746935	1327.93	9.66E-291	290.015	intron_variant	*PRTG*	0.0141	0.0646	0.827	0.0067	0.0635	0.916
rs581287	15	55632411	T	C	PRTG	0.680661	1.01739	51441	0.236004	0.00656326	1293	3.75E-283	282.426	intron_variant	*PRTG*	0.1199	0.0554	0.03027	0.1256	0.0543	0.02061
rs558290	15	55635500	T	C	PRTG	0.673519	1.01801	51441	0.227788	0.00651794	1221.35	1.4E-267	266.855	intron_variant	*PRTG*	0.0979	0.0553	0.07674	0.1051	0.0542	0.05277

rs28512199	15	55642315	G	C	PRTG	0.210314	0.996888	51441	-0.257152	0.00741069	1204.1	7.85E-264	263.105	intron_variant	*PRTG*	0.1164	0.0643	0.07024	0.1159	0.063	0.06596
rs489672	15	55638379	A	T	PRTG	0.3252	1.01317	51441	-0.226888	0.00654243	1202.66	1.61E-263	262.793	intron_variant	*PRTG*	0.0995	0.0555	0.07299	0.1059	0.0544	0.05148
rs720509	15	55638344	C	T	PRTG	0.323081	1.01068	51441	-0.226731	0.00656402	1193.12	1.91E-261	260.719	intron_variant	*PRTG*	0.0977	0.0554	0.07805	0.1035	0.0544	0.05685
rs16976436	15	55631757	C	T	PRTG	0.208507	1.00063	51441	-0.253097	0.00741785	1164.17	3.73E-255	254.428	intron_variant	*PRTG*	0.1108	0.0644	0.08514	0.1106	0.0631	0.07957
rs7182117	15	55747685	G	C	PRTG	0.179985	1.01656	51441	-0.267737	0.00786904	1157.64	9.82E-254	253.008	upstream_gene_variant	*PRTG*	0.0238	0.0686	0.7291	0.0166	0.0676	0.8064
rs4332687	15	55747592	C	T	PRTG	0.180307	1.01652	51441	-0.267359	0.00786322	1156.08	2.14E-253	252.669	upstream_gene_variant	*PRTG*	0.0238	0.0686	0.7291	0.0166	0.0676	0.8064
rs370988976	15	55745528	A	G	PRTG	0.061667	0.976411	51441	-0.422846	0.0126684	1114.09	2.86E-244	243.544	upstream_gene_variant	*PRTG*	0.3223	0.1013	0.00147	0.3126	0.0994	0.001656
rs77118243	15	55740674	G	A	PRTG	0.0596877	0.956273	51441	-0.431565	0.0129951	1102.89	7.76E-242	241.11	synonymous_variant	*PRTG*	0.3197	0.1009	0.001536	0.3099	0.099	0.001744
rs552292	15	55596834	A	T	PRTG	0.660276	0.990027	51441	0.211553	0.00638659	1097.24	1.32E-240	239.881	intergenic_region	*PYGO1-PRTG*	0.0725	0.052	0.1633	0.0738	0.0511	0.1487
rs7176818	15	55646292	A	G	PRTG	0.610201	0.969883	51441	0.215218	0.00650859	1093.41	8.91E-240	239.05	intron_variant	*PRTG*	0.0528	0.0529	0.3178	0.0421	0.0519	0.4176
rs12591246	15	55732069	T	C	PRTG	0.184919	1.01181	51441	-0.256219	0.00779513	1080.38	6.08E-237	236.216	intron_variant	*PRTG*	-0.0175	0.0681	0.7976	-0.0285	0.0671	0.671
rs2414427	15	55713593	T	A	PRTG	0.184196	1.01229	51441	-0.255401	0.00779049	1074.77	1E-235	234.998	intron_variant	*PRTG*	-0.0152	0.068	0.8236	-0.0269	0.067	0.6886
rs12594642	15	55702160	T	C	PRTG	0.182348	1.01732	51441	-0.248477	0.00783627	1005.43	1.18E-220	219.927	intron_variant	*PRTG*	-0.029	0.068	0.6701	-0.0401	0.067	0.5494
rs1371056	15	55702711	T	G	PRTG	0.182497	1.01658	51441	-0.248269	0.00783652	1003.69	2.84E-220	219.547	intron_variant	*PRTG*	-0.029	0.068	0.6701	-0.0401	0.067	0.5494
rs12592293	15	55747471	G	A	PRTG	0.170555	1.01852	51441	-0.251244	0.00805436	973.034	1.31E-213	212.884	upstream_gene_variant	*PRTG*	0.0174	0.069	0.801	0.0114	0.068	0.867
rs2414425	15	55709401	C	T	PRTG	0.178447	1.01211	51441	-0.238007	0.00789282	909.315	9.27E-200	199.033	intron_variant	*PRTG*	0.0007	0.0689	0.9918	-0.0073	0.0679	0.9143
rs12914679	15	55603029	T	A	PRTG	0.515653	1.0094	51441	-0.183645	0.00610202	905.754	5.51E-199	198.259	intergenic_region	*PYGO1-PRTG*	-0.0719	0.0501	0.1512	-0.0818	0.0493	0.09736
rs715321	15	55717450	A	T	PRTG	0.174735	1.01536	51441	-0.238756	0.00796557	898.413	2.17E-197	196.663	intron_variant	*PRTG*	-0.0195	0.0685	0.7753	-0.0309	0.0674	0.6466
rs8027131	15	55645164	G	A	PRTG	0.469858	0.999686	51441	0.182675	0.0061219	890.396	1.2E-195	194.92	intron_variant	*PRTG*	-0.0576	0.0514	0.262	-0.0624	0.0505	0.2164
rs7180112	15	55735111	T	C	PRTG	0.164158	1.01571	51441	-0.243806	0.00817114	890.27	1.28E-195	194.893	downstream_gene_variant	*PRTG*	0.0101	0.0713	0.887	-0.0053	0.0704	0.9394
rs77957184	15	55625768	C	T	PRTG	0.473123	1.01865	51441	0.1817	0.00611168	883.872	3.15E-194	193.502	intron_variant	*PRTG*	-0.0775	0.0511	0.1294	-0.0832	0.0502	0.09745
rs191781137	15	55642101	G	A	PRTG	0.0384361	0.855901	51441	-0.498905	0.0169022	871.26	1.74E-191	190.76	intron_variant	*PRTG*	0.3335	0.1914	0.08147	0.3056	0.1882	0.1045
rs654844	15	55626716	C	G	PRTG	0.477665	1.01047	51441	0.17946	0.00608531	869.697	3.79E-191	190.421	intron_variant	*PRTG*	-0.0769	0.0508	0.1303	-0.083	0.05	0.09668
rs4774217	15	55601113	C	A	PRTG	0.467045	1.00811	51441	0.179747	0.00611707	863.452	8.65E-190	189.063	intergenic_region	*PYGO1-PRTG*	-0.0504	0.0506	0.32	-0.0562	0.0498	0.2598
rs2579033	15	55623452	G	A	PRTG	0.473062	1.01243	51441	0.178698	0.00608611	862.107	1.7E-189	188.77	intron_variant	*PRTG*	-0.0769	0.0508	0.1299	-0.0811	0.0499	0.1039
rs4453409	15	55634735	G	A	PRTG	0.464732	1.01611	51441	0.179244	0.00611409	859.463	6.37E-189	188.196	intron_variant	*PRTG*	-0.0503	0.0513	0.3264	-0.056	0.0504	0.2667
rs1550326	15	55641001	G	T	PRTG	0.423087	1.00859	51441	-0.179824	0.00614203	857.182	2E-188	187.7	intron_variant	*PRTG*	0.0502	0.051	0.3252	0.0524	0.0501	0.2955
rs546918	15	55607918	G	A	PRTG	0.475729	1.00718	51441	0.178265	0.00609547	855.303	5.12E-188	187.291	downstream_gene_variant	*PRTG*	-0.0625	0.0509	0.2187	-0.0698	0.05	0.1622
rs1659296	15	55640791	C	G	PRTG	0.46828	1.00575	51441	0.178254	0.006103	853.08	1.56E-187	186.808	intron_variant	*PRTG*	-0.0574	0.0517	0.2665	-0.063	0.0508	0.2149
rs491014	15	55616005	T	C	PRTG	0.486703	1.00858	51441	0.17786	0.00609546	851.419	3.57E-187	186.447	3_prime_UTR_variant	*PRTG*	-0.0762	0.0508	0.1334	-0.0874	0.0499	0.07973
rs530798	15	55612529	G	A	PRTG	0.486451	1.0078	51441	0.177422	0.00609721	846.748	3.7E-186	185.432	3_prime_UTR_variant	*PRTG*	-0.0705	0.0505	0.1628	-0.0806	0.0496	0.1044
rs74017540	15	55612747	T	C	PRTG	0.472618	1.01466	51441	0.177672	0.00614893	834.907	1.39E-183	182.857	3_prime_UTR_variant	*PRTG*	-0.0561	0.0511	0.2718	-0.0651	0.0502	0.1946
rs4561398	15	55640419	T	C	PRTG	0.521427	0.997051	51441	-0.174674	0.00606429	829.654	1.93E-182	181.715	intron_variant	*PRTG*	-0.0595	0.0516	0.2487	-0.0661	0.0507	0.1921
rs62017965	15	55626515	G	C	PRTG	0.465684	1.01882	51441	0.176092	0.00611815	828.401	3.61E-182	181.443	intron_variant	*PRTG*	-0.0591	0.0516	0.2516	-0.0652	0.0507	0.1982
rs488986	15	55606791	G	T	PRTG	0.483903	0.997003	51441	0.174532	0.0060644	828.276	3.85E-182	181.415	downstream_gene_variant	*PRTG*	-0.0679	0.051	0.1828	-0.0757	0.0501	0.1312
rs1659295	15	55598880	A	G	PRTG	0.536046	1.00042	51441	-0.173039	0.00609635	805.651	3.18E-177	176.497	intergenic_region	*PYGO1-PRTG*	-0.0494	0.0505	0.3281	-0.0537	0.0497	0.2802
rs12438007	15	55600766	C	T	PRTG	0.481104	1.01169	51441	0.173803	0.00614871	799.003	8.89E-176	175.051	intergenic_region	*PYGO1-PRTG*	-0.0582	0.0511	0.2545	-0.067	0.0502	0.1824
rs7181481	15	55594746	A	G	PRTG	0.434661	0.997641	51441	-0.172645	0.00610826	798.868	9.51E-176	175.022	intergenic_region	*PYGO1-PRTG*	0.0369	0.0504	0.4642	0.0367	0.0496	0.4584
rs62043872	15	55708361	C	T	PRTG	0.171286	1.01319	51441	-0.223933	0.00802504	778.649	2.37E-171	170.626	intron_variant	*PRTG*	-0.006	0.0693	0.9305	-0.0129	0.0683	0.8502
rs2414424	15	55707865	C	T	PRTG	0.17126	1.01362	51441	-0.223646	0.00802372	776.909	5.65E-171	170.248	intron_variant	*PRTG*	-0.006	0.0693	0.9305	-0.0129	0.0683	0.8502
rs12592192	15	55730126	G	A	PRTG	0.156771	1.01398	51441	-0.229776	0.00832564	761.683	1.16E-167	166.937	intron_variant	*PRTG*	0.0047	0.0717	0.9473	-0.0092	0.0708	0.8963
rs2414426	15	55713534	C	G	PRTG	0.168623	1.01652	51441	-0.220216	0.00808039	742.73	1.53E-163	162.816	intron_variant	*PRTG*	-0.006	0.0694	0.9307	-0.0129	0.0683	0.8504
rs9920262	15	55653412	C	A	PRTG	0.831059	1.02365	51441	0.210635	0.00806101	682.78	1.66E-150	149.78	downstream_gene_variant	*PRTG*	0.0335	0.0711	0.6373	0.0171	0.0701	0.807
rs10851589	15	55657551	C	T	PRTG	0.832069	1.02141	51441	0.209819	0.00808695	673.164	2.05E-148	147.688	intron_variant	*PRTG*	0.0335	0.0711	0.6373	0.0171	0.0701	0.807

rs9920076	15	55653183	T	A	PRTG	0.831524	1.02197	51441	0.208954	0.008077	669.269	1.44E-147	146.841	downstream_gene_variant	*PRTG*	0.0247	0.0711	0.7282	0.0091	0.0701	0.8971
rs550226	15	55682548	A	T	PRTG	0.16134	0.992278	51441	-0.209316	0.00828739	637.923	9.46E-141	140.024	upstream_gene_variant	*PRTG*	0.0205	0.0709	0.7722	0.0028	0.07	0.9681
rs62043869	15	55673773	T	C	PRTG	0.159038	1.0193	51441	-0.208322	0.00825937	636.172	2.27E-140	139.644	downstream_gene_variant	*PRTG*	0.042	0.0712	0.555	0.0264	0.0702	0.7067
rs572531	15	55652319	G	A	PRTG	0.162844	1.01467	51441	-0.206206	0.00818147	635.242	3.62E-140	139.441	downstream_gene_variant	*PRTG*	0.033	0.0713	0.6434	0.0182	0.0703	0.7952
rs10518817	15	55661402	A	T	PRTG	0.158878	1.02013	51441	-0.206564	0.00825705	625.833	4.03E-138	137.395	intron_variant	*PRTG*	0.035	0.0711	0.6224	0.0187	0.0702	0.7895
rs8030795	15	55745937	G	C	PRTG	0.287123	0.999989	51441	-0.164249	0.00664448	611.059	6.58E-135	134.182	upstream_gene_variant	*PRTG*	0.0595	0.0591	0.3143	0.0707	0.058	0.223
rs28556884	15	55673323	T	C	PRTG	0.253975	1.01129	51441	-0.169563	0.00690731	602.619	4.51E-133	132.346	downstream_gene_variant	*PRTG*	-0.0217	0.0603	0.7189	-0.0296	0.0593	0.6173
rs11639131	15	55627385	G	A	PRTG	0.413948	1.01625	51441	-0.150023	0.00614912	595.235	1.82E-131	130.74	intron_variant	*PRTG*	0.0479	0.0519	0.3563	0.0493	0.051	0.3343
rs1530087	15	55697831	G	A	PRTG	0.150002	1.01936	51441	-0.205429	0.00848563	586.079	1.79E-129	128.748	intron_variant	*PRTG*	0.0071	0.0728	0.922	-0.0022	0.0718	0.975
rs1986012	15	55691073	T	C	PRTG	0.251666	1.00952	51441	-0.165352	0.00697465	562.052	3.01E-124	123.522	intron_variant	*PRTG*	-0.033	0.0606	0.5857	-0.0456	0.0596	0.4444
rs4774799	15	55658511	C	T	PRTG	0.839739	1.02048	51441	0.190646	0.00824603	534.523	2.93E-118	117.533	intron_variant	*PRTG*	0.0269	0.0716	0.7074	0.0116	0.0706	0.87
rs6493805	15	55674043	T	G	PRTG	0.843352	1.01824	51441	0.191465	0.00830858	531.039	1.68E-117	116.775	downstream_gene_variant	*PRTG*	0.0312	0.0717	0.6637	0.0155	0.0708	0.8264
rs16976450	15	55668258	A	G	PRTG	0.176957	1.00855	51441	-0.178468	0.0079446	504.636	9.31E-112	111.031	intron_variant	*PRTG*	0.0481	0.0677	0.4779	0.0245	0.0668	0.714
rs4774220	15	55670663	G	A	PRTG	0.246689	1.01366	51441	-0.154744	0.00696714	493.311	2.71E-109	108.567	intron_variant	*PRTG*	-0.0251	0.0602	0.6767	-0.0307	0.0592	0.6047
rs11636555	15	55680453	A	G	PRTG	0.02108	0.904631	51441	0.485348	0.021988	487.233	5.7E-108	107.244	upstream_gene_variant	*PRTG*	0.0513	0.1612	0.7504	0.0749	0.1576	0.6344
rs934886	15	55647761	A	G	PRTG	0.637118	1.06214	51441	0.13335	0.00641909	431.556	7.452E-96	95.1277	intron_variant	*PRTG*	-0.0288	0.0546	0.5983	-0.0302	0.0536	0.5731
rs8034113	15	55663666	G	C	PRTG	0.825565	1.00664	51441	0.161126	0.00798729	406.943	1.697E-90	89.7704	intron_variant	*PRTG*	0.0382	0.068	0.5746	0.0145	0.067	0.8288
rs80351750	15	55644421	G	C	PRTG	0.011289	0.986488	51441	-0.524567	0.0287051	333.952	1.324E-74	73.878	intron_variant	*PRTG*	-0.0509	0.2425	0.8336	-0.0217	0.2375	0.9271
rs142626067	15	55604605	A	G	PRTG	0.0116725	0.88667	51441	-0.485526	0.029638	268.366	2.577E-60	59.5889	intergenic_region	*PYGO1-PRTG*	0.2083	0.1927	0.2798	0.2147	0.1883	0.2543
rs2576936	15	55718609	G	C	PRTG	0.0280692	0.988906	51441	-0.291591	0.0183614	252.196	8.626E-57	56.0642	intron_variant	*PRTG*	0.1766	0.1488	0.2355	0.1729	0.146	0.2364
rs1863756	15	55716802	T	C	PRTG	0.0334236	1.03483	51441	-0.273212	0.0173199	248.834	4.663E-56	55.3313	intron_variant	*PRTG*	0.1658	0.1471	0.2596	0.1548	0.1443	0.2832
rs75403514	15	55673468	A	G	PRTG	0.0126531	0.861598	51441	-0.428758	0.0289269	219.696	1.053E-49	48.9774	synonymous_variant	*PRTG*	0.2448	0.1609	0.1281	0.2574	0.1576	0.1025
rs62017992	15	55649336	T	C	PRTG	0.0403764	0.999568	51441	-0.223019	0.0152453	213.998	1.844E-48	47.7343	intron_variant	*PRTG*	-0.0366	0.1366	0.7889	-0.0401	0.1347	0.7662
rs4601983	15	55634902	T	C	PRTG	0.676782	0.981231	51441	0.0923571	0.00651138	201.184	1.152E-45	44.9386	intron_variant	*PRTG*	-0.0125	0.0553	0.8208	-0.0109	0.0543	0.8412
rs72748421	15	55625819	C	T	PRTG	0.184133	0.901103	51441	0.113027	0.00821366	189.362	4.382E-43	42.3583	intron_variant	*PRTG*	-0.0821	0.0657	0.2115	-0.0759	0.0644	0.2392
rs62043873	15	55709102	C	T	PRTG	0.0306514	0.867622	51441	-0.252733	0.0186821	183.008	1.068E-41	40.9714	intron_variant	*PRTG*	-0.0405	0.1378	0.7686	-0.0374	0.1359	0.7835
rs150799270	15	55646569	G	A	PRTG	0.0334502	0.938375	51441	-0.23232	0.017224	181.931	1.836E-41	40.7361	intron_variant	*PRTG*	-0.1159	0.1473	0.4312	-0.1236	0.1453	0.3951

SNP, Single Nucleotide Polymorphism; CHROM, Chromosome; POS, Position; A1, Minor allele; A2, Major allele; MAF, Minor allele frequency; N, Number of samples; CHISQ, Chisquare; LOG10P, Log 10 P-value, SE, Standard Error

*Condition analysis 1: Adjusted for age, sex, three principal components and clinical diagnosis status

#Condition analysis 2: Adjusted for age, sex, three principal components, *APOE* ε4 status and clinical diagnosis status

Supplementary Table 18. Gene pathways associated with tau deposition

ID	Description	SetSize	enrichmentScore	NES	*P*-value	*p*.adjust	*q*value	rank	leading_edge	Database
GO:0032373	positive regulation of sterol transport	37	0.711638705	1.6385708	7.57E-06	0.03	0.03	1667	tags=32%, list=10%, signal=29%	GO Biological Process
GO:0032376	positive regulation of cholesterol transport	37	0.711638705	1.6385708	7.57E-06	0.03	0.03	1667	tags=32%, list=10%, signal=29%	GO Biological Process
GO:0032371	regulation of sterol transport	61	0.645179106	1.5316761	1.27E-05	0.03	0.03	1.67E+03	tags=28%, list=10%, signal=25%	GO Biological Process
GO:0032374	regulation of cholesterol transport	61	0.645179106	1.5316761	1.27E-05	0.03	0.03	1.67E+03	tags=28%, list=10%, signal=25%	GO Biological Process
GO:0005916	fascia adherens	9	0.897600495	1.7956496	4.07E-05	0.05	0.05	428	tags=33%, list=3%, signal=33%	GO Cellular Component
GO:0099572	postsynaptic specialization	337	0.489025146	1.2366171	1.17E-04	0.05	0.05	4273	tags=35%, list=25%, signal=26%	GO Cellular Component
GO:0032279	asymmetric synapse	327	0.48910016	1.2364019	7.84E-05	0.05	0.05	4273	tags=34%, list=25%, signal=26%	GO Cellular Component
GO:0030669	clathrin-coated endocytic vesicle membrane	66	0.603135862	1.4423482	2.01E-04	0.05	0.05	2784	tags=30%, list=17%, signal=25%	GO Cellular Component
GO:0014069	postsynaptic density	314	0.488380532	1.2342847	2.05E-04	0.05	0.05	4273	tags=34%, list=25%, signal=26%	GO Cellular Component
GO:0098984	neuron to neuron synapse	354	0.47912675	1.21266	2.48E-04	0.06	0.05	4273	tags=33%, list=25%, signal=25%	GO Cellular Component
GO:0030301	cholesterol transport	105	0.573677492	1.4003494	4.69E-05	0.10	0.10	1667	tags=24%, list=10%, signal=22%	GO Biological Process
hsa02010	ABC transporters	43	0.623976475	1.4541347	6.00E-04	0.21	0.20	2242	tags=33%, list=13%, signal=28%	KEGG
GO:1905954	positive regulation of lipid localization	105	0.565443155	1.3802493	1.21E-04	0.22	0.21	2140	tags=27%, list=13%, signal=23%	GO Biological Process
GO:0072319	vesicle uncoating	8	0.891151466	1.7431713	1.45E-04	0.22	0.22	1312	tags=50%, list=8%, signal=46%	GO Biological Process
GO:0086042	cardiac muscle cell-cardiac muscle cell adhesion	7	0.905338982	1.7340558	1.75E-04	0.24	0.23	2	tags=14%, list=0%, signal=14%	GO Biological Process
hsa04310	Wnt signaling pathway	166	0.511570031	1.2681449	1.40E-03	0.25	0.24	3781	tags=33%, list=22%, signal=26%	KEGG

NES, Normalized Enrichment Score; p.adjust, Adjusted P-value.

Supplementary Table 19. Gene pathways commonly associated with tau and amyloid pathologies

ID	Description	SetSize	enrichmentScore	NES	*P*-value	*p*.adjust	*q*value	rank	leading_edge	Database
GO:0006897	endocytosis	3	0.9622642	2.167127	0.0008921	0.2221281	0.2221281	5	tags=100%, list=9%, signal=96%	GO Biological Process
GO:0006898	receptor-mediated endocytosis	3	0.9622642	2.167127	0.0008921	0.2221281	0.2221281	5	tags=100%, list=9%, signal=96%	GO Biological Process
GO:0030139	endocytic vesicle	3	0.9056604	1.9977257	0.0038247	0.1529894	0.1529894	8	tags=100%, list=14%, signal=91%	GO Cellular Component
GO:0030666	endocytic vesicle membrane	3	0.9056604	1.9977257	0.0038247	0.1529894	0.1529894	8	tags=100%, list=14%, signal=91%	GO Cellular Component
R-HSA-162582	Signal Transduction	12	0.469697	1.7690894	0.0154716	0.0154716	NA	12	tags=58%, list=21%, signal=58%	Reactome

NES, Normalized Enrichment Score; p.adjust, Adjusted P-value.

Supplementary Table 20. Association of tau and amyloid PGS on clinical and neuropathological measures of AD

Phenotypes	All subjects				*APOE* ε4 carriers				*APOE* non-ε4 carriers			
	Amyloid PGS		Tau PGS		Amyloid PGS		Tau PGS		Amyloid PGS		Tau PGS	
	P-value	**R^2**	***P-value***	**R^2**	***P-value***	**R^2**	***P-value***	**R^2**	***P-value***	**R^2**	***P-value***	**R^2**
Clinical diagnosis (CN and AD)	0.0177	0.00377	0.2852	0.00076	0.4857	0.0013	0.0149	0.01675	0.4885	0.00044	0.7782	7.30E-05
NIA-Reagan Diagnosis of Alzheimer's disease	0.9733	9.30E-07	0.9059	1.20E-05	0.6983	0.0006	0.1512	0.00836	0.8565	3.50E-05	0.3745	0.00084
Definite presence of Hippocampal Sclerosis	0.97	1.60E-06	0.34	0.0006	0.674	0.0009	0.2124	0.0078	0.456	0.0013	0.0608	0.0082

CN, Cognitively Normal; AD, Alzheimer's disease; PGS, Polygenic score

Supplementary Table 21. Mediation analysis of rs78636169 effects on cortical thickness via tau SUVR across Braak stage regions in ADNI cohort

Braak stage	Predictor	Mediator	Outcome	N	Indirect effect (ACME)		Direct effect		Total effect	
					β (95% CI)	*P*-value	β (95% CI)	*P*-value	β (95% CI)	*P*-value
Braak 1-2	rs78636169	Tau SUVR	Cortical atrophy	375	-0.109 (-0.203, -0.037)	4.00E-04	-0.093 (-0.258, 0.055)	0.24	-0.202 (-0.391, -0.035)	0.02
Braak 3-4	rs78636169	Tau SUVR	Cortical atrophy	375	-0.032 (-0.058, -0.01)	1.20E-03	-0.034 (-0.085, 0.015)	0.17	-0.066 (-0.12, -0.013)	0.01
Braak 5-6	rs78636169	Tau SUVR	Cortical atrophy	375	-0.017 (-0.036, -0.004)	1.60E-03	-0.017 (-0.057, 0.022)	0.41	-0.033 (-0.074, 0.005)	0.09
Braak 1-6	rs78636169	Tau SUVR	Cortical atrophy	375	-0.053 (-0.096, -0.019)	4.00E-04	-0.047 (-0.129, 0.027)	0.23	-0.1 (-0.185, -0.023)	0.01

SUVR, Standard Uptake Value Ratio; ACME, Average Causal Mediation Effect; CI, Confidence Interval

Supplementary Table 22. Mediation analysis of rs78636169 effects on cortical thickness via tau SUVR across Braak stage regions in A4 cohort

Braak stage	Predictor	Mediator	Outcome	N	Indirect effect (ACME)		Direct effect		Total effect	
					β (95% CI)	*P*-value	β (95% CI)	*P*-value	β (95% CI)	*P*-value
Braak 1-2	rs78636169	Tau SUVR	Cortical atrophy	311	-0.044 (-0.116, 0.008)	0.12	0.15 (-0.23, 0.5)	0.41	0.11 (-0.29, 0.47)	0.58
Braak 3-4	rs78636169	Tau SUVR	Cortical atrophy	311	0.043 (-0.016, 0.146)	0.16	0.21 (-0.27, 0.66)	0.42	0.26 (-0.2, 0.69)	0.29
Braak 5-6	rs78636169	Tau SUVR	Cortical atrophy	311	0.044 (-0.025, 0.134)	0.2	0.1 (-0.28, 0.43)	0.59	0.14 (-0.21, 0.46)	0.41
Braak 1-6	rs78636169	Tau SUVR	Cortical atrophy	311	0.017 (-0.057, 0.117)	0.63	0.16 (-0.27, 0.53)	0.45	0.18 (-0.23, 0.55)	0.39

SUVR, Standard Uptake Value Ratio; ACME, Average Causal Mediation Effect; CI, Confidence Interval

Supplementary Table 23. SNPs in *MAPT* gene associated with tau deposition

SNP	Gene	CHR	BP	Variant type	Effect	ADNI cohort				A4 cohort				Tau-SUVR Meta-analysis						
						Beta	SE	*P*-value	MAF	Beta	SE	*P*-value	MAF	Beta	SE	*P*-value	Dir	HetISq	HetChiSq	HetPVal
rs117977938	*MAPT*	17	44043717	intron_variant	C	0.4477	0.1664	0.007447	0.04094	0.2685	0.2412	0.2665	0.03055	0.39	0.137	0.004423	++	0	0.373	0.5412
rs116204525	*MAPT*	17	44046192	upstream_gene_variant	C	0.4477	0.1664	0.007447	0.04094	0.2685	0.2412	0.2665	0.03055	0.39	0.137	0.004424	++	0	0.373	0.5413
rs63750072	*MAPT*	17	44060859	missense_variant	G	0.47	0.1486	0.001691	0.05211	0.09646	0.2069	0.6414	0.0418	0.3431	0.1208	0.004489	++	53.4	2.146	0.143
rs2435207	*MAPT*	17	44058928	upstream_gene_variant	A	0.1739	0.06949	0.01276	0.3151	0.08186	0.08437	0.3327	0.328	0.1368	0.0537	0.01083	++	0	0.708	0.4001
rs34619181	*MAPT*	17	44051409	5_prime_UTR_variant	T	0.2553	0.0868	0.003474	0.1911	0.04385	0.1061	0.6797	0.1768	0.1707	0.0672	0.01112	++	57.9	2.375	0.1233
rs13313562	*MAPT*	17	44056097	upstream_gene_variant	C	0.258	0.0875	0.003397	0.1886	0.0429	0.1062	0.6865	0.1752	0.1712	0.0676	0.0113	++	59	2.44	0.1183
rs4792894	*MAPT*	17	43999203	intron_variant	A	-0.1787	0.1056	0.0914	0.1042	-0.2662	0.1473	0.07178	0.08521	-0.2084	0.0859	0.01524	--	0	0.233	0.6296
rs2435206	*MAPT*	17	44058111	upstream_gene_variant	T	0.1378	0.07053	0.05155	0.304	0.08982	0.08343	0.2825	0.3215	0.1178	0.0539	0.02878	++	0	0.193	0.6608
rs76281607	*MAPT*	17	44042782	intron_variant	T	0.2118	0.089	0.01783	0.1737	0.05188	0.1077	0.6303	0.1624	0.147	0.0686	0.03222	++	23.6	1.308	0.2527
rs77169816	*MAPT*	17	44042942	intron_variant	T	0.2118	0.089	0.01783	0.1737	0.05188	0.1077	0.6303	0.1624	0.147	0.0686	0.03222	++	23.6	1.308	0.2527
rs3785883	*MAPT*	17	44054433	5_prime_UTR_variant	A	0.2053	0.08888	0.02144	0.1749	0.05574	0.1071	0.6032	0.1672	0.1444	0.0684	0.03485	++	13.3	1.153	0.2829
rs2435209	*MAPT*	17	44061893	upstream_gene_variant	T	0.132	0.07169	0.06648	0.2965	0.08951	0.08483	0.2922	0.3119	0.1143	0.0548	0.03691	++	0	0.146	0.7023
rs2435210	*MAPT*	17	44061894	upstream_gene_variant	T	0.132	0.07169	0.06648	0.2965	0.08951	0.08483	0.2922	0.3119	0.1143	0.0548	0.03691	++	0	0.146	0.7023
rs66499584	*MAPT*	17	44091780	intron_variant	A	0.2522	0.09423	0.007786	0.1489	-0.001097	0.118	0.9926	0.1399	0.1537	0.0737	0.03692	+-	64.4	2.809	0.09373
rs16940758	*MAPT*	17	44040626	intron_variant	T	0.202	0.08957	0.02472	0.1687	0.04582	0.108	0.6715	0.1608	0.1385	0.069	0.04474	++	19.2	1.237	0.266
rs2435211	*MAPT*	17	44063244	downstream_gene_variant	T	0.1321	0.07321	0.07209	0.2928	0.08246	0.08548	0.3355	0.3119	0.1111	0.0556	0.04578	++	0	0.194	0.6594

SNP, Single Nucleotide Polymorphisms; Chr, Chromosome; A1, Effect allele; MAF, Minor Allele Frequency; SE, Standard error; Dir, Effect direction;
Het ChiSq, Chi-square value for heterogeneity test; Het PVal, *P*-value for heterogeneity in effect sizes in meta-analysis.
