# Supplementary material for: Common and rare variant analyses implicate JARID2 in cerebral tau deposition
Source: NPJ Dement. 2026 Jul 3;2(1):52. doi: 10.1038/s44400-026-00107-6 (PMC13331746; doi:10.1038/s44400-026-00107-6)
Supplement: Supplementary file 4 — Supplementary Information [file 44400_2026_107_MOESM4_ESM.pdf]

A4 AND LEARN STUDY TEAM LIST

The A4 and LEARN Study Leadership Teams provide the list below for standardized acknowledgement of the many individuals who have contributed to the A4 and LEARN Studies (current and former). The list consists of five parts: I. Funding Partners, II. Study Leadership Team, III. Team Leaders and Key Personnel, IV. Institutional Study Partners, and V. Study Sites. All manuscripts, including methodological papers, should include an acknowledgement of the A4 and LEARN Study Teams. This information is current as of May 1, 2023.

Reisa Sperling, MD (Harvard Medical School, Brigham and Women's Hospital, Massachusetts General Hospital, Leadership Team); Paul Aisen, MD (University of Southern California, Alzheimer's Therapeutic Research Institute, Leadership Team); Roy Yaari, MD (Eli Lilly and Company, Leadership Team); Cheryl A. Brown, RPh, PMP (Eli Lilly and Company, Leadership Team); John R. Sims, MD (Eli Lilly and Company, Leadership Team); Keith Johnson, MD (PET Imaging, Team Leader); Clifford Jack Jr., MD (MRI – Mayo Clinic, Team Leader); James B. Brewer, MD, PhD (ADCS Imaging, Team Leader); Jason Karlawish, MD (Ethics Committee, Team Leader); Joshua D. Grill, PhD (Ethics Committee, Team Leader) Marybeth Howlett, MEM (AVID, Team Leader); Paul Maruff, PhD (Cogstate, Team Leader); Kenneth Marek, MD (Invicro, Team Leader); John Seibyl, MD (Invicro, Team Leader); Mark Mintun, MD (Eli Lilly and Company, Team Leader); Karen Holdridge, MPH (Eli Lilly and Company, Team Leader); Isabella Velona, MS (Eli Lilly and Company, Team Leader); Alison Belsha, BS (University of Southern California, Alzheimer's Therapeutic Research Institute, Team Leader); Jeremy Pizzola (University of Southern California, Alzheimer's Therapeutic Research Institute, Team Leader); Robert Rissman, PhD (University of Southern California, Alzheimer's Therapeutic Research Institute, Team Leader); Cecily Jenkins, PhD (University of Southern California, Alzheimer's Therapeutic Research Institute, Team Leader); Michael Donohue, PhD (University of Southern California, Alzheimer's Therapeutic Research Institute, Team Leader); Rema Raman, PhD (University of Southern California, Alzheimer's Therapeutic Research Institute, Team Leader); Gustavo Jimenez-Maggiora, MBA (University of Southern California, Alzheimer's Therapeutic Research Institute, Team Leader); Mike Rafii, MD, PhD (University of Southern California, Alzheimer's Therapeutic Research Institute, Team Leader); Aaron Schultz, PhD (Brigham and Women's Hospital, Massachusetts General Hospital, Harvard Medical School); Dorene Rentz, PsyD (Brigham and Women's Hospital, Massachusetts General Hospital, Harvard Medical School); Kate Papp, PhD (Brigham and Women's Hospital, Massachusetts General Hospital, Harvard Medical School); Beth Mormino, PhD (Brigham and Women's Hospital, Massachusetts General Hospital, Harvard Medical School); Rebecca Amariglio, PhD (Brigham and Women's Hospital, Massachusetts General Hospital, Harvard Medical School); Gad Marshall, MD (Brigham and Women's Hospital, Massachusetts General Hospital, Harvard Medical School); Dylan Kirn (Brigham and Women's Hospital, Massachusetts General Hospital, Harvard Medical School); Michael Properzi (Brigham and Women's Hospital, Massachusetts General Hospital, Harvard Medical School); J. Alex Becker, PhD (Brigham and Women's Hospital, Massachusetts General Hospital, Harvard Medical School); Rachel Buckley, PhD (Brigham and Women's Hospital, Massachusetts General Hospital, Harvard Medical School); Jorge Gutierrez (University of Southern California, Alzheimer's Therapeutic Research Institute, Clinical Operations); Harriett Davey (University of Southern California, Alzheimer's Therapeutic Research Institute, Clinical Operations); Melissa Ruiz (University of Southern California, Alzheimer's Therapeutic Research Institute, Clinical Operations); Vedeline Torreon, BS (University of Southern California, Alzheimer's

Therapeutic Research Institute, Medical Safety/Clinical Monitoring); Marianne Manire, BS (University of Southern California, Alzheimer's Therapeutic Research Institute, Medical Safety/Clinical Monitoring); Renarda Jones, MS (University of Southern California, Alzheimer's Therapeutic Research Institute, Medical Safety/Clinical Monitoring); Isabel Francis (University of Southern California, Alzheimer's Therapeutic Research Institute, Clinical Operations); Maria Arampatzidou, PhD (University of Southern California, Alzheimer's Therapeutic Research Institute, Clinical Operations); Paula Cohen, BA (University of Southern California, Alzheimer's Therapeutic Research Institute, Clinical Operations); Jennifer Salazar, MBS (University of Southern California, Alzheimer's Therapeutic Research Institute, Clinical Operations); Gabriela Muranevici, MD, PhD (University of Southern California, Alzheimer's Therapeutic Research Institute, Former Medical Monitor, Medical Safety/Clinical Monitoring); Tiffany Chow, MD (University of Southern California, Alzheimer's Therapeutic Research Institute, Former Director, Clinical Monitoring, Medical Safety/Clinical Monitoring); Steve Bruno III, BA (University of Southern California, Alzheimer's Therapeutic Research Institute, Former Lead Clinical Monitor, Medical Safety/Clinical Monitoring); Gina Garcia-Camilo, MD (University of Southern California, Alzheimer's Therapeutic Research Institute, Former Clinical Monitor, Medical Safety/Clinical Monitoring); Adriana Bohorquez, MD (University of Southern California, Alzheimer's Therapeutic Research Institute, Former Medical Monitor, Medical Safety/Clinical Monitoring); Alyssa Carroll (Schmitt), MS (University of Southern California, Alzheimer's Therapeutic Research Institute, Former Clinical Monitor Manager, Medical Safety/Clinical Monitoring); Sarah Danowski, MA (University of Southern California, Alzheimer's Therapeutic Research Institute, Former Clinical Monitor Manager, Medical Safety/Clinical Monitoring); Deborah Tobias (University of Southern California, Alzheimer's Therapeutic Research Institute, Former Administration Director); Lindsey Earp, BA (University of Southern California, Alzheimer's Therapeutic Research Institute, Administration); Dan Abinsay (University of Southern California, Alzheimer's Therapeutic Research Institute, Administration); Sarah Walter, MSc (University of Southern California, Alzheimer's Therapeutic Research Institute, Administration); Ryoko Ihara, MD, PhD (University of Southern California, Alzheimer's Therapeutic Research Institute, Neuropsychology); Cecily Jenkins, PhD (University of Southern California, Alzheimer's Therapeutic Research Institute, Neuropsychology); Xavier Salazar, PhD (University of Southern California, Alzheimer's Therapeutic Research Institute, Neuropsychology); Stefanie Juliano, MA (University of Southern California, Alzheimer's Therapeutic Research Institute, Neuropsychology); Sarah Espinoza, BA (University of Southern California, Alzheimer's Therapeutic Research Institute, Neuropsychology); Shelley Moore, BA (University of Southern California, Alzheimer's Therapeutic Research Institute, Recruitment & Retention, Administration); Taylor Clanton, MPH, CHES (University of Southern California, Alzheimer's Therapeutic Research Institute, Recruitment & Retention); Agnes Lewandowski, M.Res (University of Southern California, Alzheimer's Therapeutic Research Institute, Recruitment & Retention); Phuoc Hong, BA (University of Southern California, Alzheimer's Therapeutic Research Institute, IT); Hongmei Qiu, MS (University of Southern California, Alzheimer's Therapeutic Research Institute, Informatics); Jia-shing So, BS (University of Southern California, Alzheimer's Therapeutic Research Institute, Informatics); Stefania Bruschi, MS, MBA (University of Southern California, Alzheimer's Therapeutic Research Institute, Informatics); Kimberlee Eudy, JD (University of Southern California, Alzheimer's Therapeutic Research Institute, Contracts); Quin Revel, JD (University of Southern California, Alzheimer's Therapeutic Research Institute, Contracts); Michael Selsnik, BS (University of Southern California, Alzheimer's Therapeutic Research Institute, Contracts); Olusegun Adegoke, MSc (University of Southern California, Alzheimer's Therapeutic Research Institute, Data Management); Veasna Tan, MA (University of Southern California, Alzheimer's Therapeutic Research Institute, Data Management); Olga Baryshnikava, MS (University of Southern California, Alzheimer's Therapeutic Research Institute, Data Management); Iris Sim Dacio, BA

(University of Southern California, Alzheimer's Therapeutic Research Institute, Data Management); Sandhya Niranjan Jaiswal, B Pharm (University of Southern California, Alzheimer's Therapeutic Research Institute, Data Management); Elizabeth Shaffer, BS (University of Southern California, Alzheimer's Therapeutic Research Institute, Regulatory Affairs); Michelle Pablo, BS (University of Southern California, Alzheimer's Therapeutic Research Institute, Regulatory Affairs); Karin Ernstrom, MS (University of Southern California, Alzheimer's Therapeutic Research Institute, Biostatistics); Gopalan Sethuraman, PhD (University of Southern California, Alzheimer's Therapeutic Research Institute, Biostatistics); Jiyoon Choi, MS (University of Southern California, Alzheimer's Therapeutic Research Institute, Biostatistics); Oliver Langford, MS (University of Southern California, Alzheimer's Therapeutic Research Institute, Biostatistics); Shunran Wang, MS (University of Southern California, Alzheimer's Therapeutic Research Institute, Biostatistics); Andy Liu, MS (University of Southern California, Alzheimer's Therapeutic Research Institute, Biostatistics); Barbara Bartocci, MPH (University of Southern California, Alzheimer's Therapeutic Research Institute, Quality Assurance); Melissa Korba (University of Southern California, Alzheimer's Therapeutic Research Institute, Quality Assurance); Sara Abdel-Latif, MBA (University of Southern California, Alzheimer's Therapeutic Research Institute, Biomarker); Andrea Abram, MBA (Eli Lilly and Company); Bhavna Madduri, MPH (Eli Lilly and Company); Dillon Hilderbrand (Eli Lilly and Company); John Brad Holmes, MBA (Eli Lilly and Company); Julie Chandler, PhD (Eli Lilly and Company); Keith Parsons, BS, MBA (Eli Lilly and Company); Lisa Ferguson-Sells, BSc (Eli Lilly and Company); Marybeth Devine (Eli Lilly and Company); Michael Case, MS (Eli Lilly and Company); Michele Mancini, MD (Eli Lilly and Company); Michael Pontecorvo, PhD (Eli Lilly and Company); Peter Fairfield, MBA (Eli Lilly and Company); Phyllis Ferrell, MBA (Eli Lilly and Company); Satina Hall (Eli Lilly and Company); Scott Kaiser, MBA (Eli Lilly and Company); Sergey Shcherbinin, PhD (Eli Lilly and Company); Stacy Huckins (Eli Lilly and Company); Susan Warner, Pharm.D (Eli Lilly and Company); Jude Burger, MS (Eli Lilly and Company); Bret J. Borowski, RT (R) (MRI – Mayo Clinic); Petrice M. Cogswell, MD (MRI – Mayo Clinic); Cory A. Johnson (MRI – Mayo Clinic); Kejal Kantarci, MD (MRI – Mayo Clinic); Leonard C. Matoush, Jr. (MRI – Mayo Clinic); William C. Turke (MRI – Mayo Clinic); Ashritha L. Reddy (MRI – Mayo Clinic); Denise A. Reyes (MRI – Mayo Clinic); Kaely B. Thostenson, RT (R) (MRI – Mayo Clinic); Samantha M. Zuk (MRI – Mayo Clinic); Jeffrey M. Burns, MD, MS (A4 Study Ethics Committee, University of Kansas Alzheimer's Disease Center, Member); David Sultzer, MD (A4 Study Ethics Committee, University of California, Irvine, Member); Howard Feldman, MD (Alzheimer's Disease Cooperative Study [ADCS] – University of California, San Diego, Director (Lead)); Genny Matthews (ADCS – University of California, San Diego, Recruitment); Stephanie Parks (ADCS – University of California, San Diego, Clinical Operations); Jen Mason, MPH (ADCS – University of California, San Diego, Data Management); Jason Young, PhD (ADCS – University of California, San Diego, Informatics); Ashlee Heldreth, BA (ADCS – University of California, San Diego, Monitor); Janet Kastelan, BA (ADCS – University of California, San Diego, Monitor); Rebecca Ryan-Jones, PhD (ADCS – University of California, San Diego, Monitor); Lindsay Cotton (ADCS – University of California, San Diego, Monitor); Maria Bulger Lennox, RN (ADCS – University of California, San Diego, Monitor); Ronelyn Chavez, BA (ADCS – University of California, San Diego, Monitor); Tilman Oltersdorf, MD (ADCS – University of California, San Diego, MedSafety); Curtis Taylor, PhD (ADCS – University of California, San Diego, MedSafety); Barbara LaPlante, MA (ADCS – University of California, San Diego, Transition Team); Meghan Stirn, MBA (ADCS – University of California, San Diego, Transition Team); Joanne Brechlin, MBA, MPH (ADCS – University of California, San Diego, Transition Team); Gina Varner, MPH (ADCS – University of California, San Diego, Transition Team); Carol Evans, BA (ADCS – University of California, San Diego, U19 Grant Administration); Karim Hussein, JD (ADCS – University of California, San Diego, U19 Grant Administration); Erika Wilson (ADCS – University of California, San Diego, Finance); Ronald Thomas, PhD (ADCS – University of California, San

Diego, Biostatistics); Sheila Jin, PhD (ADCS – University of California, San Diego, Biostatistics); James Barlow (ADCS – University of California, San Diego, Biomarker); Nichol Ferng, BS (ADCS Neuroimaging – University of California, San Diego); Robin Jennings, BS, MS (ADCS Neuroimaging – University of California, San Diego); Leonardino Digma, BA (ADCS Neuroimaging – University of California, San Diego); Heidi Jacobs, PhD (PET Imaging, Massachusetts General Hospital); Jorge Sepulcre, MD (PET Imaging, Massachusetts General Hospital); Justin Sanchez (PET Imaging, Massachusetts General Hospital); Matthew Scott (PET Imaging, Massachusetts General Hospital); Reneé Tschopp, MS, PMP (Invicro); Donna Miles (Invicro); Amy Frederickson, GradDipBiostats (Cogstage, Melbourne); François Windels, PhD (Cogstate, Brisbane); Bodil Hook, BBSc, PostGradDip in Psychology (Cogstage, Melbourne); Lenny Nuciforo, BS (Cogstage, New York, NY); Natalia Contreras, PhD (Cogstage, Melbourne); Patrick McCabe, BA (McCabe Message Partners); Becky Watt Knight, MA (McCabe Message Partners); Rachel Griffith, BA (McCabe Message Partners); Melissa McGue, BS, BA (McCabe Message Partners); Shannon Conti, MS (McCabe Message Partners); Amy Martin Vogt, BA (McCabe Message Partners); Marissa C. Natelson Love, MD (University of Alabama, Birmingham); P. Denise Ledlow, RN (University of Alabama, Birmingham); Amber Watkins, RN (University of Alabama, Birmingham); David S. Geldmacher, MD (University of Alabama, Birmingham); Loren Brown Ashley, RN (University of Alabama, Birmingham); Jacqueline Vaughn, RN (University of Alabama, Birmingham); William J. Burke, MD (Banner Alzheimer's Institute); Roma Patel, MS, MBA (Banner Alzheimer's Institute); Daniel Viramontes Apodaca (Banner Alzheimer's Institute); Sachin Y. Pandya (Banner Alzheimer's Institute); Anna D. Burke, MD (Banner Alzheimer's Institute); Edward Zamrini, MD (Banner Sun Health Research Institute); Zoran Obradov, CRC (Banner Sun Health Research Institute); Christine M. Belden, PsyD (Banner Sun Health Research Institute); Carol Cline, MSW, LMSW, CSP (Banner Sun Health Research Institute, Psychometrist); Margaret Rich, CSP (Banner Sun Health Research Institute, Psychometrist); Lisa Roye, MS (Banner Sun Health Research Institute, Psychometrist); Marwan Sabbagh, MD (Banner Sun Health Research Institute, Past Investigator); Jerome Yesavage, MD (Stanford University School of Medicine, VA Aging Clinical Research Center); Steven Z. Chao, MD, PhD (Stanford University School of Medicine, VA Aging Clinical Research Center); Tamara Beale, MA (Stanford University School of Medicine, VA Aging Clinical Research Center); Jaila Coleman, BA (Stanford University School of Medicine, VA Aging Clinical Research Center); Shawn Kile, MD (Sutter Institute for Medical Research); Valentina Mikhalenko, CRC (Sutter Institute for Medical Research); Yvonne Au, LCSW, PACC (Sutter Institute for Medical Research); Mary Vaughn, RN (Sutter Institute for Medical Research); Sampreet Moneski, CRC (Sutter Institute for Medical Research); Tammy Donnell, CCRC (Sutter Institute for Medical Research); Dawn Lenakakis, CRC (Sutter Institute for Medical Research); John Gregory Duffy, MD (Syrentis Clinical Research); Lorrie Bisesi, PhD (Syrentis Clinical Research); Poonam Nina Banerjee, PhD (Syrentis Clinical Research); Maria Gonzalez, BS (Syrentis Clinical Research); Rania Bilwani, BS (Syrentis Clinical Research); John Olichney, MD (University of California, Davis Alzheimer's Disease Center East Bay); Charles DeCarli, MD (University of California, Davis Alzheimer's Disease Center East Bay); Hongzheng Zhang, PhD (University of California, Davis Alzheimer's Disease Center East Bay); Antoinette Lopez, MD (University of California, Davis Alzheimer's Disease Center East Bay); Mary McPhail-Ciufo, MD (University of California, Davis Alzheimer's Disease Center East Bay); Adrian Preda, MD (University of California, Irvine [UCI BIC]); Andrea Weideman (University of California, Irvine [UCI BIC]); Steven Potkin, MD (University of California, Irvine [UCI BIC], Past Investigator); Melanie Tallakson, DNP (University of California, Irvine [UCI MIND]); Catherine McAdams-Ortiz, AGNP (University of California, Irvine [UCI MIND]); Beatriz Vides, MSN, RN (University of California, Irvine [UCI MIND]); Gaby T. Thai, MD (University of California, Irvine [UCI MIND]); Steven P. Tam, MD (University of California, Irvine [UCI MIND]); Maryam Beigi, MD (University of California, Los Angeles); Thao Rodriguez, NP (University of California, Los Angeles); Maya Farchi, CRC (University of

California, Los Angeles); Roberto Hernandez, CRC (University of California, Los Angeles); Lorena Monserratt, PhD (University of California, Los Angeles); Lauren Garcia, CRC (University of California, Los Angeles); Celine Ossinalde, MA (University of California, Los Angeles); Douglas Galasko, MD (University of California, San Diego); Helen Vanderswag, RNC, BSN (University of California, San Diego); Laura Linares, RN (University of California, San Diego); Chi Kim, BS (University of California, San Diego); Shawnees Peacock, BS (University of California, San Diego); Adam L. Boxer, MD, PhD (University of California, San Francisco, Memory and Aging Center); Lawren Vandevrede, MD, PhD (University of California, San Francisco, Memory and Aging Center); Peter Ljubenkov, MD (University of California, San Francisco, Memory and Aging Center); Julio C. Rojas, MD, PhD (University of California, San Francisco, Memory and Aging Center); Mauricio Becerra (University of Southern California, Alzheimer's Disease Research Center); Liberty Teodoro, RN (University of Southern California, Alzheimer's Disease Research Center); Sonia Pawluczyk, MD (University of Southern California, Alzheimer's Disease Research Center); Karen Dagerman, MS (University of Southern California, Alzheimer's Disease Research Center); Lon Schneider, MD (University of Southern California, Alzheimer's Disease Research Center); Christopher H. van Dyck, MD (Yale Alzheimer's Disease Research Unit); Julia W. McDonald, BA (Yale Alzheimer's Disease Research Unit); Susan P. Good, APRN (Yale Alzheimer's Disease Research Unit); Joanna E. Harris, BA (Yale Alzheimer's Disease Research Unit); Kara Bates, BS (Yale Alzheimer's Disease Research Unit); Jessica Lam, BS (Yale Alzheimer's Disease Research Unit); Raymond Scott Turner, MD, PhD (Georgetown University); Melanie Chadwick, RN, MS, NP (Georgetown University); Kathleen Johnson, RN, MSN, NP (Georgetown University); Brigid Reynolds, RN, MSN, NP (Georgetown University); Kelly McCann, BA (Georgetown University); Thomas O. Obisesan, MD, MPH (Howard University); Oyonumo E. Ntekim, MD, PhD (Howard University); Sheeba R. Nadarajah, PhD (Howard University); Sharlene Leong, MSc (Howard University); Saba Wolday, MSc (Howard University); Jillian Turner (Howard University); Mark Brody, MD (Brain Matters Research); Paayal Patel, MD (Brain Matters Research); Cynthia Stimeck, PA-C (Brain Matters Research); Neill R. Graff-Radford, MD (Mayo Clinic – Jacksonville); Christopher Homa, BS (Mayo Clinic – Jacksonville); Amanda Phillips, BS (Mayo Clinic – Jacksonville); Paul Winner, DO (Premiere Research Institute); Alfonso X. Moreno, MD (Premiere Research Institute); Esteban Olivera, MD (Synexus Clinical Research, Orlando); Jennifer West, PA (Synexus Clinical Research, Orlando); Noureen Dhanani, APRN (Synexus Clinical Research, Orlando, LEARN Study PI); Maria Amy Edridge, L-CRC (Synexus Clinical Research, Orlando); Alisa Petit, MS, CSP (Synexus Clinical Research, Orlando); Yarnick Mirjah, BS (Synexus Clinical Research, Orlando); Olivia Reilly (Synexus Clinical Research, Orlando); Angelica Okolie, MD (Synexus Clinical Research, The Villages); Uzma Khan, MD (Synexus Clinical Research, The Villages); Elma Fallejo (Synexus Clinical Research, The Villages); Amanda G. Smith, MD (USF Health Byrd Alzheimer's Institute); Juris Jarvis, MD (USF Health Byrd Alzheimer's Institute); Kelly Rodrigo, BA, CCRC (USF Health Byrd Alzheimer's Institute); Ijeoma Mba, MBBS (USF Health Byrd Alzheimer's Institute); Anna D. Sladky, CIP, CRA-USF (USF Health Byrd Alzheimer's Institute); Patricia Lowe, CCRC, CRA-USF (USF Health Byrd Alzheimer's Institute); Breanna Davis (USF Health Byrd Alzheimer's Institute); Ranjan Duara, MD (Wien Center for Alzheimer's Disease and Dementia); Maria T. Greig Custo, MD (Wien Center for Alzheimer's Disease and Dementia); Rosemarie A. Rodriguez, PhD (Wien Center for Alzheimer's Disease and Dementia); Julieth Formosa, PharmD (Wien Center for Alzheimer's Disease and Dementia); Warren Barker, MA (Wien Center for Alzheimer's Disease and Dementia); Joyce Lee, PharmD (Wien Center for Alzheimer's Disease and Dementia); Cesar Chirinos (Wien Center for Alzheimer's Disease and Dementia); James J. Lah, MD, PhD (Emory University); Allan I. Levey, MD, PhD (Emory University); Deborah Westover, BSN, RN (Emory University); Gail Schwartz, BSN, RN (Emory University); Lauren Mariotti, BS (Emory University); Jeffrey Ross, MD (Great Lakes Clinical Trials); Linda Rice, PhD (Great Lakes Clinical Trials); Sandra Weintraub, PhD (Northwestern University); Ian Grant,

MD (Northwestern University); Brittanie Muse, MSPH, CCRC (Northwestern University); Shea Gold, MA (Northwestern University); Jelena Pejic, BS (Northwestern University); Loreece Haddad, MS (Northwestern University); Neelum T. Aggarwal, MD (Rush University Medical Center); Ajay Sood, MD, PhD (Rush University Medical Center); Kimberly Blanchard, DNP, APRN, NP-C (Rush University Medical Center); Peter Lambiotis (Rush University Medical Center); Amelia Troutman (Rush University Medical Center); Martin Farlow, MD (Indiana University); Jared Brosch, MD (Indiana University); Nancy McClaskey, RN (Indiana University); Del D. Miller, PharmD, MD (University of Iowa); Hristina K. Koleva, MD (University of Iowa); Karen Ekstam Smith, RN (University of Iowa); Laura Temple, MS (University of Iowa); Susan Schultz, MD (University of Iowa, Past Investigator); Anne Arthur, APRN (University of Kansas Medical Center Alzheimer's Disease Center); Rachel Starr, BS, CCRP (University of Kansas Medical Center Alzheimer's Disease Center); Nicole Mathis, MS (University of Kansas Medical Center Alzheimer's Disease Center); Phyllis Switzer (University of Kansas Medical Center Alzheimer's Disease Center); Gregory A. Jicha, MD, PhD (University of Kentucky); Andrea L. Shaffer, BS (University of Kentucky); Sarah Hatch, MSW (University of Kentucky); Sierra Fuhrmann, BS (University of Kentucky); Molly Harper, MSc (University of Kentucky); Kelly Parsons, MSW (University of Kentucky); Jeffrey N. Keller, PhD (Pennington Biomedical Research Center); William P. Gahan, MD (Pennington Biomedical Research Center); Robert Brouillette, MS (Pennington Biomedical Research Center); Heather Foil, MS (Pennington Biomedical Research Center); Owen Carmichael, PhD (Pennington Biomedical Research Center); Paul B. Rosenberg, MD (Johns Hopkins University); Meghan Schultz, RN, MSN (Johns Hopkins University); Samantha Schwartz (Johns Hopkins University); Samantha Horn (Johns Hopkins University); Mersania Jn Pierre (Johns Hopkins University); Robert A. Stern, PhD (Boston University School of Medicine); Jane Mwicigi, MBChB, MPH (Boston University School of Medicine); Alex Puleio, MS (Boston University School of Medicine); Jesse Mez, MD, MS (Boston University School of Medicine); Wendy Qiu, MD, PhD (Boston University School of Medicine); Eric Steinberg, MSN, RN, CS, CANP (Boston University School of Medicine); Tia Hall, BS (Brigham and Women's Hospital); Emily Sprague, BS (Brigham and Women's Hospital); Mariana Palou, BS (Brigham and Women's Hospital); Martha Vander Vliet, RN (Brigham and Women's Hospital); Jaimie Ziolkowski, MA, CCRP (University of Michigan); Judith L. Heidebrink, MD, MS (University of Michigan); Bekkie Wang (University of Michigan); David S. Knopman, MD (Mayo Clinic – Rochester); Bronwyn Briseno, RN (Mayo Clinic – Rochester); Jonathan Graff Radford, MD (Mayo Clinic – Rochester); Sara Mason, RN (Mayo Clinic – Rochester); Karen Kuntz (Mayo Clinic – Rochester); Kari Baxter (Mayo Clinic – Rochester); Randall Bateman, MD (Washington University School of Medicine); Joy Snider, MD, PhD (Washington University School of Medicine); Gregory Day, MD (Washington University School of Medicine); Nupur Ghoshal, MD, PhD (Washington University School of Medicine); Erik Musiek, MD, PhD (Washington University School of Medicine); Tammie Benzinger, MD, PhD (Washington University School of Medicine); John Morris, MD (Washington University School of Medicine); Marta Santos, BSN, RN (Washington University School of Medicine); Daniel L. Murman, MD, MS (University of Nebraska Medical Center); Haley Kampschneider, BS (University of Nebraska Medical Center); Deb Heimes, BS (University of Nebraska Medical Center); Nick Miller, BS (University of Nebraska Medical Center); David Wint, MD (Cleveland Clinic Lou Ruvo Center for Brain Health); Charles Bernick, MD (Cleveland Clinic Lou Ruvo Center for Brain Health); Michelle Torreliza (Cleveland Clinic Lou Ruvo Center for Brain Health); Simrit Saraon, NP (Cleveland Clinic Lou Ruvo Center for Brain Health); Barnett Shpritz (Cleveland Clinic Lou Ruvo Center for Brain Health); Karen L. Bell, MD (Columbia University Medical Center); Ruth Tejeda, MD, MS (Columbia University Medical Center, Past Coordinator); Chismary De La Cruz, BA (Columbia University Medical Center); Lawrence Honig, MD, PhD (Columbia University Medical Center); Betina Idna, PhD, RN (Columbia University Medical Center); Horacio A. Capote, MD (Dent Neurologic Institute); Michelle Rainka, PharmD (Dent Neurologic Institute); Traci

Aladeen, PharmD (Dent Neurologic Institute); Tatiana Jimenez-Knight, MA (Dent Neurologic Institute); Healther MacNamara, BS (Dent Neurologic Institute); Mary Sano, PhD (Icahn School of Medicine at Mount Sinai); Judith Neugroschl, MD (Icahn School of Medicine at Mount Sinai); Joanne Lim (Icahn School of Medicine at Mount Sinai); Allison Ardolino (Icahn School of Medicine at Mount Sinai);Gina Garcia Camilo (Icahn School of Medicine at Mount Sinai); Amy Aloyisi, MD (Icahn School of Medicine at Mount Sinai); Melanie Shulman, MD (NYU Langone Medical Center); Anaztasia Ulysse, BA, CRC (NYU Langone Medical Center); Jamika Singleton-Garvin, CCRC (NYU Langone Medical Center); Mohammed Sheikh, BS, CCRC (NYU Langone Medical Center); Mrunaliniash Gaikwad, BS, CRC (NYU Langone Medical Center); Anton P. Porsteinsson, MD (University of Rochester); Audrey Rice, RN, ANP (University of Rochester); Susan Salem-Spencer, RN, MSN (University of Rochester); Bridget Holvey, MPH, BS (University of Rochester); Asa Widman, BA (University of Rochester); Michael Lin, MD (Weill Cornell Medical Center); Norman Relkin, MD, PhD (Weill Cornell Medical Center); Suzanne Craft, PhD (Wake Forest University School of Medicine); Abigail Heston O'Connell, MS, APRN, NP-C (Wake Forest University School of Medicine); Alexis Webb, MS (Wake Forest University School of Medicine); Bevan Hoover (Wake Forest University School of Medicine); Patricia Wittmer (Wake Forest University School of Medicine); Alan J. Lerner, MD (University Hospitals Cleveland/Case Western Reserve University); Maria Toth, RN (University Hospitals Cleveland/Case Western Reserve University); Parianne Fatica, CCRC (University Hospitals Cleveland/Case Western Reserve University); Susie Sami, MA, (University Hospitals Cleveland/Case Western Reserve University); Paula Ogrocki, PhD (University Hospitals Cleveland/Case Western Reserve University, LEARN Study PI); Marianne Sanders, RN (University Hospitals Cleveland/Case Western Reserve University); Michael Karathanos, MD (Central States Research, LLC); Christy Lisenbee, BS (Central States Research, LLC); Sarah Land, DO (Central States Research, LLC); Carmen Toegel, LPN (Central States Research, LLC); Aimee L. Pierce, MD (Oregon Health and Science University); Lisa C. Silbert, MD (Oregon Health and Science University); Jeffrey A. Kaye, MD (Oregon Health and Science University); Alexandria A. Ruhf, MS (Oregon Health and Science University); Amy B. Thomas, RN (Oregon Health and Science University); Steven Aurich (Oregon Health and Science University); G. Peter Gliebus, MD (Drexel University); Katherine Rife, BS (Drexel University); Melinda Webster, BS (Drexel University); Christine Barr, RN (Drexel University); Monica Mazurek, RN (Drexel University); Sanjeev N. Vaishnavi, MD, PhD (University of Pennsylvania) Martha Combs, BS, MS (University of Pennsylvania); Jade Uffelman, BS (University of Pennsylvania); Loren Terrill (University of Pennsylvania); Oscar Lopez, MD (University of Pittsburgh, Alzheimer's Disease Research Center); Thomas Baumgartner, LSW, MPH (University of Pittsburgh, Alzheimer's Disease Research Center); Sarah Goldberg, LPC (University of Pittsburgh, Alzheimer's Disease Research Center); Donna Simpson, CRNP, MSN, MPH (University of Pittsburgh, Alzheimer's Disease Research Center); Cary Zik, MPH (University of Pittsburgh, Alzheimer's Disease Research Center); Stephen P. Salloway, MD, MS (Butler Hospital Memory and Aging Program); Diane Monast, RN, MSN, CNS (Butler Hospital Memory and Aging Program); Vanessa Rua, RN, BSN (Butler Hospital Memory and Aging Program); Jessica Alber, PhD (Butler Hospital Memory and Aging Program); Athene K.W. Lee, PhD (Butler Hospital Memory and Aging Program); Sophia Tarro (Butler Hospital Memory and Aging Program); Brain R. Ott, MD (Rhode Island Hospital, Retired); Chuang-Kuo Wu, MD, PhD (Rhode Island Hospital); Lori A. Daiello, PharmD, ScM (Rhode Island Hospital); Jonathan D. Drake, MD (Rhode Island Hospital); Alisa Omert, RN (Rhode Island Hospital); Hannah Alaimo (Rhode Island Hospital); Jacobo Mintzer, MD (Ralph H. Johnson VA Health Care System); Olga Brawman-Mintzer, MD (Ralph H. Johnson VA Health Care System); Allison Acree, MS, CHES (Ralph H. Johnson VA Health Care System); Heather Allen, BSN, MS (Ralph H. Johnson VA Health Care System); Arthur Williams, BS, BA (Ralph H. Johnson VA Health Care System); Sydney O'Connor, MA (Baylor College of Medicine); Valory Pavlik, PhD (Baylor College of Medicine); Melissa Yu, MD, FAAN (Baylor College of

Medicine); Shayla Yonce, BA (Baylor College of Medicine); Joseph C. Masdeu, MD, PhD (Nantz National Alzheimer Center, Houston Methodist); Belen Pascual, PhD (Nantz National Alzheimer Center, Houston Methodist); Micha Bangibin (Nantz National Alzheimer Center, Houston Methodist); Benjamin Batista (Nantz National Alzheimer Center, Houston Methodist); Brendan Kelley, MD (University of Texas Southwestern Medical Center); Shahera Ranjha, MS (University of Texas Southwestern Medical Center); Jana Windsor, MS (University of Texas Southwestern Medical Center); Mary Quiceno, MD (University of Texas Southwestern Medical Center, Past Investigator); Elaine Peskind, MD (Seattle Institute for Biomedical & Clinical Research); James O'Connell, MSW (Seattle Institute for Biomedical & Clinical Research); Adam McPartlin, ARNP (Seattle Institute for Biomedical & Clinical Research); Murray A. Raskind, MD, PhD (Seattle Institute for Biomedical & Clinical Research); Anita Ranta, BS (Seattle Institute for Biomedical & Clinical Research); Cynthia M. Carlsson, MD, MS (University of Wisconsin); Benjamin Farral, BS (University of Wisconsin); Kim Peterson, BS (University of Wisconsin); Sandra Harding, MS (University of Wisconsin); Aleshia Cole, RN, APNP (University of Wisconsin); Sarah Best, BSc, CCRP, MHM (Candidate) (Parkwood Institute); Rebecca Shostak, RPN (Parkwood Institute); Kayla VanderPloeg, BScN (Parkwood Institute); Elsa Mann, BScN (Parkwood Institute); Julia Truemner, BA, CCRP (Parkwood Institute); Sandra Black, OC, OOnt, MD, FRCP(C), FRSC, FAAN, FAHA, FANA (Sunnybrook Health Sciences Centre); Benjamin Lam, MD, MSc, FRCP(C) (Sunnybrook Health Sciences Centre); Chinthaka Heyn, PhD, MD, FRCP(C) (Sunnybrook Health Sciences Centre); Samantha Paul-Stotz, MN-RN, BSN (Sunnybrook Health Sciences Centre); Maryna Butenko, MSc (Sunnybrook Health Sciences Centre); Sharon Cohen, MD, FRCPC (Toronto Memory Program); C. Ian Cohen, MD, CCFP (Toronto Memory Program); Atif Shaikh, MBBS, RPN (Toronto Memory Program); Ellen Buchman, MD, CCFP (Toronto Memory Program); Barathy Tharmalingam, CRC (Toronto Memory Program); Linda Schlesinger, BA, CCRP (Toronto Memory Program); Robin Hsiung, MD, MHSc, FRCPC, FACP, FAAN (University of British Columbia, Clinic for Alzheimer Disease and Related Disorders); Ellen Kim, MSc (University of British Columbia, Clinic for Alzheimer Disease and Related Disorders); Tahlee Marian Bpsych (University of British Columbia, Clinic for Alzheimer Disease and Related Disorders); Haakon Nygaard, MD, PhD (University of British Columbia, Clinic for Alzheimer Disease and Related Disorders); Benita Mudge, BSc (University of British Columbia, Clinic for Alzheimer Disease and Related Disorders); Michele Assaly, MA (University of British Columbia, Clinic for Alzheimer Disease and Related Disorders); Colin L. Masters, MD (The University of Melbourne); Andrew Huynh, MBBS, BMedSci, Mclin Tres (The University of Melbourne); Paul Yates, MBBS, PhD, FRACP (The University of Melbourne); Georgios Zisis, RN, MSc (The University of Melbourne); Laura Marginson, RN (The University of Melbourne); Takeshi Iwatsubo, MD, PhD (The University of Tokyo, School of Medicine); Atushi Iwata, MD, PhD (The University of Tokyo, School of Medicine); Kazushi Suzuki, MD, PhD (The University of Tokyo, School of Medicine); Yoshiki Niimi MD, PhD (The University of Tokyo, School of Medicine); Ken-ichiro Sato, MD, PhD (The University of Tokyo, School of Medicine)

###
